# Cryopreserved Human Otic Neuronal Spheroids Self‐assemble for Functional Connectivity Analysis and Long‐term Ototoxicity Evaluation

**DOI:** 10.1002/advs.202505663

**Published:** 2025-11-21

**Authors:** Gaoying Sun, Yukai Wang, Man Wang, Xinyue Wang, Mingming Tang, Da Li, Jianhuan Qi, Xue Wang, Shujuan Sun, Lei Chen, Weibin An, Ligang Kong, Anqi Suo, Haibo Wang, Wenwen Liu, Lei Xu

**Affiliations:** ^1^ Department of Otolaryngology‐Head and Neck Surgery Shandong Provincial ENT Hospital Shandong University Jinan 250022 China; ^2^ Shandong Institute of Otorhinolaryngology Jinan 250022 China; ^3^ Key Laboratory of Organ Regeneration and Reconstruction State Key Laboratory of Stem Cell and Reproductive Biology Institute of Zoology Chinese Academy of Sciences Beijing 100101 China; ^4^ Beijing Institute for Stem Cell and Regenerative Medicine Beijing 100101 China

**Keywords:** electrophysiological assay, human pluripotent stem cell, long‐term ototoxicity evaluation, neural network, spiral ganglion neurons

## Abstract

Spiral ganglion neurons (SGNs) in the inner ear are indispensable for auditory function, and their irreversible damage causes permanent sensorineural hearing loss. Although current human pluripotent stem cell (hPSC)‐derived otic lineages offer a valuable resource for SGN regeneration, they face challenges in terms of reproducibility and functional maturation. Here, a robust protocol is established to generate human otic neuronal spheroids (hONS) from cryopreserved hPSC‐derived pre‐placodal ectoderm (PPE) cells. Post‐thaw PPE cells retained high purity and differentiation efficiency comparable to fresh PPE cells. These self‐assembled hONS differentiated into functionally mature SGN‐like neurons, showing specific maker expression, electrophysiological activity, AMPA receptor‐mediated glutamate response, and extensive neurite extension. In tripartite cocultures incorporating murine cochlear explants and human cortical organoids, hONS formed bidirectional functional synaptic connections, validated through live‐cell imaging, optogenetic stimulation, and synaptic immunostaining. Notably, hONS exhibited heightened sensitivity to ototoxic insults. Short‐term cisplatin exposure induced dose‐dependent alterations in cellular and calcium dynamics, whereas prolonged exposure impaired glutamatergic neural functionality and triggered progressive neuronal death. Co‐treatment with sodium thiosulfate attenuated cisplatin‐induced damage. The hONS model also demonstrated concentration‐dependent toxicity to neomycin. Collectively, this hONS model provides a reliable platform for investigating SGN regeneration and conducting preclinical evaluation of ototoxic drug.

## Graphical abstract

This study establishes a robust and reproducible protocol for generating human otic neuronal spheroids (hONS) from cryopreserved hPSC‐derived pre‐placodal ectoderm cells. These hONS further differentiate into functional SGN‐like neurons, which extend neurite projections toward both murine hair cells and human cortical organoids, thereby forming functional synaptic connections in tripartite coculture systems. Moreover, these hONS demonstrate heightened sensitivity to ototoxic drugs.

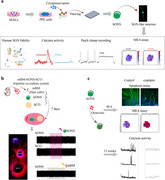

## 1 Introduction

Hearing loss is recognized as the most prevalent sensory disorder globally, with the affected population predicted to reach ≈2 billion by 2050.^[^ ^1^, ^2^ ^]^ This consequently imposes a significant socioeconomic burden on both families and society. Sensorineural hearing loss (SNHL) is the most common form of hearing impairment, primarily caused by irreversible damage to the cochlea hair cells (HCs), the spiral ganglion neurons (SGNs), or both.^[^ ^3^, ^4^, ^5^ ^]^ Mammalian SGNs, which are bipolar neurons situated within the modiolus of cochlea, represent a critical component of the auditory system. These neurons establish synaptic connections with cochlear HCs and play an essential role in sound perception by transmitting auditory signals from the HCs to the cochlear nucleus in the brain.^[^ ^6^, ^7^, ^8^, ^9^ ^]^ During development, SGNs originate from the otic placode, an ectodermal structure derived from the pre‐placodal ectoderm (PPE) that gives rise to the inner ear. The otic placode subsequently invaginates to form the otic vesicle, which then undergoes delamination and differentiation into SGNs following the induction of transcription factors (TFs), including SOX2, EYA1, PAX2, POU4F1, PROX1, NEUROD1, and NEUROG1.^[^ ^8^, ^10^ ^]^ Damage and degeneration of SGNs, which can result from factors such as ototoxic drugs, aging, and noise exposure, not only disrupts the sound transmission and causes difficulties in hearing clarity, speech comprehension, and sound localization, but can also lead to permanent SNHL, as SGNs are not capable of regenerating.^[^ ^11^, ^12^, ^13^, ^14^, ^15^, ^16^, ^17^, ^18^ ^]^ Besides, the cochlear implantation, a common treatment for severe SNHL, works by bypassing damaged HCs and directly stimulating the remaining SGNs, its efficacy depends on the presence of functional SGNs.^[^ ^4^, ^19^, ^20^ ^]^ Nevertheless, the investigation of SGN biology and regeneration currently faces several substantial challenges. First, accessing SGNs is particularly difficult due to their location deep within the cochlea, characterized by small size and intricate structure. Moreover, human cochlear tissue for research purposes is scarce, typically available only postmortem or during surgical procedures such as cochlear implantation. This limitation affects both the quantity and quality of samples, thereby restricting direct studies on human SGNs and necessitating reliance on animal models, which may not fully replicate human auditory physiology or pathology. Consequently, these obstacles have impeded progress in elucidating the role of SGNs in auditory processing and developing effective therapies for hearing loss.

Recently, evidences indicate that introducing exogenous stem cells or inducing the glia‐to‐neuron differentiation of adjacent supporting cells can partially compensate for the injured SGNs in animal models.^[^ ^21^, ^22^, ^23^, ^24^ ^]^ Human pluripotent stem cell (hPSC)‐derived otic systems are particularly noteworthy as they recapitulate otic lineage development, including otic neuron differentiation, and are widely used to model hearing loss in vitro.^[^ ^25^, ^26^, ^27^, ^28^, ^29^ ^]^ However, current protocols for differentiating otic neurons from hPSC sources are not yet suitable for translational applications, as they depend heavily on the initial cell population and cannot be reliably replicated across different research groups. Inconsistent batch‐to‐batch performance is a problem that may have contributed to past clinical failures in neural transplantation.^[^ ^30^, ^31^, ^32^, ^33^, ^34^ ^]^ Additionally, deriving mature otic neurons from hPSCs is a lengthy process that must be carried out to completion, which may result in neuronal heterogeneity. To address this, cryopreservation, which maintains the structural and functional integrity of cells at ultralow temperatures, can provide a steady supply of quality‐controlled, ready‐to‐use cells for screening, thereby avoiding extended continuous cultures.^[^ ^35^ ^]^ The translational potential of cryopreserved hPSC‐derived precursors has already been demonstrated. For example, cryopreserved midbrain dopamine precursors derived from hPSCs have been shown to efficiently yield A9 midbrain dopamine neurons in vivo and ameliorate phenotypes in rat models of Parkinson's disease.^[^ ^30^, ^31^ ^]^ Additionally, the cryopreservation of hPSC‐derived mid‐hindgut endoderm monolayers has enabled reproducible and scalable production of small intestinal organoids without the need for *de novo* differentiation of hPSCs.^[^ ^36^ ^]^ In this study, we hypothesized that a cryopreservation strategy could be established for hPSC‐derived otic progenitor cells, facilitating the reproducible generation of human otic neuronal spheroids (hONS) and otic neurons independent of the original hPSCs.

Neurons display distinct electrophysiological characteristics during maturation, which are essential for electrical signaling in diverse biological contexts. In the auditory system, the glutamatergic synapse between inner hair cells (IHCs) and SGNs is highly specialized, enabling sustained afferent transmission at frequencies exceeding hundreds of Hertz with sub‐millisecond precision.^[^ ^10^, ^37^ ^]^ Upon sound stimulation, calcium influx through voltage‐gated calcium channels in IHCs triggers the release of glutamate into the synaptic cleft. The binding of glutamate to postsynaptic receptors on SGNs then triggers depolarization, leading to action potential generation and propagation of auditory signals to the brainstem.^[^ ^38^, ^39^ ^]^ Among these receptors, AMPA receptors mediate rapid excitatory transmission and serve as the primary driver of action potential generation, whereas NMDA receptors, expressed at relatively low levels in mature SGNs, activate more slowly and are implicated in synaptic plasticity and long‐term modulation. Therefore, a physiologically relevant in vitro model of human SGNs should recapitulate both glutamatergic properties and calcium‐mediated electrophysiological activity to faithfully model their in vivo counterparts. In this study, we established a cryopreservation protocol for hPSC‐derived PPE cells and systematically demonstrated their capacity to form self‐organizing, highly reproducible hONS. Within these 3D structures, functional SGN‐like neurons efficiently differentiated, exhibiting mature electrophysiological activity, robust calcium dynamics, and characteristic bipolar morphology. In coculture systems, these neurons extended neural projections toward both murine HCs and human cortical organoids, demonstrating potential for synaptic integration. Furthermore, we evaluated the response of hONS to short‐ and long‐term ototoxic injury, highlighting their utility as a sensitive platform for ototoxicity testing and mechanistic study. Our findings provide key insights and a scalable model for developing therapeutic strategies aimed at SGN regeneration and the treatment of SNHL.

## 2 Results

### 2.1 Generation of Self‐Organizing and Reproducible Otic Neuronal Spheroids Using Cryopreserved hPSC‐Derived PPE Cells

**Figure 1:**
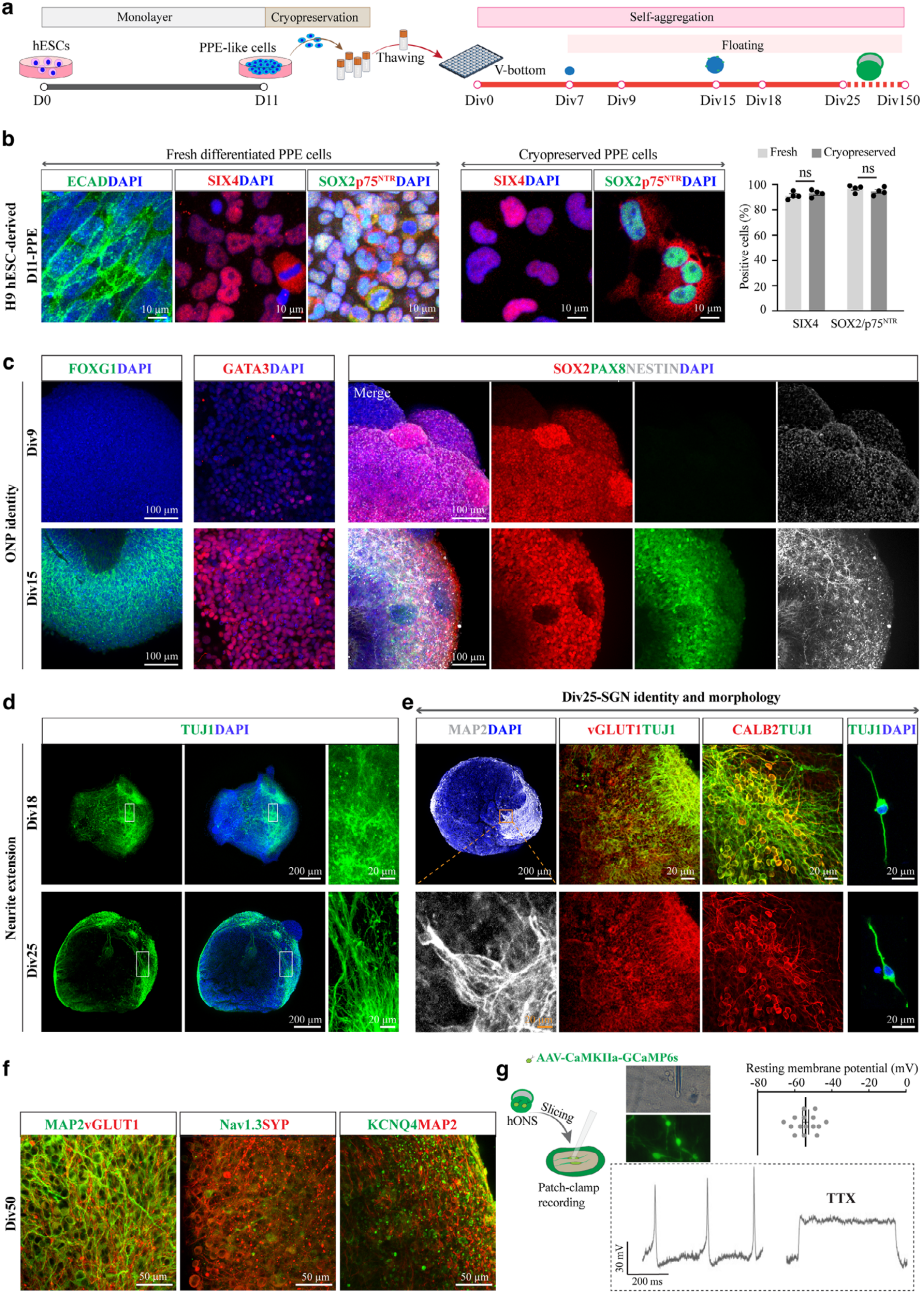
Generation of self‐organizing and reproducible hONS using cryopreserved hPSC‐derived PPE cells. a) Schematic diagram of a stepwise differentiation protocol for obtaining hONS from hESCs. Div, day in vitro. b) Representative immunostaining images of D11 cells for PPE markers, ECAD, SOX2, p75^NTR^, and SIX4 and quantification analysis of SIX4^+^ and SOX2/p75^NTR+^ cells. Unpaired two‐tailed Student's *t*‐test, *n* = 4 spheroids for each group. c) Representative immunostaining of otic neuronal progenitor (ONP) markers, GATA3, NESTIN, SOX2, and PAX8. d) Immunostaining of neuronal marker TUJ1 to show neurite extension in spheroids from different time points. e) Immunostaining of mature neuronal marker MAP2, glutamatergic marker vGLUT1, and SGN marker CALB2, in Div25 spheroids and bipolar neurons of dissociated Div25 spheroids. f) Immunostaining of mature glutamatergic neurons (MAP2 and vGLUT1), and ion channel proteins, Nav1.3 and KCNQ4, at Div50 H9 hONS. g) Overview of whole‐cell patch‐clamp recording and resting membrane potentials (RMPs) of CaMKII‐labelled glutamatergic neurons in hONS, n = 15 cells. Bottom, representative action potentials of glutamatergic neurons from hONS before or after TTX administration. Values are presented as mean ± SEM. ns, no significance.

Given that the PPE is a crucial embryonic structure that gives rise to otic placodes, PPE‐like cells derived from hPSCs are being explored for their potential in inner ear regeneration and cochlear implant optimization.^[^ ^25^, ^28^ ^]^ Previously, we used H9 hESCs to generate PPE‐like cells, which subsequently differentiated into otic neuronal organoids majoring in SGN‐like cells.^[^ ^28^ ^]^ In this study, we chose to cryopreserve H9 hESCs‐derived PPE cells and tested whether they can be stably induced into otic placodes containing otic neuron precursors in vitro (**Figures** **1**a and S1a,b, Supporting Information). Prior to cryopreservation, the cellular identity was validated through immunostaining for PPE markers, including ECAD, SOX2, p75^NTR^, and SIX4. Results demonstrated that by day 11 (D11) of differentiation, monolayer cells derived from H9 hESCs were positively labelled with all markers, with 96.79% ± 1.38% of cells co‐expressing SOX2 and p75^NTR^, and 91.23% ± 1.47% expressing SIX4 (Figure 1b), indicating successful induction of PPE cells. These cells, designated as fresh differentiated PPE cells, were dissociated into single cells and cryopreserved for subsequent experimental analysis.

We next assessed the viability and differentiation potential of cryopreserved PPE cells following thawing. Analysis of cellular purity revealed that the thawed cells maintained high‐level expression of PPE markers, with 94.56% ± 1.52% of cells co‐expressing SOX2 and p75^NTR^, and 93.01% ± 1.36% positive for SIX4 (Figure 1b). The thawed cells were resuspended and seeded into low‐adhesion, V‐shaped 96‐well plates for 3D differentiation, with the seeding day designated as day in vitro 0 (Div0) (Figure 1a). After four days of self‐aggregation, single cells formed round spheroids, which continued to increase in size over time (Figure S1c,d, Supporting Information). By Div9, the spheroids exhibited positive expression of early otic markers GATA3 and JAG1, but were negative for otic neuronal progenitor (ONP) markers such as FOXG1, PAX8, and PAX2 (Figure 1c and Figure S2a, Supporting Information). Consistently, minimal expression of NESTIN, a marker of neuronal progenitors, was detected by Div9 (Figure 1c), indicating that the spheroids were at an early ONP stage. By Div15, spheroids derived from cryopreserved PPE cells began to express late ONP markers, including SOX2, PAX8, PAX2, FOXG1, and NESTIN (Figure 1c), mirroring the differentiation timeline observed in spheroids generated via the conventional non‐cryopreserved (fresh) approach (Figure S2b, Supporting Information). These results confirm that cryopreserved PPE cells retain their capacity to efficiently differentiate into ONPs.

Following the ONP stage, cells progressed toward neuronal maturation. Our results showed that, after Div15, the size of the spheroids stabilized (Figure S1c,d, Supporting Information). By Div25, the spheroids exhibited neurite extensions and connections between adjacent neural clusters, a feature not observed at Div18 or earlier stages (Figure 1d). Additionally, Div25 spheroids expressed markers indicative of mature neurons, including the glutamatergic marker vGLUT1, the mature neuron marker MAP2, and the SGN marker CALB2 (Figure 1e). Morphological analysis of dissociated spheroids revealed the characteristic bipolar architecture of the neurons (Figure 1e). We also detected the expression of key TFs specific to the early SGN lineage, including POU4F1, PROX1, and NEUROD1, both in fresh and cryopreserved groups (Figure S2c, Supporting Information). Statistical analysis revealed no significant differences between the two groups with respect to the proportion of TF‐positive cells or their corresponding mRNA expression levels (Figure S2c,d, Supporting Information). Furthermore, S100B‐positive glial cells were observed in Div25 spheroids across both groups (Figure S2e, Supporting Information). Collectively, these findings indicate that by Div25, the spheroids have developed a heterogeneous population comprising both mature SGN‐like neurons and glial cells.

By Div50, hONS from both fresh and cryopreserved groups exhibited further structural complexity, with more elaborated neurite outgrowth indicative of advanced neuronal maturation (Figure 1f and Figure S2f,g. Supporting Information). Using immunostaining for the synaptic vesicle marker synaptophysin (SYP) and the neuronal marker MAP2, we quantified synaptic vesicle density, defined as SYP‐positive puncta per 100 µm of MAP2‐labeled neurite, and found no significant differences between the fresh and cryopreserved groups (Figure S2g, Supporting Information). To evaluate functional maturation, we assessed the expression of voltage‐gated ion channels Nav1.3 and KCNQ4, both of which showed clear co‐localization with SYP and MAP2 (Figure 1f). Whole‐cell patch‐clamp recordings were performed on spheroid slices at Div50 to detect the electrophysiological properties of derived‐neurons. Prior to recording, glutamatergic neurons were selectively labeled using AAV‐CaMKIIa‐GCaMP6s, which expressed the calcium indicator GCaMP6s under the CaMKIIa promoter (Figure 1g). The recorded neurons exhibited a mean resting membrane potential of –54.07 ± 1.63 mV, consistent with values reported in previous studies of mature human otic neurons cultured with neurotrophic factors.^[^ ^27^ ^]^ Moreover, tetrodotoxin (TTX)‐sensitive action potentials in response to depolarizing current were elicited in most recorded cells (n = 12/15, Figure 1g). These results indicate that these otic spheroids‐derived neurons not only express key voltage‐gated channels but also display functional electrophysiological properties characteristic of mature glutamatergic neurons.

Furthermore, to evaluate the reproducibility of our protocol, we extended our validation to an additional hESC line (H1). Cryopreserved H1 hESC‐derived PPE cells also exhibited high purity, with 93.46% ± 1.31% SIX4‐positive and 94.50% ± 1.85% SOX2 and p75^NTR^‐double positive cells (Figure S2h, Supporting Information), and efficiently gave rise to well‐structured, mature glutamatergic neurons within hONS by Div50 (Figure S2i, Supporting Information). We analyzed organoid formation rates from cryopreserved PPE cells derived from two hESC lines (H9 and H1). Across multiple independent batches and thawed vials, the spheroid formation rates consistently exceeded 96.88% (Table S3, Supporting Information), matching the efficiency observed in fresh PPE‐derived groups (over 97.22%, Table S3, Supporting Information). These results demonstrate the reliability and cell line‐independent robustness of the cryopreservation protocol for generating otic spheroids. Besides, to assess batch‐to‐batch consistency, we generated spheroids from two independent batches of H9 hESC‐derived cryopreserved PPE cells and observed highly similar transcriptomic profiles at both Div0 and Div15 (Figure S3a,b, Supporting Information), along with consistent expression patterns of genes related to otic identity, proliferation, and pluripotency (Figure S3c, Supporting Information). Comparative gene expression analysis of key otic markers (*GATA3*, *GATA2*, *EYA1*, *DLX3*) revealed no significant differences between fresh and cryopreserved samples at both Div0 and Div15 (Figure S3d, Supporting Information). Collectively, these results demonstrate that our cryopreservation‐based method reliably produces otic spheroids independent of the hPSC line or batch used, underscoring its stability and reproducibility.

### 2.2 Transcriptomic Analysis Unravels Cell Fidelity to SGN in Human Fetal Inner Ear Atlas

To evaluate the maturity of SGN‐like cells within our hONS, we performed bulk RNA‐seq at Div50, a stage associated with functionally mature glutamatergic neurons, on spheroids generated from both fresh and cryopreserved approaches. Principal component analysis (PCA) and clustering heatmaps revealed high transcriptional similarity between fresh and cryopreserved groups across both H9 and H1 hESC lines (Figure S3e,f, Supporting Information). Expression heatmaps further demonstrated enriched transcription of markers associated with mature SGNs, ion channels, and genes implicated in spontaneous neuronal firing in the compared groups across two hESC lines (Figure S3g, Supporting Information). We additionally validated the elevated expression of selected voltage‐gated channel genes (*KCNA5*, *KCNJ8*, *KCNQ2*, *SCN3A*, *SCN9A*) via RT‐PCR in Div50 hONS from both conditions and cell lines (Figure S3h, Supporting Information). Together with whole‐cell patch‐clamp electrophysiological profiles, these molecular data confirm the functional maturity of neurons within the hONS.

**Figure 2:**
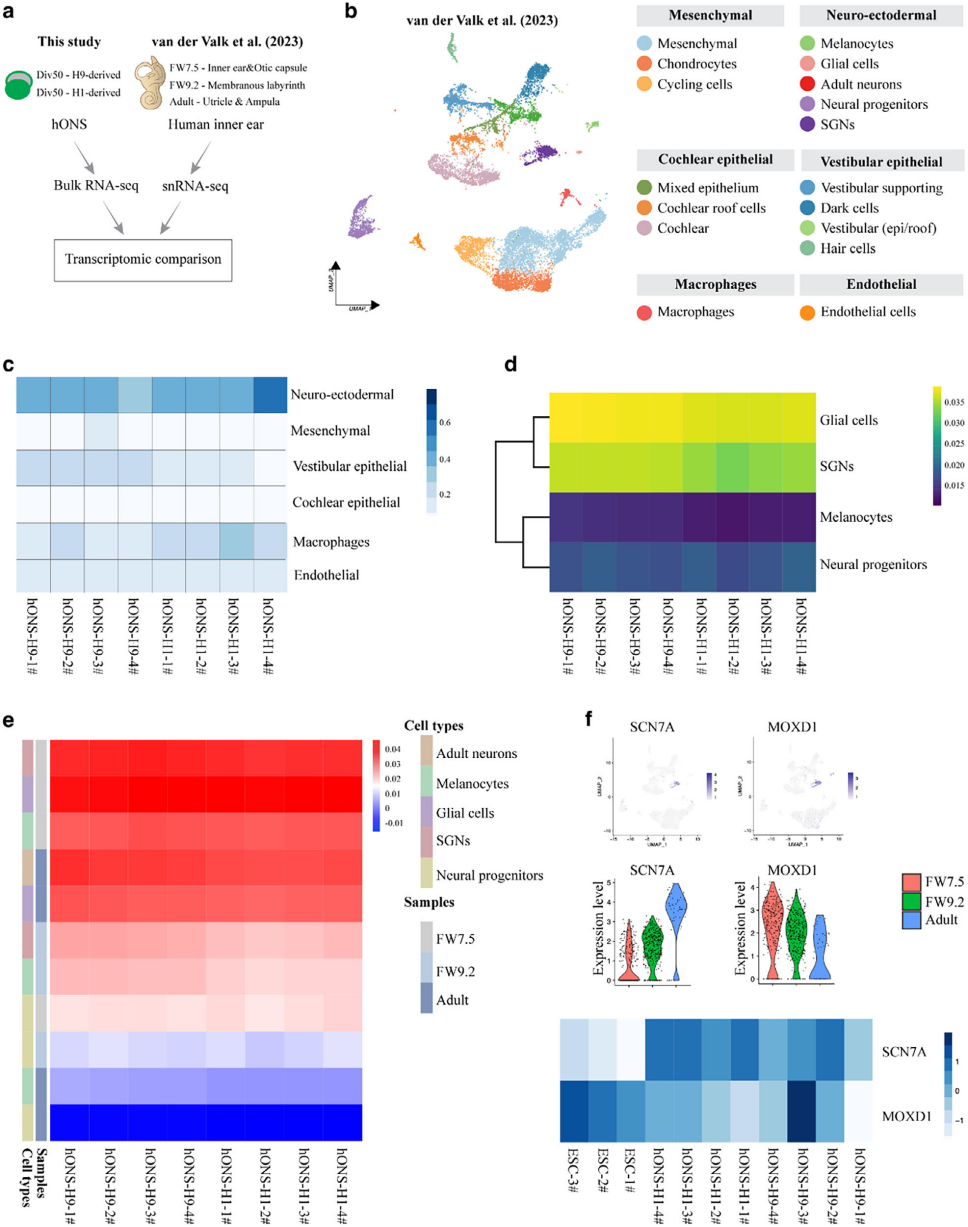
Transcriptomic analysis unravels cell fidelity to SGNs in the human fetal inner ear atlas. a) Experimental overview of transcriptomic comparison between the generated hONS and the published human inner ear atlas.^[^ ^40^ ^]^ Each sample contains 1 spheroid, n = 4 spheroids for each hESC line. b) Cell type annotations of integrated human inner ear datasets using uniform manifold approximation and projection (UMAP) plot. c) Heatmap showing cell type abundance analysis in hONS bulk RNA‐seq using BayesPrism algorithm. The heatmap displays the relative abundance of major cell types identified in the inner ear atlas across different samples, revealing that neuro‐ectodermal cells are the predominant component of the hONS. d) Heatmap showing gene expression correlation between hONS bulk RNA‐seq and pseudobulk profiles of four neuro‐ectodermal subtypes identified in the inner ear single‐cell atlas. e) Heatmap showing gene expression correlation between hONS bulk RNA‐seq and pseudobulk profiles of neuro‐ectodermal subtypes across three developmental timepoints (Fetal_75, Fetal_92, and Adult) from the inner ear single‐cell atlas. f) Heatmap, FeaturePlot, and VlnPlot showing SCN7A and MOXD1 expression in hONS and adult neurons across three developmental timepoints (Fetal_75, Fetal_92, and Adult) from the inner ear single‐cell atlas.

To assess the fidelity of our hONS in recapitulating the characteristics of native human SGNs, we performed a comparative transcriptomic analysis using a reference single‐nucleus RNA sequencing atlas of the human inner ear published by van der Valk et al. (**Figure** **2**a).^[^ ^40^ ^]^ After integrating fetal (7.5‐ and 9.2‐week) and adult snRNA‐seq datasets, we re‐annotated cell clusters using known markers, identifying five major neuroectodermal populations: melanocytes, glial cells, adult neurons, neural progenitors, and SGNs (Figures 2b and S3j,k, Supporting Information). Deconvolution analysis using BayesPrism indicated that neuroectodermal cell types constituted the largest fraction within the hONS (Figures 2c and S3l, Supporting Information). Correlation analysis between pseudobulk profiles of neuroectodermal subclusters and our bulk RNA‐seq data revealed significant transcriptional similarity between Div50 hONS and human inner ear melanocytes, glial cells, and SGNs (Figures 2d and S3m, Supporting Information). Further correlation analysis confirmed that hONS exhibit high similarity to three specific cell populations within the fetal week 7.5 neuroectodermal group (Figure 2e). Consistent with this, key marker genes of SGNs such as *SCN7A* and *MOXD1*, which are highly expressed in fetal datasets, were also prominently expressed in our hONS (Figures 2f and S3n, Supporting Information). Collectively, these findings demonstrate that hONS, generated via both fresh and cryopreserved protocols from two independent hESC lines, exhibit transcriptional congruence with glial, spiral ganglion neuronal, and melanocyte populations within the fetal human inner ear neuroectoderm, providing evidence for the fidelity of our hONS model in recapitulating authentic human SGN identity.

Furthermore, we compared the heterogeneity of PPE‐derived hONS with non‐PPE‐derived hONS from H9 ESCs using our previously established differentiation protocol.^[^ ^29^ ^]^ At the ONP stage, PPE‐derived hONS had a significantly higher percentage of PAX2‐positive cells than the non‐PPE group (91.85% ± 4.14% vs 43.85% ± 2.06%; Figure S10a, Supporting Information). Bulk RNA‐seq analysis uncovered distinct transcriptional profiles between the two groups (Figure S10b, Supporting Information). PPE‐derived hONS showed marked upregulation of SGN marker genes and downregulation of glial marker genes (Figure S10c, Supporting Information). GO and KEGG analyses revealed that PPE‐derived hONS were enriched in neuronal development and maturation‐related terms, including neurogenesis, neuron differentiation, axon guidance, calcium signaling pathway, glutamatergic synapse, relative to non‐PPE‐derived hONS (Figure S10d,e, Supporting Information). These findings indicate that PPE‐derived hONS exhibit reduced heterogeneity and enhanced specificity in SGN lineage development compared to non‐PPE‐derived hONS.

### 2.3 hONS Develop Stable and Glutamate‐Responsive Calcium Activity

**Figure 3:**
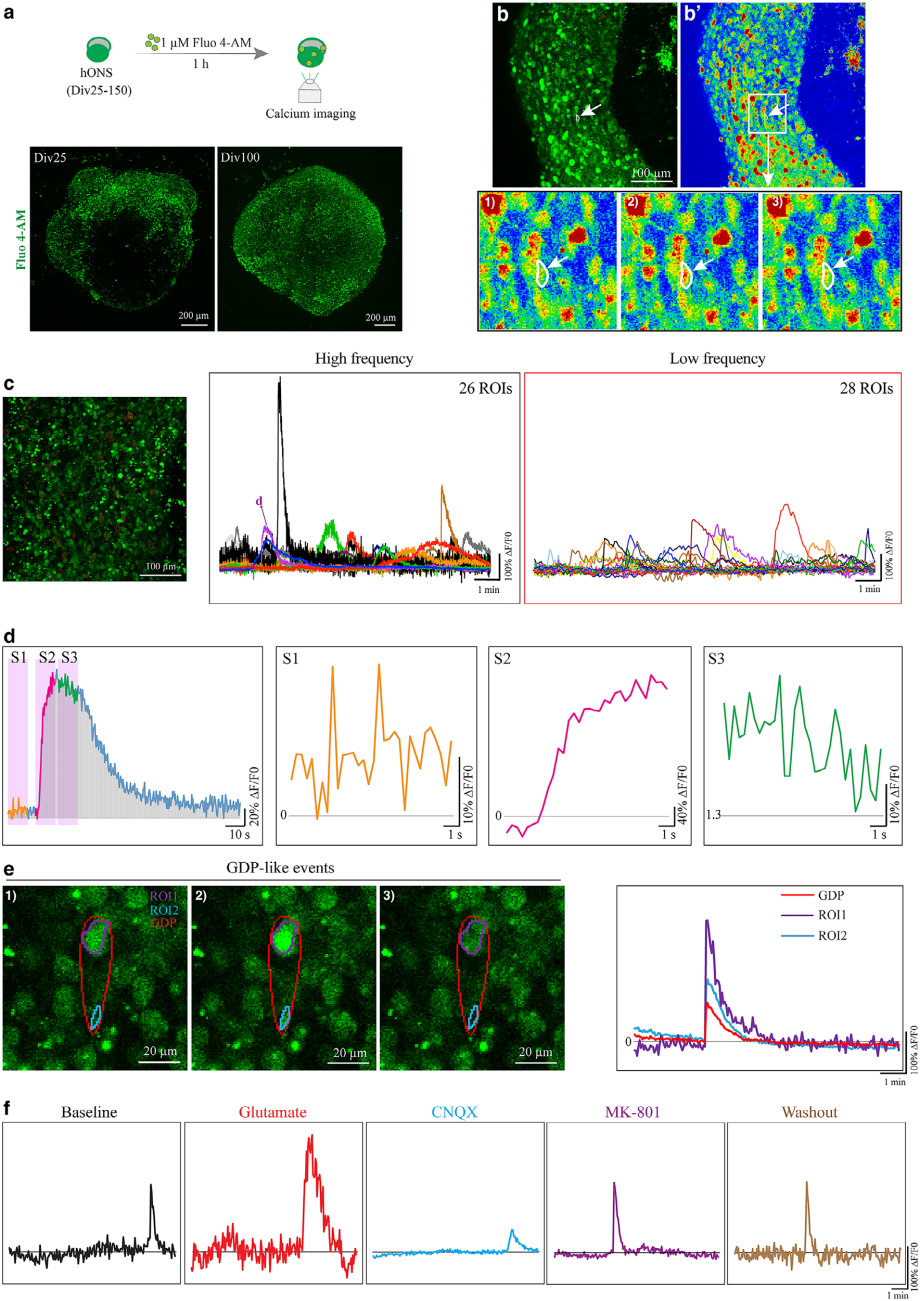
hONS develop stable and glutamate‐responsive calcium activity. a) Calcium imaging analysis of otic spheroids after Div25. Up, schematic diagram. Bottom, representative images of whole spheroids loaded with the calcium indicator Fluo 4‐AM. b) Heatmaps of a Fluo‐4 AM‐loaded otic spheroid. Up, the snapshot overview of calcium imaging at one sampling timepoint. Bottom, three consecutive heatmaps of the boxed area to show the dynamic changes of calcium signals in the white‐line circled region of interest (ROI). c) Colored calcium traces (∆F/F0) of ROIs detected in a Fluo 4‐AM‐labeled otic spheroid at high and low sampling frequencies, respectively. d) Details of a single calcium trace indicated in c at baseline (S1), shift (S2), and peak (S3) phases of high sampling frequency. e) Calcium images and colored‐calcium traces showing a representative giant depolarized potential (GDP)‐like event detected from a Fluo 4‐loaded otic spheroid. f) Calcium traces of the same ROI under different conditions. Glutamate, a robust stimulator of glutamatergic neurons. CNQX, the inhibitor of AMPA receptors. MK‐801, an antagonist of NMDA receptors.

Calcium activity in cochlear SGNs is critical for their function in auditory signal transmission. Here, we employed calcium dye‐based imaging to investigate the calcium activities of spheroids after Div25, when cells have developed a bipolar morphology (**Figure** **3**a). Active calcium responsiveness was detected in spheroids after Div25, as evidenced by dynamic heatmaps and consistent patterns of calcium transients detected in the same calcium dye‐loaded spheroids imaged at both high and low sampling frequencies (Figure 3a–c). These calcium‐responsive regions exhibited robust activity even at the baseline (S1 section) and peak (S3 section) phases of individual calcium traces, which displayed pronounced calcium fluctuations (S1 and S3 lines). These findings suggest that the neurons within the analyzed spheroids are functionally active (Figure 3d). Furthermore, giant depolarized potential (GDP)‐like events, which are indicative of active neurons in calcium activity assay, were spontaneously detected in these spheroids (Figure 3e).

Cochlea SGNs are glutamatergic neurons. To further investigate the glutamatergic property of hONS, we examined their responses to glutamate, a glutamatergic neuron‐specific stimulant. Results indicated that glutamate stimulation significantly increased the ∆F/F0 ratio of the same region of interest (ROI) within the detected spheroids (Figure 3f), corroborating the glutamatergic property. To clarify which glutamatergic receptors contribute for the glutamatergic phenotype, CNQX (an antagonist of AMPA receptors) or MK‐801 (an antagonist of NMDA receptors) was applied separately to specifically inhibit corresponding receptors. The same ROI showed specific inhibitory responses to CNQX, which were reversible upon washout, while MK‐801 had no detectable inhibitory effect (Figure 3f). Additionally, RT‐PCR confirmed high mRNA expression of AMPA receptors, *GRIA1* and *GRIA2*, and no detectable expression of NMDA receptor *GRIN3B* at Div50 hONS (Figure S4a, Supporting Information). These results collectively demonstrate that the glutamatergic phenotype is mediated by AMPA receptors within the spheroids.

### 2.4 Neurite‐Sprouted Otic Neuronal Spheroids Exhibit Glutamatergic Neural Network

**Figure 4:**
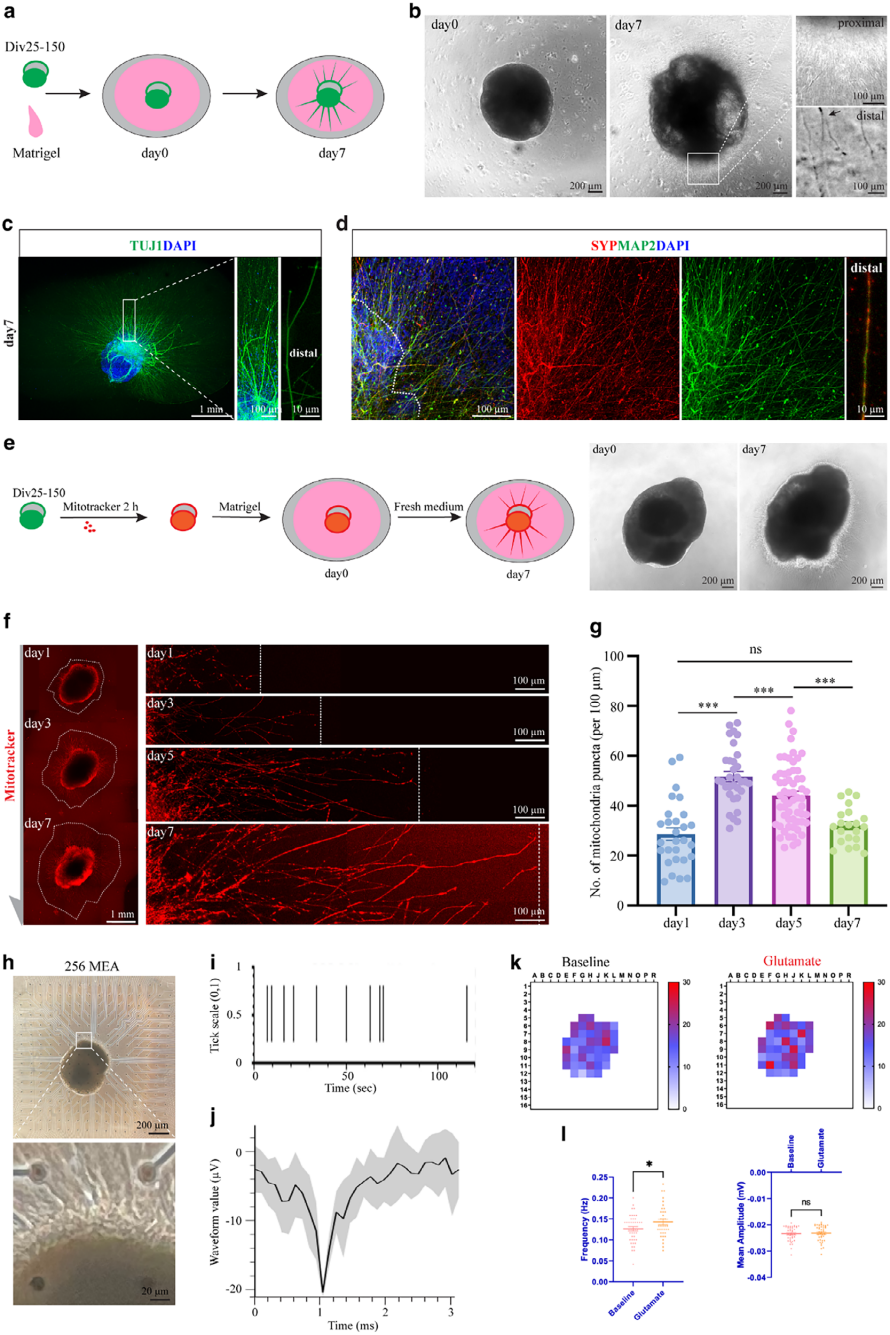
Neurite‐sprouted hONS exhibit a glutamatergic neural network. a) Schematic diagram of neurite extension assay. b) Phase control images of a Matrigel‐encapsuled otic neuronal spheroid. Close‐up views showing the proximal and distal sections of extended neurites. c,d) Representative immunostaining images of neurons (TUJ1^+^MAP2^+^), synaptic protein (SYP^+^) in Matrigel‐encapsuled otic spheroids. e) Experimental design of cell viability assay using live mitochondria dye. f,g) Dynamics of extended neurites in Mitotracker‐loaded hONS encapsuled in Matrigel over time. One‐way ANOVA followed by Tukey's test, n = 21 to 60 neurites from 5 spheroids for each group. h) Phase control images showing a Div40 hONS mounted on a 256‐electrode MEA plate. i,j) Frequency raster plot (i), and stacked amplitude waves (j) of spikes detected from single electrodes. k,l) Frequency heatmaps (k) and quantification (l) of spikes detected from the otic spheroid after glutamatergic stimulation. Unpaired two‐tailed Student's *t*‐test, n = 36 (Baseline) and 43 (Glutamate) active electrodes for frequency quantification. n = 41 (Baseline) and 42 (Glutamate) active electrodes for amplitude quantification. Values are presented as mean ± SEM. *p < 0.05, ***p < 0.001, ns, no significance.

The extension and guidance of SGN neurites are crucial to construct connections between IHCs and auditory nuclei. We introduced a commercially available biomaterial matrix, Matrigel, as a 3D scaffold to support neurite extension of hONS (**Figure** **4**a). The spheroids after Div25 were suspended in pure Matrigel. After seven days of culture, the encapsulated spheroids presented robust fasciculation proximally with numerous branches at the distal ends and high expression of synaptic protein, indicating an advanced neuronal extension development (Figure 4b–d). We then assessed the viability of the extended neurites using MitoTracker, a dye that labels mitochondria in live cells (Figure 4e). From day 1 to day 7 of culture, an increasing number of neurites emerged from the spheroids over time, all of which exhibited strong MitoTracker staining, indicating the presence of live and abundant mitochondria (Figure 4f). Statistical analysis revealed that the density of mitochondria in the extended neurites significantly increased on days 3 and 5 compared to day 1, with the highest density observed on day 3. However, by day 7, the mitochondrial density declined, showing no significant difference compared to day 1 (Figure 4g).

Next, we investigated the electrophysiological activity of the neurite‐sprouted spheroids using a 256‐electrode microelectrode array (MEA) system (Figure 4h). This system enabled high‐throughput assessment of the electrophysiological profiles of the loaded spheroids under both baseline and glutamate‐stimulated conditions. Typical spikes and amplitudes characteristic of mature neurons were detected from active electrodes in both fresh and cryopreserved groups derived from H9 hESC line (Figure 4i,j and Figure S4c, Supporting Information), as well as in the cryopreserved group derived from H1 hESC line (Figure S4e, Supporting Information), suggesting the maturation of neurons within the otic neuronal spheroids. Glutamate stimulation significantly increased the firing rate, indicating the presence of functional glutamatergic neurons (Figure 4k,l and Figure S4a–c, Supporting Information). Importantly, no significant differences in spike amplitude or frequency responses to glutamate were observed between the cryopreserved and fresh groups (Figure S4d, Supporting Information), supporting functional equivalence between the two groups. Taken together, embedding mature otic neuronal spheroids in Matrigel recapitulates the physiological neurite sprouting of otic neurons in vitro and facilitates the development of a glutamatergic neural network within the spheroid system.

### 2.5 hONS Form Functional Glutamatergic Neural Projection Bidirectionally with Peripheral and Central Auditory Components

**Figure 5:**
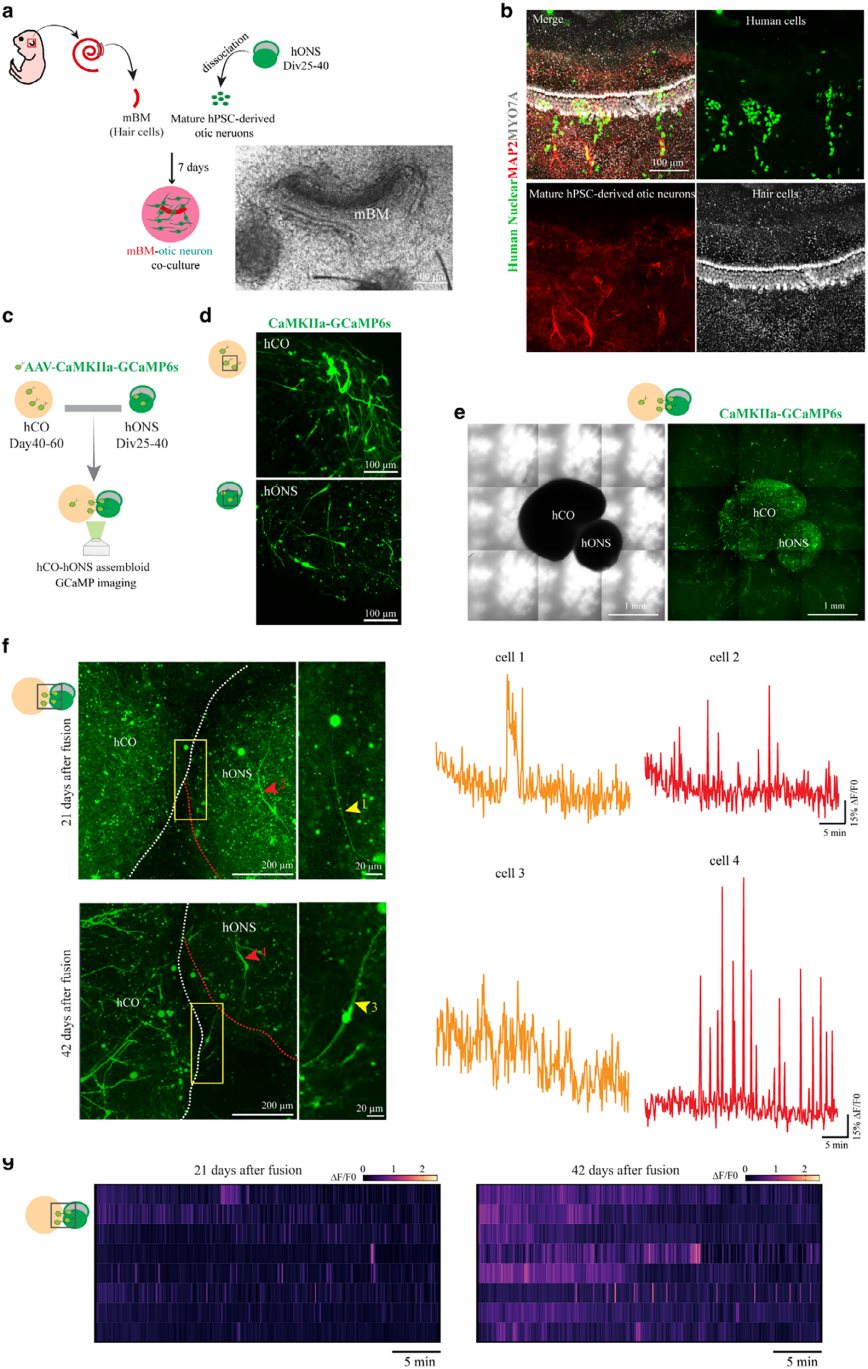
hONS form glutamatergic neural projection with peripheral and central auditory components. a) Schematic diagram presenting the coculture of murine basilar membrane (mBM, incorporating HCs) explant and human mature otic neurons (dissociated otic spheroids between Div25‐40). b) Immunostaining of the mBM‐otic neuron coculture system showing the neural innervation between mature hPSC‐derived otic neurons (humanNuclear^+^MAP2^+^) and HCs (MYO7A^+^). c) Schematic diagram showing the generation of human cortical organoid (hCO)‐otic spheroid assembloid. d) The CaMKIIa‐GCaMP6s‐labeled hCO and otic spheroids before fusion. e) The overview images, phase and confocal, of an hCO‐otic spheroid assembloid after 21 days of fusion. f) Left, the calcium imaging of glutamatergic neurons around the connection of the assembliod in e fused for different days. Yellow and red arrowheads indicate bipolar glutamatergic neurons at different positions, respectively. Right, Calcium traces of neurons indicated. g) Frequency heatmaps of glutamatergic neurons at the connection site in the assembloid fused for different days.

SGNs extend peripheral processes toward cochlear HCs and central axons toward the cochlear nucleus in the brainstem, therefore bridging HCs and the cochlear nucleus for electrical signal transmission. To simulate the peripheral projection, given the impracticality of using the human cochlear basilar membrane, we constructed a coculture system comprising the middle turn of the murine basilar membrane (mBM), which contains well‐organized HCs, and mature otic neurons (**Figure** **5**a). The hONS were dissociated into single cells and evenly seeded onto cover‐slips pre‐loaded with mBM explants. As illustrated in Figure 4b, the human otic neurons, marked with the antibodies of human nuclear and MAP2, formed clusters and were predominantly localized near MYO7A‐labeled HCs, indicating the capability of otic neurons to establish peripheral projections toward (Figure 5a,b).

To simulate central projection, we cocultured hPSC‐derived cortical organoids (hCOs) (Figure S5a, Supporting Information), as the human cerebral component, with hONS. Both of the organoids were pre‐transduced with AAV‐CaMKII‐GCaMP6s to enable real‐time calcium imaging (Figure 5c). While hCO neurons exhibited multipolar morphologies, hONS‐derived neurons retained a bipolar phenotype, confirming their distinct origin (Figure 5d). After 21 days, fused assembloids showed bipolar glutamatergic neurons bridging hCO and otic spheroids (Figure 5e). These neurons exhibited unique calcium dynamics at fusion sites, suggesting functional interaction with hCOs (Figure 5f). This connectivity remained stable for up to three months, confirming robust integration.

**Figure 6:**
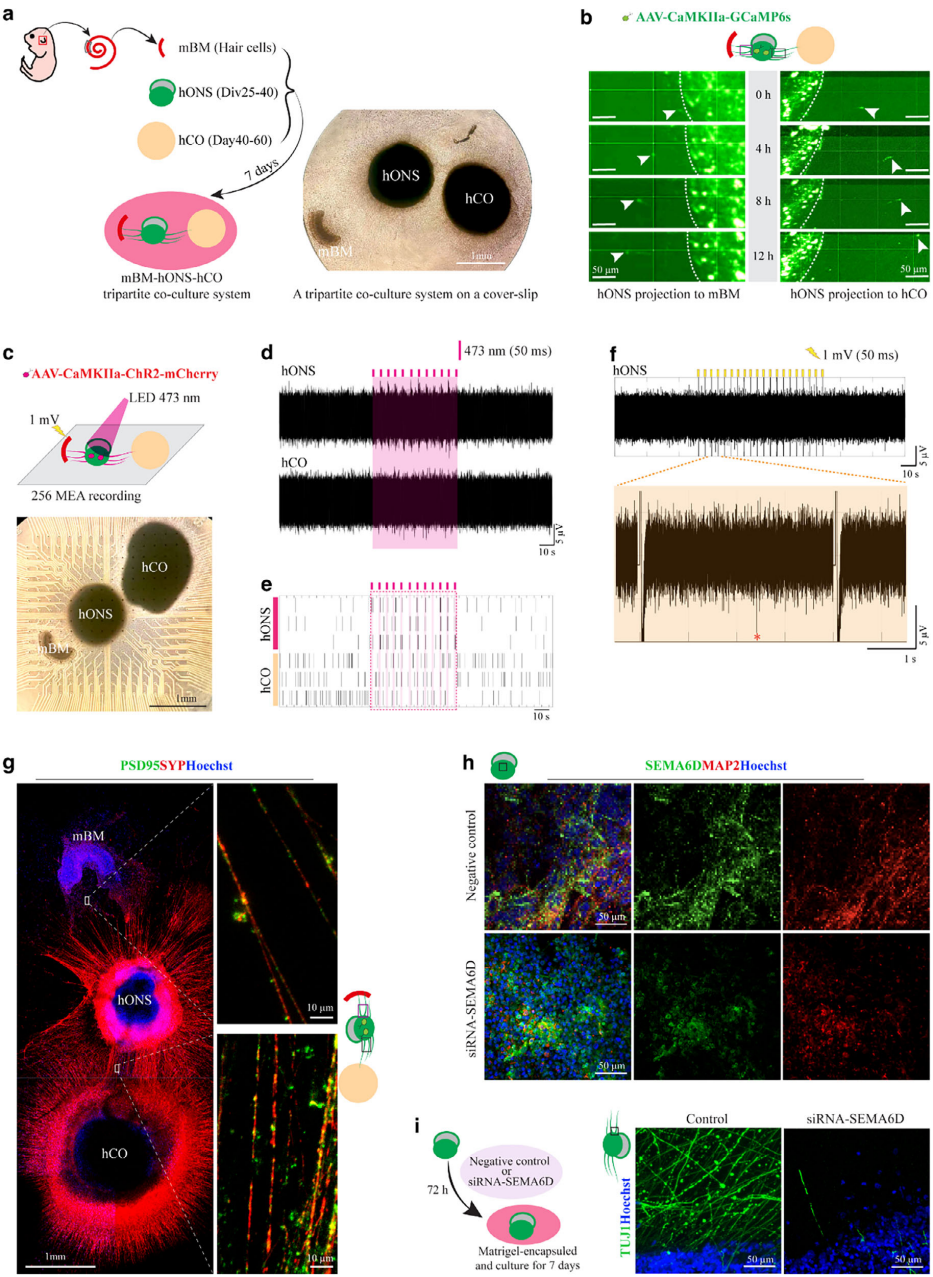
hONS form functional glutamatergic neural projection bidirectionally with peripheral and central auditory components. a) Schematic diagram presenting the tripartite coculture of mBM explant, hONS (Div25‐40), and hCOs (Day 40‐60). b) Time‐lapse imaging showing the processes of neurons from AAV‐CaMKIIa‐GCaMP6s‐labeled hONS in a tripartite coculture system. c) Optogenetic stimulation integrated with MEA assay of a tripartite coculture system expressing AAV‐CaMKIIa‐ChR2‐mCherry in otic spheroids. d,e) Representative firing profiles (d) and frequency raster plots (e) of electrodes from hONS and hCO, stimulated by 473‐nm LED light. f) Representative spikes firing of electrodes in hONS after electrical stimulation of BM electrodes. g) Representative immunostaining showing the pre‐synaptic (SYP) and post‐synaptic protein (PSD95) at the junction site of the coculture system. h) Immunostaining of SEMA6D and MAP2 at Div50 hONS (H9 and cryopreserved approach) in different groups. i) siRNA‐mediated knockdown of SEMA6D leads to neglectful neural processes extended from hONS.

We further constructed a tripartite coculture system comprising GCaMP6s‐labeled hONS, hCO, and mBM to dynamically visualize neurite projection (**Figure** **6**a). Time‐lapse imaging revealed neurite extension from hONS toward both targets within 24 h, with penetration into hCO and mBM tissues by 60 h (Figure 6b and Videos S1,S2, Supporting Information). Functional connectivity was assessed using a combination of optogenetic stimulation and MEA recording. Prior to assembly, hONS were transduced with AAV‐CaMKIIa‐ChR2‐mCherry to drive neuron‐specific expression of the light‐sensitive channelrhodopsin ChR2 (Figure S5b, Supporting Information). These modified hONS were subsequently cocultured with hCO and mBM for 7 days to facilitate integration (Figure 6c). Optical stimulation of hONS with 50‐ms pulse of 473‐nm light elicited a significant increase in firing rate in hCO‐proximal electrodes, with a response latency of ≈100 ms (Figure 6d,e). Reciprocally, electrical stimulation of mBM‐adjacent electrodes reliably induced firing in hONS‐proximal electrodes (Figure 6f), demonstrating bidirectional communication. Furthermore, immunostaining revealed co‐localization of the pre‐synaptic marker SYP and post‐synaptic marker PSD95 at hONS‐target interfaces (Figure 6g), validating the structural integrity of these synapses. Together, these results confirm the establishment of functional bidirectional synaptic transmission between hONS and both central (hCO) and peripheral (mBM) auditory components.

To identify potential molecular cues governing hONS neurite targeting, we performed GO enrichment analysis on bulk RNA‐seq data from Div50 hONS (vs PPE cells). GO terms associated with axon guidance, axon extension, neuron projection, and axonogenesis were significantly enriched in hONS, which is consistent with their capacity for directed neurite outgrowth (Figure S5c, Supporting Information). The Semaphorin, Netrin, Slit, and Ephrin families are four major axon guidance cue families in the nervous system.^[^ ^41^, ^42^ ^]^ Heatmap analysis confirmed the expression of key members and receptors across these four families in hONS (Figure S5d, Supporting Information). Among these cues, Semaphorins are a highly conserved superfamily of transmembrane/signaled proteins with well‐documented roles in SGN development, including axon branch refinement,^[^ ^43^ ^]^ projection patterning,^[^ ^44^, ^45^ ^]^ and membrane excitability.^[^ ^46^ ^]^ *SEMA6D*, a class 6 Semaphorin, plays critical roles in neurobiological processes such as axon guidance, neural circuit formation, and synaptic plasticity. We confirmed SEMA6D expression in neurons of Div50 hONS via immunostaining (Figure 6h). To test its functional role, we performed siRNA‐mediated *SEMA6D* knockdown in Div50 hONS prior to encapsulation in Matrigel for neurite extension assays. Knockdown of *SEMA6* resulted in a dramatic reduction in neurite outgrowth from otic spheroid neurons (Figure 6i), suggesting that SEMA6D contributes to regulating directed neurite projection in hONS.

### 2.6 hONS Discriminate Short‐Term Ototoxic Insults Through Graded Cellular and Calcium Response

**Figure 7:**
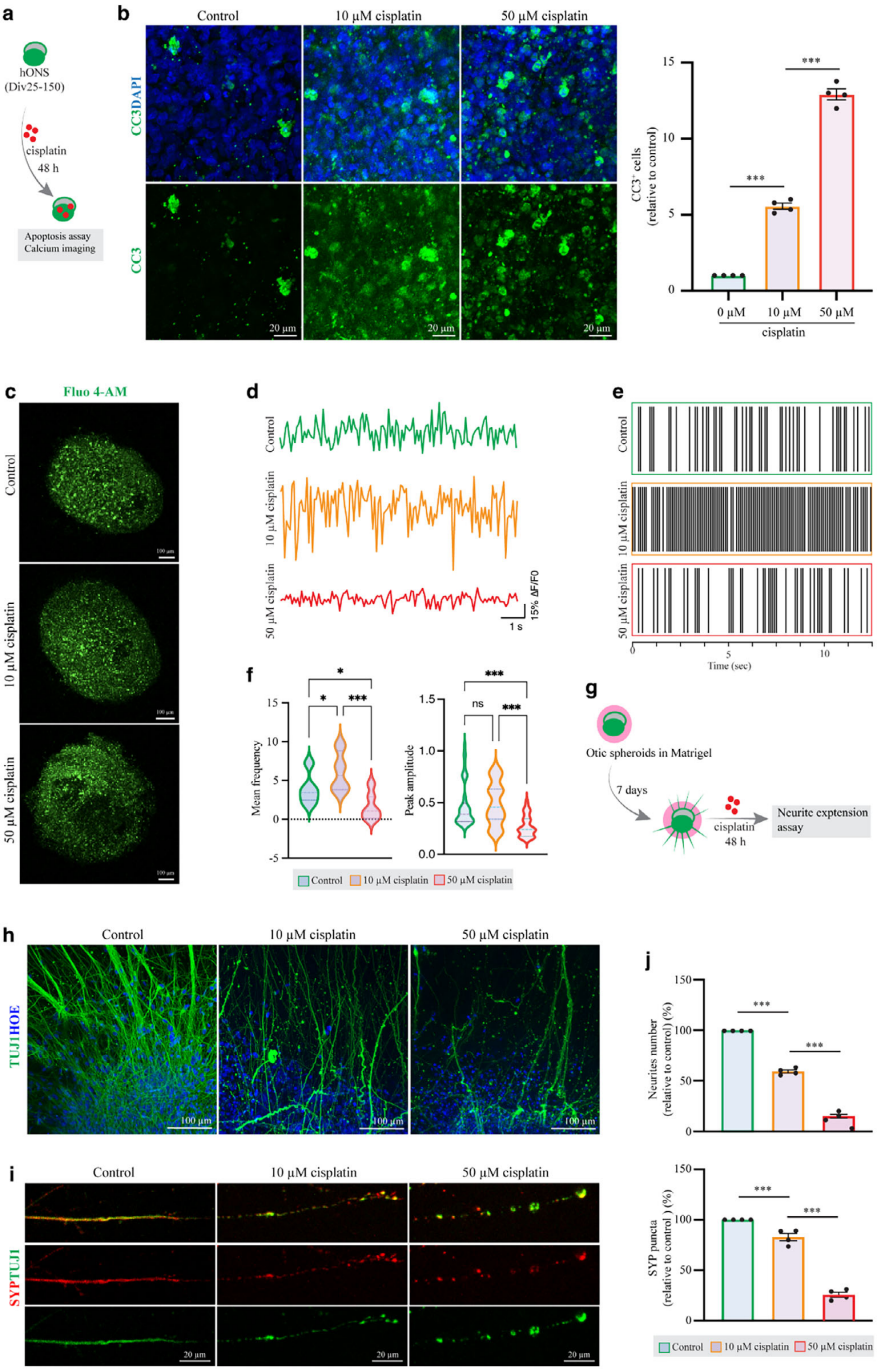
hONS discriminate short‐term ototoxic insults through graded cellular and calcium responses. a) Schematic diagram of short‐term ototoxic insults to hONS. b) Representative immunostaining and quantification of apoptosis (cleaved caspase‐3, CC3) in the aggregates treated with different dosages of cisplatin for 48 h. One‐way ANOVA followed by Tukey's test. n = 4 spheroids for each group. c) Representative calcium imaging of otic spheroids treated by different dosages of cisplatin. d‐f) Representative calcium traces, raster plots, and quantification of Fluo 4‐AM‐loaded otic spheroids in different groups. *n* = 3 to 5 spheroids for each group. g) Experimental design of neurite outgrowth assay. h–j) Representative immunostaining and quantifications of extended neurites (TUJ1^+^) near the core of spheroids and synaptic protein (SYP^+^TUJ1^+^) at the neurite tips in different groups. One‐way ANOVA followed by Tukey's test. n = 4 spheroids for each group. Values are presented as mean ± SEM. ***p < 0.001.

We investigated the response of hONS to cisplatin, a chemotherapeutic drug known for its ototoxicity and direct damage effects on SGNs. Initial experiments were conducted on native murine SGNs to establish a baseline sensitivity. Primary SGNs from postnatal day 3 mice were treated with 50 µm cisplatin, a concentration commonly used in in vitro SGN models,^[^ ^11^, ^12^ ^]^ or 10 µm cisplatin, an ultralow dose rarely applied in such studies, for 48 h. The results indicated that 10 µm cisplatin did not induce significant cell loss, apoptosis, or neurite retraction in murine SGNs. In contrast, 50 µm cisplatin markedly reduced SGN survival, impaired neurite outgrowth, and increased apoptosis, confirming that 10 µm represents a low, subtoxic dose in native murine SGN cultures (Figure S6a–c, Supporting Information). Subsequently, hONS generated from fresh and cryopreserved PPE cells were exposed to 10 or 50 µm cisplatin, respectively (**Figure** **7**a). As shown in Figures 7b and S7a (Supporting Information), both doses induced apoptosis in these cells. The number of apoptotic cells, labeled by cleaved Caspase3 (CC3^+^), significantly increased in a dose‐dependent manner in the cisplatin‐treated groups compared to controls (Figures 7b and S7a, Supporting Information). Calcium imaging revealed a marked reduction in the calcium‐labeled regions in spheroids treated with 50 µm cisplatin, along with significant decreases in mean frequency and peak amplitude of detected calcium traces, indicating severe damage to otic neurons in these spheroids (Figure 7c–f). In contrast, in the low‐dose (10 µm) treatment group, calcium imaging showed a higher mean frequency than in the control group without affecting the peak amplitude, suggesting that neurons might be in a reactive activation state (Figure 7c–f). Additionally, in a rescue assay, 1 mm sodium thiosulfate was co‐administered with 10 µm‐cisplatin for 48 h, and results demonstrated that sodium thiosulfate reversed the damage induced by 10 µm cisplatin to hONS, as evidenced by decreased CC3^+^ cells and increased neurite extension compared to the control (Figure S7b, Supporting Information). The sensitivity to 10 µm cisplatin in hONS of the cryopreserved‐group was validated using the H1 hESC line, which demonstrated increased CC3 ratio, and reduced neurite extension compared to the control, in consistent with those using H9 line (Figure S7c, Supporting Information). Furthermore, we evaluated the drug reactivity using neurite‐sprouted otic neuronal spheroids embedded in Matrigel to simulate the authentic responses of in vivo SGNs to ototoxic chemicals (Figure 7g). Cisplatin significantly reduced both the number of sprouted neurites and SYP puncta compared to the control group, even at the low dose of 10 µm (Figure 7h–j). We further extended our analysis to another ototoxin, the aminoglycoside antibiotic neomycin (1 mm), which induced significant apoptosis and neurite reduction in hONS but not in murine SGNs (Figures S6a–c and S7b, Supporting Information), further supporting the enhanced sensitivity of human otic spheroids.

**Figure 8:**
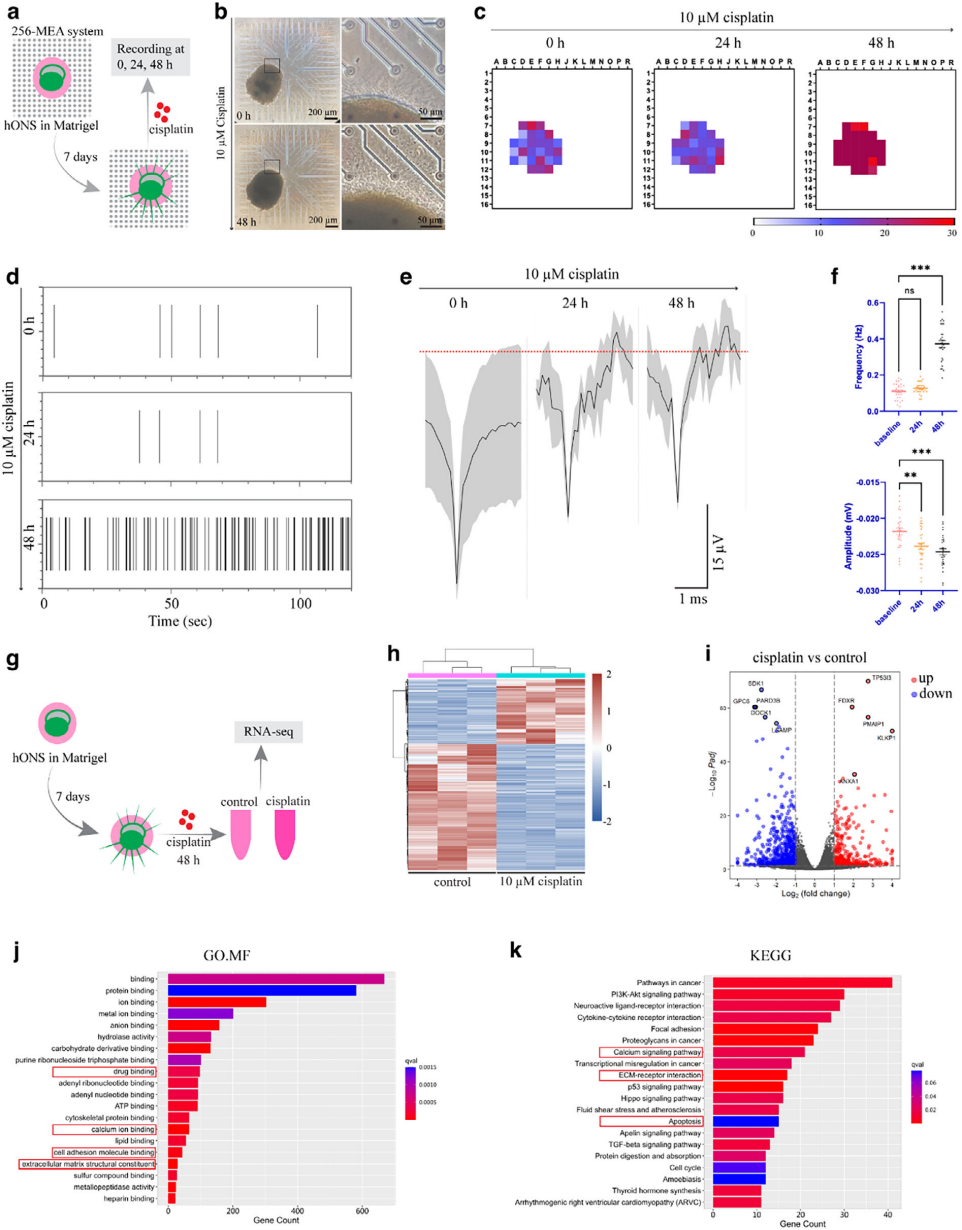
The electrophysiological and transcriptomic profiles of hONS are altered following short‐term exposure to low‐dose cisplatin. a) 256 MEA system for electrophysiological assay. b–e) Phase images (b), frequency heatmaps (c), raster plots (d), and stacked wave forms (e) of 10 µm cisplatin‐treated otic spheroid loaded on a 256‐MEA system. f) Quantification of spike frequency and amplitude. One‐way ANOVA followed by Tukey's test, n = 29 (Baseline), 29 (24 h), 28 (48 h), active electrodes for frequency quantification. n = 28 (Baseline), 29 (24 h), 27 (48 h) active electrodes for amplitude quantification. g) Experimental design for bulk RNA‐seq. h–k) Clustering heatmap (h), volcano plot (i), GO (j), and KEGG (k) assays of otic spheroids treated with or without 10 µm cisplatin for 48 h. Values are presented as mean ± SEM. ^**^ *P *< 0.01. ^***^ *P *< 0.001. ns, no significance.

The above findings suggest that even short‐term exposure to a low dose of cisplatin may cause substantial damage to otic neurons derived from hONS. To further evaluate functional deficits, we employed the MEA system on neurite‐sprouted spheroids following short‐term exposure to 10 µm cisplatin (**Figures** **8**a,b and S8a, Supporting Information). Spike frequency remained unchanged at the 24 h timepoint following cisplatin treatment but significantly increased after 48 h of treatment (Figure 8c,d,f and Figure S8b, Supporting Information). The spike amplitude assay proved greater sensitivity, showing a significant decrease at the 24 h timepoint and further deterioration at the 48 h timepoint post‐treatment (Figure 8e,f and Figure S8c, Supporting Information), indicating progressive neuronal damage in the spheroids. Glutamate stimulation slightly reduced spike frequency, confirming the impairment of electrophysiological function (Figure S8d,e, Supporting Information). The isolated phenotype of increased spike frequency and decreased amplitude observed in neuronal spheroids exposed to low‐dose ototoxic insults may represent an early characteristic of neuronal damage. Bulk RNA‐seq analysis of spheroids treated with 10 µm cisplatin for 48 h identified 1044 differentially expressed genes (DEGs) associated with mitochondrial function, apoptosis, and glial/neuronal processes (Figure 8h,i and Figure S8f, Supporting Information). Glial marker genes, including *SOX2*, *GFAP*, and *PLP1* were downregulated, while the apoptosis‐related gene *CASP3* was upregulated (Figure S8g, Supporting Information). Most neuronal genes were upregulated, whereas glial genes were downregulated (Figure S8h,i, Supporting Information). GO and KEGG analyses highlighted enrichment in drug binding, calcium signaling, and extracellular matrix organization (Figure 8j,k).

Given that our hONS is a complex composed of otic neurons and glial cells following maturation, we also explored the role of glia in neuronal survival in response to ototoxicity. Specifically, we treated otic spheroids with 10 µm cisplatin alone or in combination with 10 µm minocycline, a pharmacological inhibitor of the glia pathway. Results revealed that compared to the cisplatin‐only group, the minocycline + cisplatin group showed significantly more CC3⁺ cells and reduced neurite extension (Figure S7b, Supporting Information), indicating a protective role of glia cells in our otic spheroids in mitigating ototoxic damage.

In summary, these findings demonstrate that even short‐term, low‐dose cisplatin exposure induces substantial damage in hONS, reflecting heightened sensitivity compared to murine models. The transcriptional and functional changes provide insights into early ototoxic mechanisms and support the utility of this model for preclinical ototoxicity screening and protective strategy validation.

### 2.7 A Long‐Term Exposure of Trace Ototoxic Insult Alters Glutamatergic Neural Functionality in hONS

**Figure 9:**
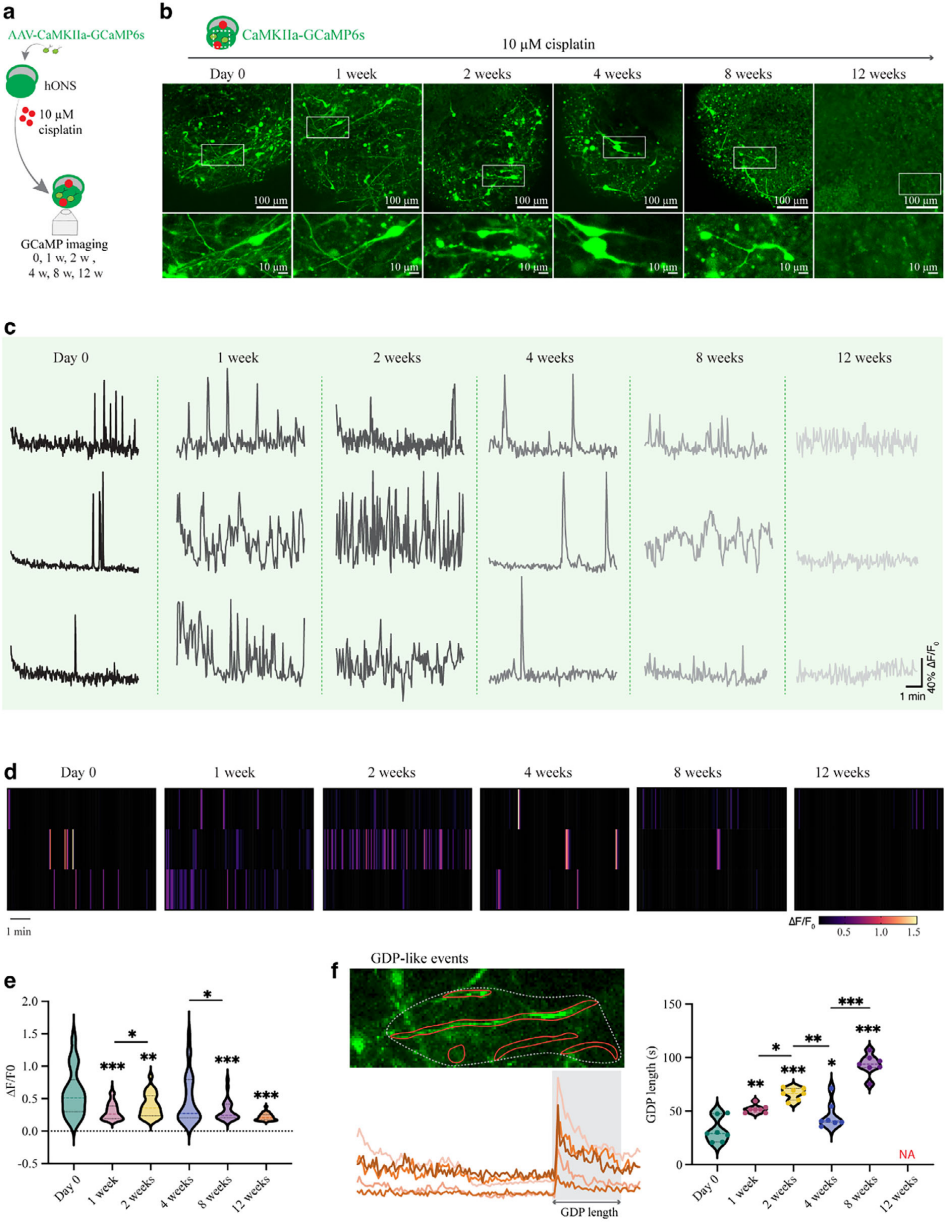
A long‐term exposure of trace ototoxic insult alters glutamatergic neural functionality in hONS. a) Experimental design of long‐term ototoxic insult. b) Representative images of virally encoded hONS treated with 10 µm cisplatin for different times. c,d) Representative calcium traces (c) and frequency heatmaps (d) of regions of interest from three randomly selected ROIs in otic spheroids treated with 10 µm cisplatin for different days. e) Quantification of ΔF/F0 ratios of calcium traces detected from otic spheroids in different groups. One‐way ANOVA followed by Tukey's test*. n* = 18‐97 ROIs. f) Quantification of GDP length between different groups. One‐way ANOVA followed by Tukey's test. *n* = 7. Values are presented as mean ± SEM. ^*^ *P *< 0.05, ^**^ *P *< 0.01, ^***^ *P *< 0.001.

Clinically, most drug‐related adverse reactions stem from cumulative damage following prolonged exposure to low‐to‐moderate doses. To explore the long‐term effects of cisplatin on the neural function of otic spheroids, we continuously monitored the calcium activity of otic neurons (transfected with AAV‐CaMKII‐GCaMP6s) over 12 weeks of 10 µm cisplatin treatment (**Figure** **9**a). After 2 weeks, glutamatergic neurons showed truncated neurites and condensed soma, which progressively worsened until no neurons remained (Figure 9b and Figure S9a,b, Supporting Information). The glutamatergic neuron population declined and stabilized by day 28 (Figure 9b). Calcium transients remained relatively constant during the first 4 weeks, underwent marked changes at week 8, and were barely detectable by week 12 (Figure 9c,d). The average calcium ratio initially decreased at week 1, returned to normal at week 4, and then declined continuously thereafter, indicating that otic spheroids could compensate for low‐dose cisplatin insults for ≈4 weeks (Figure 9e). The duration of GDP‐like events increased during the first 2 weeks, regressed noticeably in the following 2 weeks, then rebounded beyond baseline levels, and was nearly undetectable by week 12 (Figure 9f).

To evaluate the functional state of the spheroids, we calculated the mean ∆F/F0 ratio across the entire hONS and compared the general calcium activity over time. No significant differences were observed during the first 4 weeks, but a marked decrease occurred by week 8 (Figure S9c, Supporting Information). The coefficient of variation (CV) remained below 10% for the first 4 weeks, then sharply increased to exceed 17.1% at week 8, suggesting progressive neuronal instability and reduced activity following cisplatin treatment (Figure S9d, Supporting Information). These findings demonstrate that 10 µm cisplatin is neurotoxic to the induced otic neurons, with irreversible damage appearing after at least 4 weeks of exposure.

## 3 Discussion

In recent decades, stem cell‐based technologies have advanced significantly in disease modeling, drug screening, and cell replacement therapy for neurodegenerative disorders.^[^ ^47^, ^48^, ^49^, ^50^, ^51^ ^]^ In the auditory field, cochlear neuronal spheroids or organoids derived from hPSCs have been effectively utilized to recapitulate inner ear development, model hearing loss, and identify ototoxic and otoprotective compounds.^[^ ^27^, ^28^, ^52^, ^53^ ^]^ However, their widespread application has been limited by challenges of reproducibility, heterogeneity, incomplete maturation, and operational complexity. Conventional approaches that differentiate otic organoids directly from hPSCs are not only labor‐intensive and time‐consuming but also susceptible to batch‐to‐batch variability due to the inherent sensitivity of pluripotent cells to environmental fluctuations. To address these limitations, we developed a novel strategy based on the cryopreservation of an otic precursor population, specifically the PPE cells. This approach enables the reproducible and on‐demand generation of hONS with markedly reduced differentiation time and enhanced inter‐batch consistency. Transcriptomic and functional analyses confirmed that these spheroids not only exhibit classical markers of otic neurogenesis with no significant differences in marker expression between cryopreserved and fresh groups, but also exhibit electrophysiological maturity, characterized by stably negative resting membrane potentials, TTX‐sensitive action potentials, synchronized network activity, and glutamatergic responses with consistent spike amplitude and frequency between cryopreserved and fresh cohorts. Notably, bulk RNA‐seq revealed a high degree of similarity with human fetal cochlear datasets and distinct transcriptional profiles relative to non‐PPE‐derived hONS, further validating the physiological relevance of the in vitro‐derived SGN‐like neurons. The use of cryopreserved PPE cells thus decouples the differentiation process from continuous hPSC culture, standardizes the source material, and offers a promising platform for high‐throughput screening applications.

The electrophysiological properties of SGNs enable their high sensitivity and precise encoding of auditory information, modulating the fine perception within the auditory system.^[^ ^7^, ^54^, ^55^, ^56^ ^]^ Our data illustrate that cryopreserved hPSC‐derived otic precursors can be differentiated into hONS displaying key functional attributes of mature SGNs, including stable calcium transients, glutamatergic excitability, and robust neurite outgrowth. The presence of GDP‐like events further supports the emergence of intrinsically active and synaptically connected networks, consistent with previous in vivo and in vitro observations during auditory circuit maturation.^[^ ^29^, ^57^, ^58^, ^59^ ^]^ MEA recordings provided additional evidence of functional maturation, demonstrating dose‐responsive changes in firing rate following glutamate application and inhibition with CNQX. These findings confirm the presence of functional glutamatergic synapses mediated via AMPA receptors within the spheroids, which is consistent with the known excitatory effects of glutamate in mature neuronal networks.^[^ ^60^, ^61^ ^]^ The combination of 3D otic spheroids with high‐resolution MEA systems offers a powerful and non‐invasive platform^[^ ^62^, ^63^ ^]^ for assessing network‐level responses to ototoxic agents or potential therapeutics under conditions that support long‐term culture and repeated measurements.

Furthermore, we demonstrated the capacity of human otic neurons to establish afferent and efferent projections within complex micro‐environmental contexts. In tripartite coculture systems involving murine cochlear explants and hCO, otic neurites exhibited targeted outgrowth toward both HCs and cortical tissues, forming synaptic specializations positive for SYP and PSD95. Optogenetic stimulation confirmed functional synaptic transmission between otic spheroids and both peripheral and central targets. While previous studies have shown PSC‐derived SGN‐like cells could innervate HCs or HC‐like cells,^[^ ^28^, ^64^ ^]^ our work advances this by demonstrating functional bidirectional connections, mimicking the native auditory circuit. We also identified potential axon guidance cues, SEMA6D, whose knockdown impaired neurite extension. This aligns with known roles of SEMA6D in regulating axon pathfinding and target selection in retinal ganglion cell axons through interactions with Plexin‐A1 and Nr‐CAM complexes.^[^ ^65^, ^66^ ^]^ These findings suggest conserved mechanisms may govern both visual and auditory circuit formation, warranting further investigation into SEMA6D‐mediated signaling in cochlear development.

Stem cell‐derived organoids are increasingly recognized as transformative platforms for toxicology studies and disease modeling.^[^ ^67^, ^68^ ^]^ Previous studies have utilized otic organoids derived from hiPSCs or murine sensory epithelium to assess the ototoxic effects of compounds such as cisplatin, ouabain, and aminoglycoside antibiotics such as sisomicin and gentamicin.^[^ ^27^, ^69^, ^70^ ^]^ Notably, clinical cisplatin‐induced ototoxicity typically results from cumulative, low‐dose exposure during chemotherapy regimens,^[^ ^12^, ^71^ ^]^ yet most preclinical studies within otic organoid models have relied on high, acute concentrations to induce damage.^[^ ^27^, ^70^ ^]^ For instance, Kurihara et al. reported substantial neuronal damage at 100 and 200 µm cisplatin in hiPSC‐derived otic organoids,^[^ ^27^ ^]^ and Qin et al. found 75 µm cisplatin to be ototoxic to HCs in cochlear organoids.^[^ ^70^ ^]^ In contrast, our hONS exhibit unprecedented sensitivity to low‐dose cisplatin (10 µm), a concentration that proved subtoxic to primary murine SGNs. Following short‐term (48 h) exposure, 10 µm cisplatin induced significant alterations in the spheroids, including increased firing frequency accompanied by reduced sprouted neurites and synaptophysin puncta, and initiated apoptosis. This distinct electrophysiological and morphological profile suggests an early “reactive” state in which neurons remain viable yet undergo compensatory changes in response to cisplatin‐induced stress. In contrast, 50 µm cisplatin resulted in widespread apoptotic cell death. Transcriptomic analysis further revealed dysregulation of genes associated with mitochondrial function, apoptotic signaling, and glial support, offering mechanistic insights into the early stages of cisplatin‐induced injury.^[^ ^72^, ^73^ ^]^ Additionally, long‐term exposure (12 weeks) to 10 µm cisplatin revealed cumulative toxic effects: although calcium signaling remained stable during the first four weeks, progressive neurite degeneration, loss of calcium transients, and eventual neuronal death were observed thereafter, indicating that sustained low‐dose exposure may lead to irreversible auditory deficits. Importantly, we found that the presence of glial cells within the spheroids contributed to neuroprotective mechanisms, highlighting the importance of preserving neuron‐glia interactions in vitro. The platform also demonstrated appropriate responsiveness to other ototoxins such as neomycin and validated the protective effects of sodium thiosulfate, a clinically approved otoprotectant.^[^ ^74^ ^]^ Together, the capability of our hONS to model early, reversible neuronal dysfunction addresses a significant gap in current ototoxicity models. Moreover, the low‐dose, long‐term cisplatin response closely resembles clinical exposure scenarios, underscoring the unique advantage of this hONS‐based model in recapitulating physiologically and clinically relevant ototoxic pathways beyond those traditional animal‐based approaches.

In summary, this study describes a standardized, reproducible system for generating functional hONS from cryopreserved hPSC‐derived PPE cells. These spheroids recapitulate key features of native SGNs, including otic marker expression, glutamatergic electrophysiology, bidirectional functional connectivity, and sensitive ototoxic responses, while also offering the practical advantages of batch‐to‐batch consistency and off‐the‐shelf availability. This model facilitates modeling of SGN‐related hearing disorders and provides a robust platform for preclinical ototoxicity screening. However, challenges remain in achieving mature adult SGN‐like function in vivo, and further evaluation of a broader range of ototoxic and otoprotective drugs is needed to fully assess the accuracy and specificity of this platform.

## 4 Experimental Section

### hPSCs and Culture

All the human stem cell work in this study was performed under the approval of the Ethics Committee of the Institute of Zoology, the Chinese Academy of Sciences (2021, No.008). The human embryonic stem cell (hESC) lines H9 (passages 22 to 40) and H1 (passages 61 to 75) were utilized for the generation of otic neuronal spheroids and cortical organoids. H9 hESCs were cultured in Essential 8 (E8) Basal Medium (Gibco) or StemFit Basic04 medium (AJINOMOTO) on 6‐well plates pre‐coated with 1% hESC‐qualified Matrigel matrix (Corning). Cells were passaged using 0.5 mm EDTA (Gibco) every 5–7 days. Before differentiation, the pluripotency of hPSCs were confirmed through immunostaining for the pluripotent markers OCT4, SOX2, NANOG, and SSEA4.

### Mice and Ethical Statement

Wildtype postnatal day 3 (P3) C57BL/6J mice used in this study for cochlear and cerebral cultures were purchased from SPF Biotechnology (Beijing, China) and the Animal Center of Shandong University (Jinan, China). All the animal‐involved experiments in the present study were performed under the approval of the Animal Ethics Committee of the Institute of Zoology, Chinese Academy of Sciences, and the Animal Care of Shandong University, China (NO. ECAESDUSM 20 123 011), complying to the National Institutes of Health Guide for the Care and Use of Laboratory Animals.

### Generation and Cryopreservation of PPE Cells

The generation of PPE‐like cells using hPSCs was based on the previously described method with minor modifications.^[^ ^25^ ^]^ Briefly, hPSCs were dissociated using StemPro Accutase (Gibco) and resuspended in E8 medium supplemented with 10 µm Y27632 (Selleck) and 100 µg mL^−1^ Normocin (InvivoGen). Cell suspension was then seeded onto 6‐well plates pre‐coated with 1% Growth Factor Reduced (GFR) Matrigel (Corning) at a density of 10000 cells cm^−^^2^, marking differentiation day 0 (D0). The monolayer hESCs underwent stepwise PPE induction in medium supplemented with corresponding small molecular reagents and growth factors (Figure S1a, Supporting Information). Growth factors were solely from Peprotech except BMP4 (Peprotech or ARCO). Small molecules were from Sigma, Selleck or MCE. On D11, cells were cryopreserved as follows: cells were dissociated into single cells using Accutase and cryopreserved in icy NB‐CDM medium, DMEM‐F12 medium with 1% N2, 2% B27, 0.1 mm β‐Mercaptoethanol (β‐ME), and 50 µg mL^−1^ Normocin, supplemented with 10% DMSO in liquid nitrogen.

### Generation of hONS using Cryopreserved PPE Cells

For 3D differentiation, the cryopreserved PPE cells were thawed and resuspended in NB‐CDM medium supplemented with 3 µm GSK3β inhibitor CHIR99021 (Selleck), 10 ng mL^−1^ bFGF, 50 ng mL^−1^ IGF‐1 (Peprotech), and 10 µm Y27632. These cells were then seeded into low‐adhesive V‐bottom 96‐well plates at a density of 10000 cells well^−1^, marking the first day of 3D differentiation as day 0 in vitro (Div0). The aggregates underwent stepwise incubation in media supplemented with specific small molecular reagents and growth factors, as indicated in Figure S1a (Supporting Information). The NIM medium was 48% DMEM‐F12 medium, 48% Neurobasal medium, 1% N2, 2% B27, and 50 µg mL^−1^ Normocin. The otic differentiation media used after Div15 were applied in the following assays.

### Assessment of Organoid Formation Rate

H9 and H1 hESCs were used to generate PPE cells for cryopreservation. For each cell line (H9 and H1), two independent batches were cryopreserved. From each batch, two randomly selected vials of cryopreserved PPE cells were thawed, resulting in 8 vials total, and seeded into low‐adhesion V‐shaped 96‐well plates (10, 000 cells well^−1^) to induce self‐aggregation. After four days of culture, the organoid formation success rate was calculated as the percentage of wells with successfully formed spheroids relative to the total number of wells (spheroid‐formed wells/total wells × 100%).

### Generation of Human Cortical Organoids

Human cortical organoids (hCOs) were differentiated as previously described with minor modifications.^[^ ^75^ ^]^ Briefly, on day 0, H9 hESC colonies were dissociated by Accutase and 5000 cells per well were plated into low‐adhesive V‐bottom 96‐well plates. The hCO differentiation media after day 18, 48% DMEM‐F12 medium, 48% Neurobasal medium, 0.5% N2, 2% B27, 0.5% NEAA, 1% GlutaMAX, 7 µg mL^−1^ insulin, 50 µm β‐ME, 20 ng mL^−1^ BDNF, 200 µm L‐ascorbic acid, and 50 µg mL^−1^ Normocin, were applied in the following assays.

### Morphological Assay of Otic Neurons

hONS (Div25‐40) were dissociated into single cells for morphological assay and peripheral projection test. Briefly, spheroids were cut into small pieces (thickness less than 100 µm) with a disposable ophthalmic scalpel and incubated in Accutase for 30 min at 37 °C. An equal volume of 20% FBS in DMEM‐F12 medium was used to terminate the enzymatic digestion. Then gentle suction and pipette was performed to minimize the resident pieces in the solution before filtering through a 70‐µm cell strainer. After two washes with DMEM‐F12, dissociated cells were resuspended in otic differentiation medium used after Div15, with 10 µm Y27632, and seeded on cover‐slips pre‐coated with 1% Matrigel (25000‐ 40000 cells cm^−2^) for 48 h. The morphology of otic neurons were assayed by immunostaining using TUJ1antibody.

### Viral Labeling

The hONS or cortical organoids were transferred to a low‐adhesive 24‐well plate containing 500 µL corresponding culture medium with virus (5.5 × 10^10^ GC mL^−1^), and each well with no more than 2 samples. After two days, spheroids or organoids were transferred into fresh medium for further applications. Adenoviruses AAV‐CaMKIIa‐GCaMP6s and AAV‐CaMKIIa‐ChR2‐mCherry were generated in Genomeditech (Shanghai) Co., Ltd (Shanghai, China). Viral labeled spheroids alone or in coculture systems were confirmed using a Zeiss 880 confocal microscope after 10 to 14 days of infection.

### Assessment of the peripheral Projection of SGNs

mBM explants were isolated and cultured as previously described.^[^ ^76^ ^]^ Then mBM‐loaded slides were plated into 24‐well plates. Dissociated otic neurons from otic spheroids (Div25‐40) resuspended in otic differentiation medium (after Div15) containing 10 µm Y27632 and 2% Matrigel were evenly seeded onto the mBM‐loaded slides, at a density of 50000 cells cm^−2^. An equal volume of fresh medium was slowly added to the previous 200 µL medium 24 h after seeding. Samples were collected after 7 days of coculture.

### Assessment of Central Projection of SGNs

The otic spheroid (Div25‐40) and an hCO (day 45‐60), which were labeled with AAV‐CaMKIIa‐GCaMP6s two days before, were transferred to a 1.5 mL tube, washed with DMEM‐F12 medium, and suspended in pure Matrigel. Then a 30‐µL droplet containing them was distributed to a 24‐well confocal plate. The culture medium used was a 1:1 mixture of their corresponding medium. Confocal imaging of GCaMP calcium signals was performed 21 and 42 days after assembly.

### Assessment of Bidirectional Projection of SGNs

A tripartite coculture system was constructed. Briefly, hCO, hONS, and middle turn of P3 mBM were transferred to a 1.5 mL tube, washed with DMEM‐F12 medium, and supplemented in pure Matrigel. Then, a 50‐µL droplet containing three parts was distributed to the surface of culture substrates, MEA, coverslip, or a 24‐well confocal plate, for different experimental purposes. The culture medium used was a 1:1:1 mixture of their corresponding medium.

### Time‐Lapse Live Imaging

After confirmation of GGaMP6s expression in hONS via confocal imaging, a tripartite coculture system expressing AAV‐CaMKIIa‐GCaMP6s in hONS was assembled and placed on the surface of a 24‐well confocal plate in culture medium. After 24 h incubation at 37 °C and 5% CO2, time‐lapse imaging was performed at 15–20 min interval for a continuous 60 h track on a Leica SP8 confocal microscope.

### RNA Extraction and Real‐Time Polymerase Chain Reaction

Total RNA was extracted from the cells using a TRIzol reagent according to the manufacturer's instructions. cDNA was synthesized by reverse transcription using the Revert Aid First Strand cDNA Synthesis Kit (Thermo Scientific, USA), following the manufacturer's protocol. Quantitative real‐time PCR was performed using SYBR Premix Ex Taq (TaKaRa Bio). Actin was used as the housekeeping gene. All data were analyzed using the Eppendorf Realplex 2. The PCR primers used are listed in Table S2 (Supporting Information).

### Immunostaining

For monolayer cells, samples were fixed using warm 4% paraformaldehyde for 20 min, followed by two rinses with PBS for 30 min. For spheroids or organoids, samples were fixed in 4% paraformaldehyde at 4 °C overnight and immersed in PBS at 4 °C overnight. The samples were then incubated in a blocking solution composed of 1% bovine serum albumin (BSA, Sigma), 5% heat‐inactivated donkey serum (NQBB), and 0.1% Triton X‐100 (Sigma) in PBS at room temperature. This was followed by an overnight incubation at 4 °C with primary antibodies, as detailed in Table S1 (Supporting Information), diluted in the blocking solution. Subsequent to three PBS washes containing 0.1% Triton X‐100 at room temperature for 1 h, the samples were incubated with Alexa Fluor conjugated secondary antibodies (Invitrogen, 1:1000) and Hoechst 33342 (Sigma, 2 µg mL^−1^) or DAPI (Sigma, 1 µg mL^−1^), both diluted in blocking solution, for 1 h at room temperature. Samples were imaged using a ZEISS LSM 880 inverted microscope.

### Neurite Extension Assay

hONS were transferred to a 1.5 mL tube, washed with DMEM‐F12 medium, and suspended in pure Matrigel. A 20‐µL droplet containing a single spheroid was then evenly distributed to the surface of a glass coverslip in a 24‐well plate. After gelation at 37 °C, the otic differentiation medium used after Div15 was added. Cultures were sampled or used for other assays after 7 days of cultivation. Medium was changed every two days.

### Bulk RNA Sequencing

Total RNA was isolated from the collected samples using the TriZol reagent (Invitrogen). The quality, including purity, concentration, and RNA integrity number (RIN) of the isolated RNA, was assessed using the NanoPhotometer (IMPLEN) and the Agilent 2100 RNA Nano 6000 assay kit (Agilent Technologies). RNA‐seq libraries for each sample were prepared using the VAHTS Universal V6 RNA‐seq Library Prep Kit (NR604‐01/02), following the manufacturer's protocol, and sequenced on the Novaseq 6000 S4 platform with the NovaSeq 6000 S4 Reagent kit V1.5. Gene expression levels were quantified as fragments per kilobase per million (FPKM), with a threshold of FPKM > 1 indicating positive expression in the samples. Principal Component Analysis (PCA), correlation heat map, dot analysis, identification of differentially expressed genes (DEGs), and Gene Ontology (GO) analysis were conducted to evaluate the differences and similarities among the individual samples.

### Calcium Imaging

For calcium activity analysis, whole samples were loaded with 1 µm Fluo‐4 AM (Invitrogen) at 37 °C for 1 h. For both Fluo‐4 AM‐labeled spheroids and GCaMP‐encoded samples, calcium imaging was conducted on a ZEISS LSM 880 inverted confocal microscope equipped with ZEN 2010 software and 10x and 20x dry objectives, at an excitation wavelength of 488 nm. Time‐lapse images were captured at 0.2–3 Hz. Activation of the glutamatergic neurons was obtained by 10 µm glutamate (Sigma). Glutamate receptors were blocked by 20 µm CNQX (Selleck) or 1 µm MK‐801 (Selleck). Fluorescent image series were analyzed using the MeanROI component in ZEN 2010. Relative fluorescence intensity change (ΔF/F0) was calculated to indicate calcium responses in spheroids under different conditions, and ΔF/F0 values≥0.15 were considered to be calcium‐responsive.

### Microelectrode Assay (MEA) Recording

Spontaneous extracellular field potentials in otic neuronal spheroids were recorded using a 256‐channel MEA system (MEA2100 system, multichannel systems, Germany) at a sampling rate of 10 kHz and a controlled temperature of 37 °C (TC02, Multi Channel Systems). The spheroids were maintained in modulating medium in a single MEA well equipped with 256 electrodes (MCS, Reutlingen, Germany) for 7 days. Recordings of five minutes each were obtained using Multi Channel Experimenter software (version 2.15.0). Spike detection was performed with Multi Channel Analyzer software (version 2.15.0), employing an adaptive threshold set at 4.2 times the standard deviation above the estimated noise level for each electrode. Electrodes registering a minimum of 3 spikes per minute were designated as active electrodes.

### Optogenetic Stimulation and MEA Recording

After confirmation of ChR2 expression in hONS via confocal imaging, a tripartite coculture system expressing AAV‐CaMKIIa‐ChR2‐mCherry in hONS was assembled and placed on the surface of a 256‐MEA well in culture medium. After 7 days of coculture, ChR2‐positive cells in hONS were stimulated with 473 nm light using a fiber optic (Shanghai Dreamlasers Technology), and electrical changes in response to optogenetic stimulation were recorded using a 256‐channel MEA system. The stimulation mode comprised 1500 frames, and one 473‐nm pulse of LED light (50 ms) was performed every 30 frames. Laser timing was controlled by a synchronization controller (BiolinkOptics). MEA assay was conducted for 2 min, 30 s before stimulation, 1 min stimulation, and 30 s post‐stimulation. Changes of firing patterns of the tripartite coculture system in response to optogenetic stimulation were detected using Multi Channel Analyzer software (version 2.15.0).

### Patch‐Clamp Electrophysiological Recordings

Electrical signals were recorded using an EPC10 (HEKA, Germany) patch‐clamp amplifier under the control of Patchmaster v.2 × 73.5 (HEKA, Germany) was used for data acquisition, and Fitmaster v.2 × 91 for data analysis. The pipettes were prepared with the micropipette puller (P‐97, Sutter Instrument) to yield a resistance of 5–7 MΩ. The neurons were perfused with a bath solution containing 126 mm NaCl, 2.5 mm CaCl2, 4.9 mm KCl, 1.2 mm KH2PO4, 2.4 mm MgSO4, 26 mm NaHCO3, and 10 mm glucose, at pH 7.4, which was adjusted using NaOH, and an osmolarity of 310 mOsm, unless otherwise stated. The recording pipette solution contained 130 mm K‐gluconate, 6 mm KCl, 10 mm HEPES, 1 mm EGTA, and 2.5 mm Na2‐ATP, at pH 7.2, which was adjusted using KOH, and an osmolarity of 300 mOsm. Action potentials (APs) were evoked using somatic current steps (duration: 500 ms) by −100 to +200 pA with increments of 10 pA. The recordings were sampled at 20 kHz, low‐pass filtered at 10 kHz, and digitally filtered at 2 kHz using Patchmaster. Recordings with an access resistance of > 20 MΩ were excluded from the analyses.

### Cell Viability Assay

Cell viability was assessed through live cell imaging, employing the MitoTracker Red kit (Invitrogen) according to the manufacturer's guidelines. Spheroids were incubated with 100 nm MitoTracker Red at 37 °C for 30 min, followed by two washes in fresh medium. Then spheroids were encapsuled in pure Matrigel and cultured in a 24‐well confocal plate. Confocal imaging was performed on the 1, 3, 5, and 7 days after droplet at an excitation wavelength of 594 nm using a ZEISS LSM 880 inverted microscope.

### Drug Treatment

The ototoxic drug cisplatin (Sigma), neomycin (Sigma), and otoprotectant sodium thiosulfate (Sigma) were dissolved in deionized water, respectively. Samples were exposed to 10, 50, or 100 µm cisplatin, 1, 8, or 16 mm neomycin, 1 mm sodium thiosulfate, or 10 µm minocycline (MCE), as described in the text. For the control group, deionized water, matched in volume to the largest volume of the corresponding drug solution. For long‐term evaluation, CaMKIIa‐GCaMP6s labeled hONS were treated by 10 µm cisplatin for up to 3 months. Confocal sampling was performed on 0, 1, 2, 4, 8, and 12 weeks.

### Statistical Analysis

Immunofluorescent images were quantitatively analyzed using ImageJ software (version 1.50i, Wayne Rasband, National Institutes of Health, USA). Unless specified otherwise, all samples were collected for at least three replicates. Data were presented as mean ± SEM. Statistical analysis was performed using Student's *t*‐test for comparisons between two groups and one‐way ANOVA followed by Tukey's multiple comparison test for multiple group comparisons. A *P*‐value of less than 0.05 was considered statistically significant.

## Conflict of Interest

The authors declare no conflict of interest.

## Author Contributions

G.S., Y.W. and M.W. contributed equally to this work. G.S., Y.W., H.W., W.L., and L.X. conceived and designed the project. G.S. constructed experimental flowcharts of MEA and calcium imaging. G.S., M.T., and X.Y.W. performed MEA and primary data analysis. X.Y.W. collected raw data of calcium imaging under the supervision of G.S. D.L. provided technical supports. Y.J., M.W., X.W., W.H., S.S., J.Q., L.C., W.A., L.K., A.S. made constructive suggestions. W.L. and Y.W. supervised the project. G.S. and W.L. wrote the manuscript.

## Supporting information



Supporting Information

Supplemental Data

Supplemental Movie 1

Supplemental Movie 2

## Data Availability

The data that support the findings of this study are available from the corresponding author upon reasonable request.
