# Supplementary material for: Cryopreserved Human Otic Neuronal Spheroids Self‐assemble for Functional Connectivity Analysis and Long‐term Ototoxicity Evaluation
Source: Adv Sci (Weinh). 2025 Nov 21;13(7):e05663. doi: 10.1002/advs.202505663 (PMC12866767; doi:10.1002/advs.202505663)
Supplement: Supplementary file 1 — Supporting Information [file ADVS-13-e05663-s004.docx]

Supporting Information

Cryopreserved Human Otic Neuronal Spheroids Self-assemble for Functional Connectivity Analysis and Long-term Ototoxicity Evaluation

Gaoying Sun, Yukai Wang, Man Wang, Xinyue Wang, Mingming Tang, Da Li, Jianhuan Qi, Xue Wang, Shujuan Sun, Lei Chen, Weibin An, Ligang Kong, Anqi Suo, Haibo Wang*, Wenwen Liu*, Lei Xu*

Figure S1


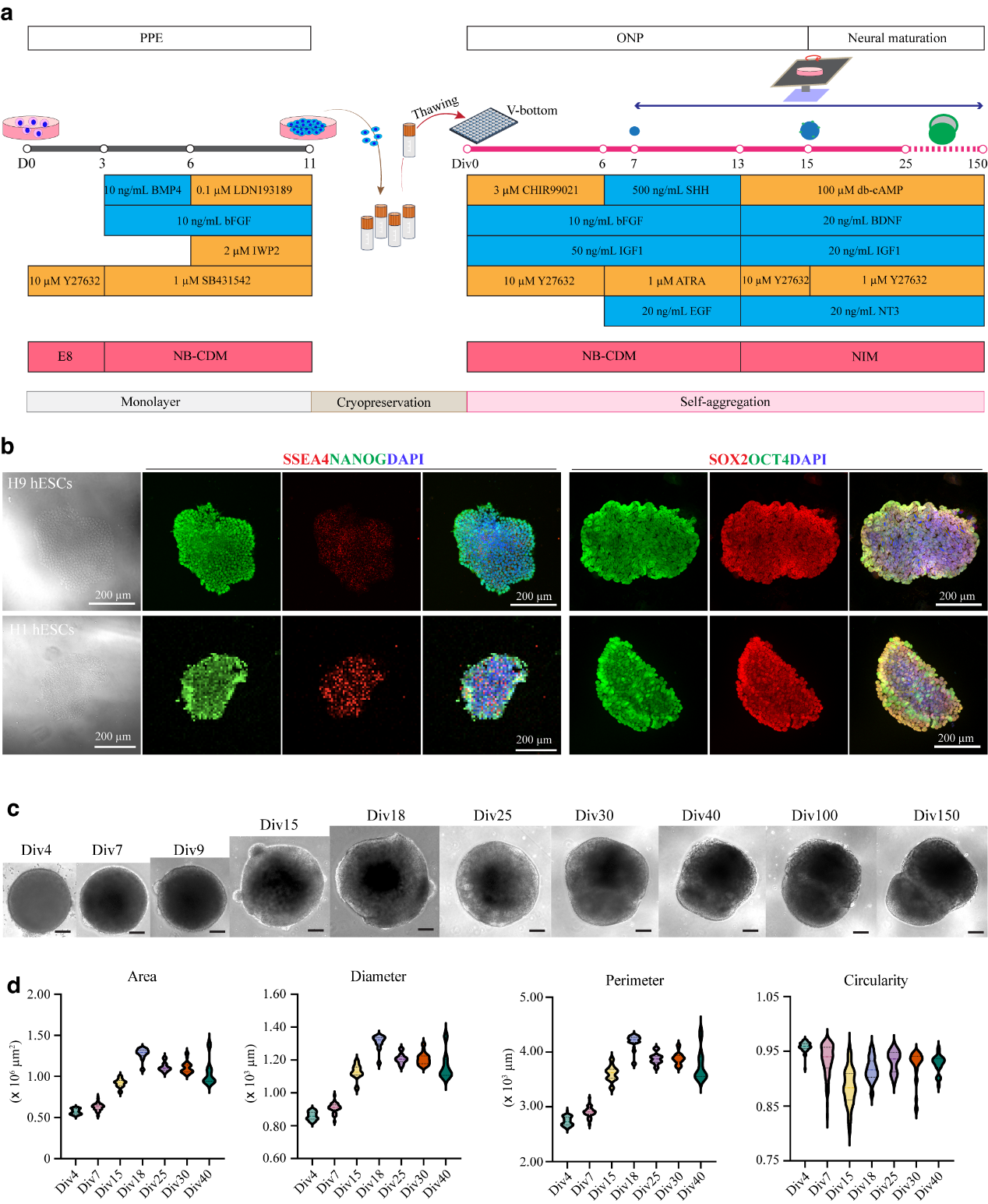


**Figure S1.** Generation of human otic neuronal spheroids (hONS). **a)** Schematic diagram and key treatments. Abbreviations: BMP4, bone morphogenetic protein 4; SHH, Sonic hedgehog; ATRA, all-trans retinoic acid; EGF, epidermal growth factor; BDNF, brain-derived neurotrophic factor; NT3, neurotrophin 3; IGF-1, insulin-like growth factor 1; FGF2, fibroblast growth factor 2. This protocol is adapted from previously reported studies.^[1-3]^ (**b)** Phase and immunostaining images of H9 and H1 hESC lines. Pluripotency markers, OCT4, SOX2, NANOG, and SSEA4. **c-d)** Phase images and quantification of hONS after aggregation. Bars, 200 µm. n=3~7 spheroids for each group. Data are presented as mean ± SEM.

Figure S2


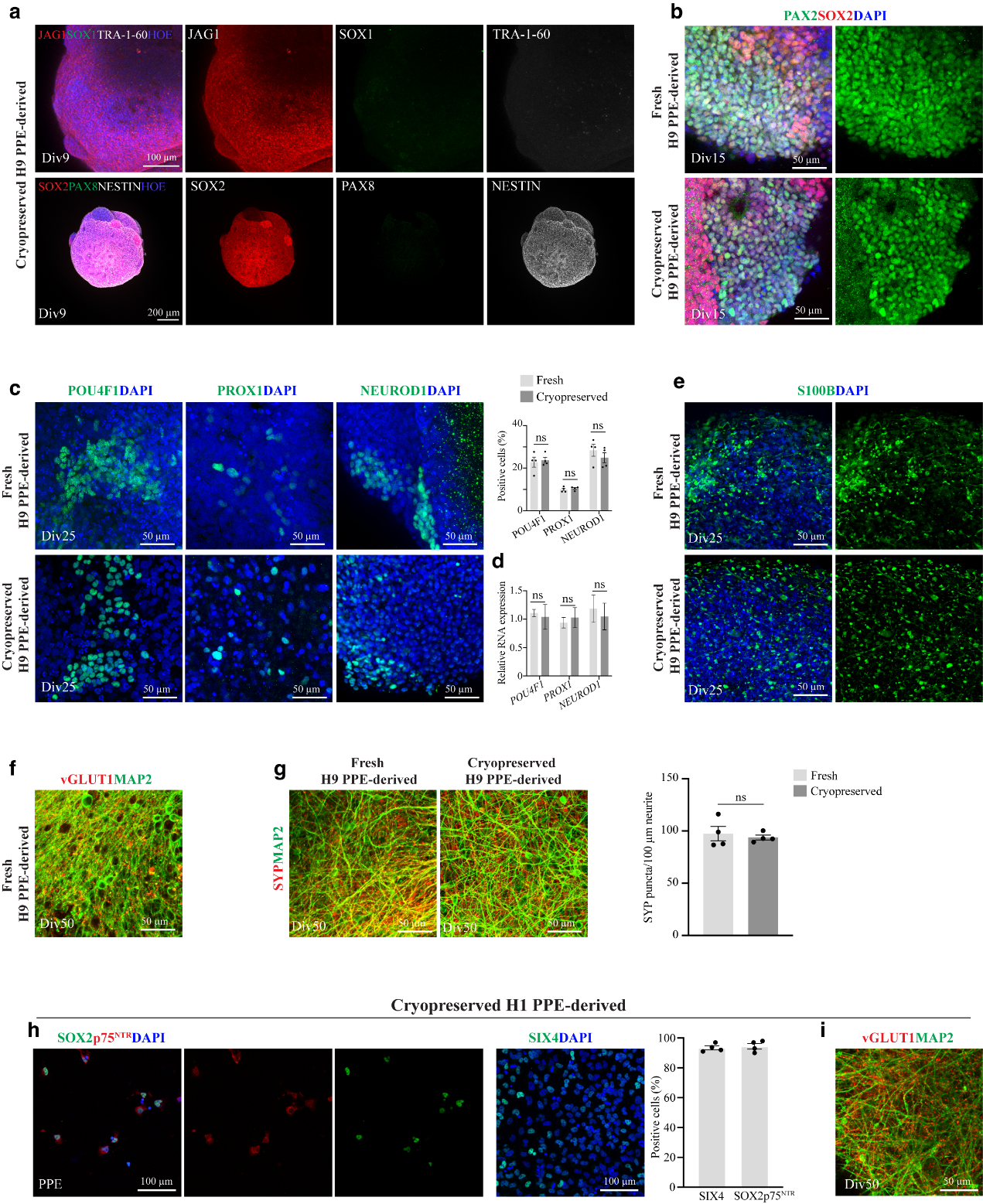


**Figure S2.** Expression of otic and glial markers during the development of otic spheroids. **a)** Immunostaining of early otic marker JAG1 (up) and other confocal channels of Figure 1c (down). Pluripotent marker, TRA-1-60. **b)** Immunostaining of ONP markers PAX2 and SOX2 at Div15 hONS. **c)** Immunostaining and quantification of initial SGN transcriptional factors, POU4F1, PROX1 and NEUROD1 at Div25 hONS. Unpaired two-tailed Student’s *t*-test. n = 4 spheroids for each group. **d)** Relative mRNA expression of *POU4F1*, *PROX1* and *NEUROD1* at Div25 hONS. Unpaired two-tailed Student’s *t*-test. n = 3 samples for each group. **e)** Immunostaining of glial marker S100b, at Div25 hONS. **f)** Immunostaining of mature glutamatergic neurons (MAP2 and vGLUT1) at Div50 hONS generated using fresh H9 PPE cells. **g)** Immunostaining and quantification of synaptic vesicles (SYP-positive) density in mature neurons (MAP2-positive) at Div50 hONS generated using H9 PPE approaches (fresh and cryopreserved groups). Unpaired two-tailed Student’s *t*-test. n = 4 spheroids for each group. **h-i)** Verification of PPE identity (SOX2, p75^NTR^, and SIX4) and mature glutamatergic neurons (MAP2 and vGLUT1) at Div50 hONS generated using cryopreserved H1 PPE cells. n = 4 for each group. Data are presented as mean ± SEM. ns, no significance.

Figure S3


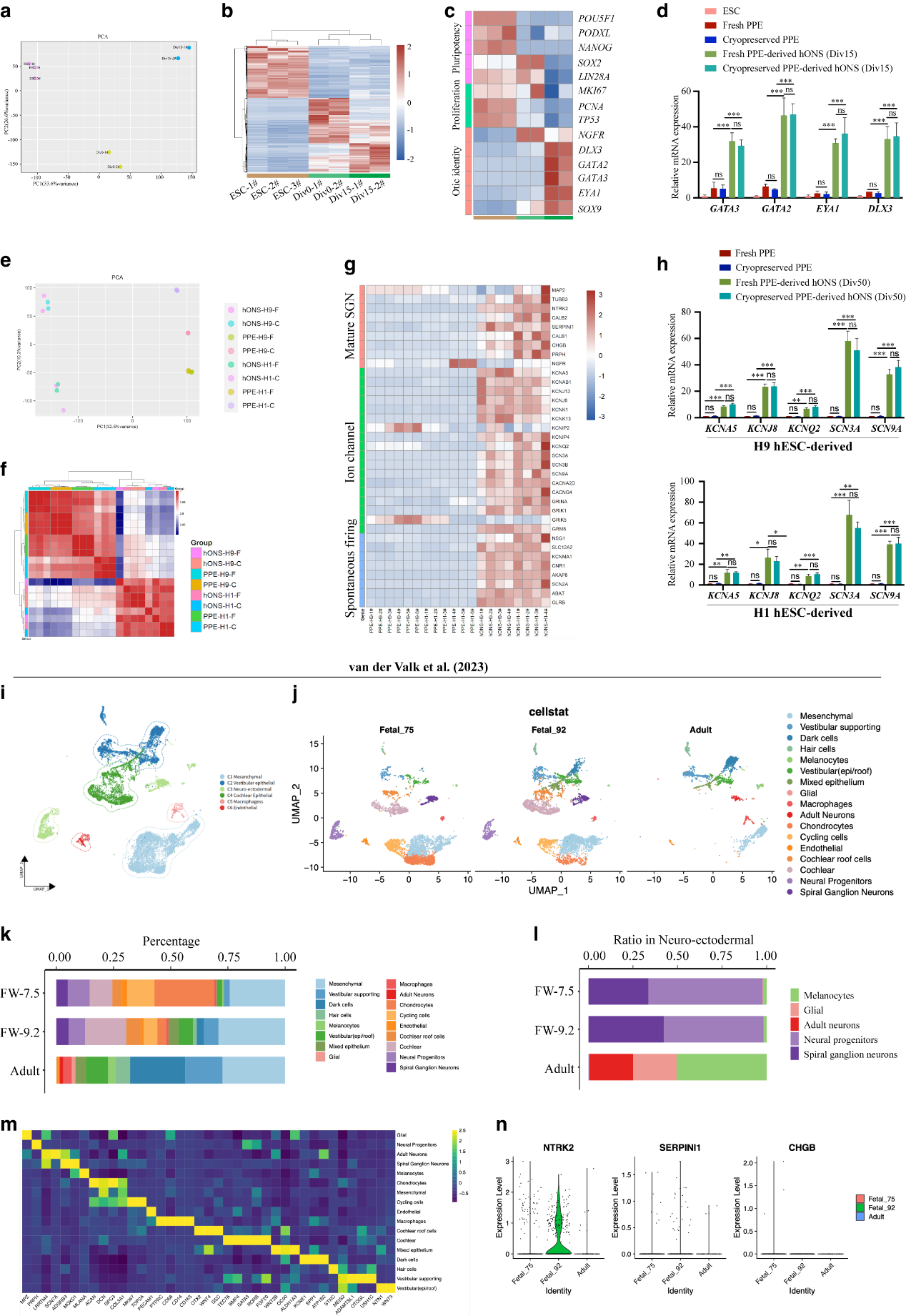


**Figure S3.** Transcriptomic analysis and validation of hONS. **a-b)** PCA (**a**) and clustering analyses (**b**) showing the similarities between samples. hESCs, H9 hESCs from different passages. Div0, thawing cryopreserved PPE cells from two different batches on the first day of spheroid formation. Div15, spheroids generated using two batches of cryopreserved PPE cells derived from H9 hESCs, each containing 3 spheroids. **c)** Expression heatmap of marker genes for pluripotency, proliferation, and otic identity in bulk RNA-seq data of **a**. **d)** Quantification of otic marker genes expression in H9 hESC-derived PPE and Div15 spheroids. One-way ANOVA followed by Tukey’s test. n=3 samples for each group. **e-f)** PCA (**e**) and clustering heatmaps (**f**) showing the similarities between samples. PPE-H9-F, fresh differentiated PPE cells using H9 hESCs. PPE-H9-C, cryopreserved PPE cells generated using H9 hESCs. hONS-H9-F, Div50 hONS generated using fresh H9 hESC-derived PPEs. hONS-H9-C, Div50 hONS generated using cryopreserved H9 hESC-derived PPEs. PPE-H1-C, cryopreserved PPE cells generated using H1 hESCs. hONS-H1-F, Div50 hONS generated using fresh H1 hESC-derived PPEs. hONS-H9-C, Div50 hONS generated using cryopreserved H1 hESC-derived PPEs. **g)** Expression heatmaps of SGN marker, ion channel, and spontaneous firing related genes in hONS. **h)** Quantification of voltage-gated ion channel genes expression in H9 hESC- and H1 hESC-derived PPE and Div50 spheroids. One-way ANOVA followed by Tukey’s test. n=3 samples for each group. **i)** Major cell type annotations of integrated human inner ear datasets using uniform manifold approximation and projection (UMAP) plot. **j)** UMAP plot showing the distribution of single cell types across three developmental timepoints (Fetal_75, Fetal_92, and Adult). **k)** Barplot showing the proportion of each cell type within three developmental timepoints. **l)** Barplot showing the proportion of neuro-ectodermal subtypes within the total neuro-ectodermal population for three developmental timepoints. **m)** Heatmap showing marker genes specifically expressed in each single cell subpopulation. **n)** SGN Marker gene expression in the spiral ganglion neuron population. **p*< 0.05, ***p*< 0.01, ****p*< 0.001. ns, no significance.

Figure S4


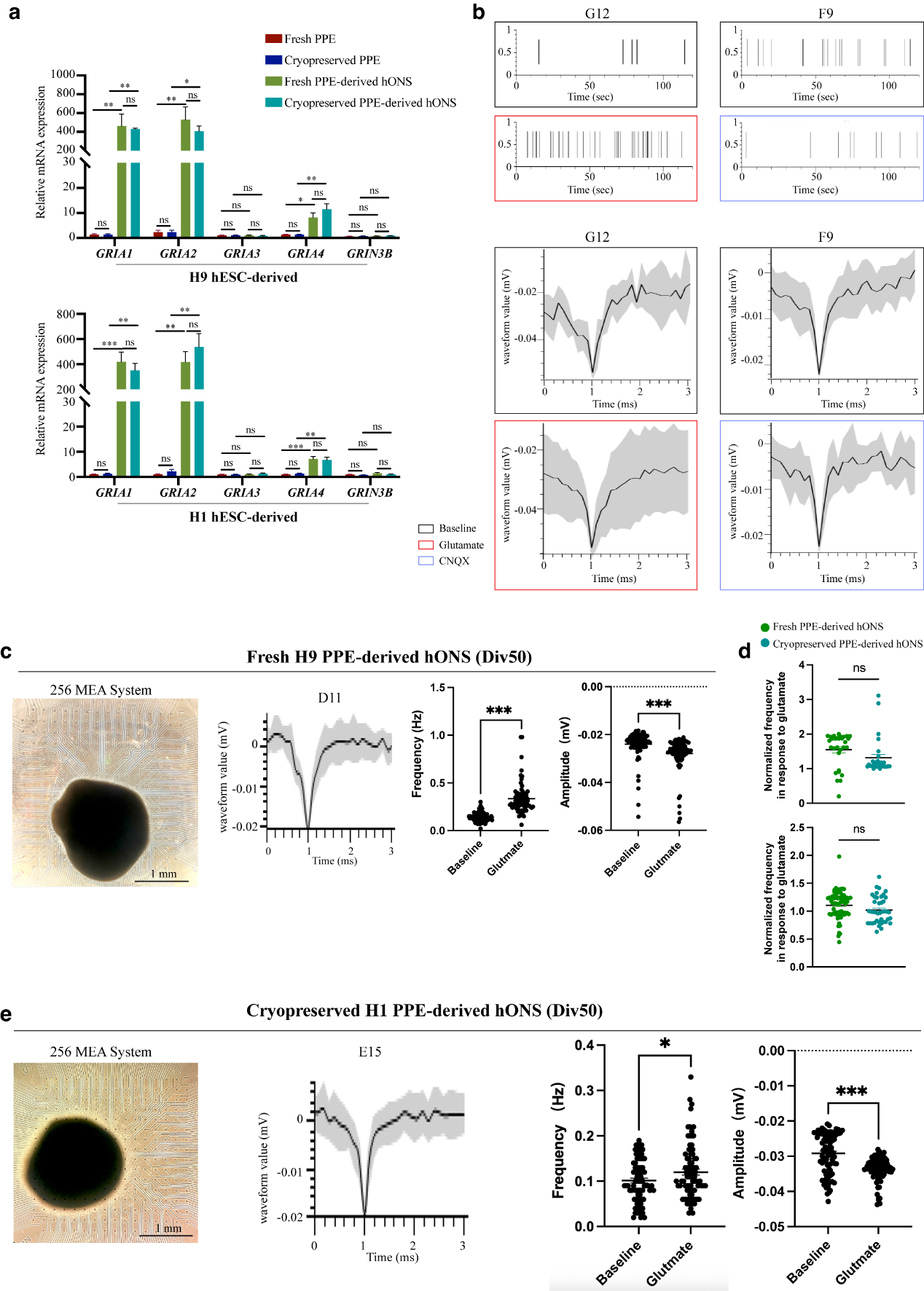


**Figure S4.** Electrophysiological changes in Matrigel-encapsuled otic spheroids under glutamatergic stimulation. **a)** Quantification of AMPA and NMDA receptor genes expression in H9 hESC- and H1 hESC-derived PPE and Div50 spheroids. One-way ANOVA followed by Tukey’s test. n=3 samples for each group. **b)** Frequency and stacked amplitudes of spikes detected in indicated electrodes under glutamate stimulation. **c)** MEA recordings of Div50 hONS generated using fresh PPE cells derived from H9 hESCs. Unpaired two-tailed Student’s *t*-test. n = 87 (Baseline) and 78 (Glutamate) active electrodes for frequency quantification. n = 87 (Baseline) and 86 (Glutamate) active electrodes for amplitude quantification. Values are presented as mean ± SEM. **d)** Comparison of glutamate responses by normalizing amplitude and frequency after glutamate stimulation using baseline values of paired electrodes. Unpaired two-tailed Student’s *t*-test. n = 30 (fresh) and 29 (cryopreserved) electrodes for frequency quantification. n = 72 (fresh) and 41 (cryopreserved) electrodes for amplitude quantification. **e)** MEA recordings of Div50 hONS generated using cryopreserved PPE cells derived from H1 hESCs. Unpaired two-tailed Student’s *t*-test. n = 81 (Baseline) and 86 (Glutamate) active electrodes for frequency quantification. n = 85 (Baseline and Glutamate) active electrodes for amplitude quantification. Values are presented as mean ± SEM. **p*< 0.05, ***p*< 0.01, ****p*< 0.001. ns, no significance.

Figure S5


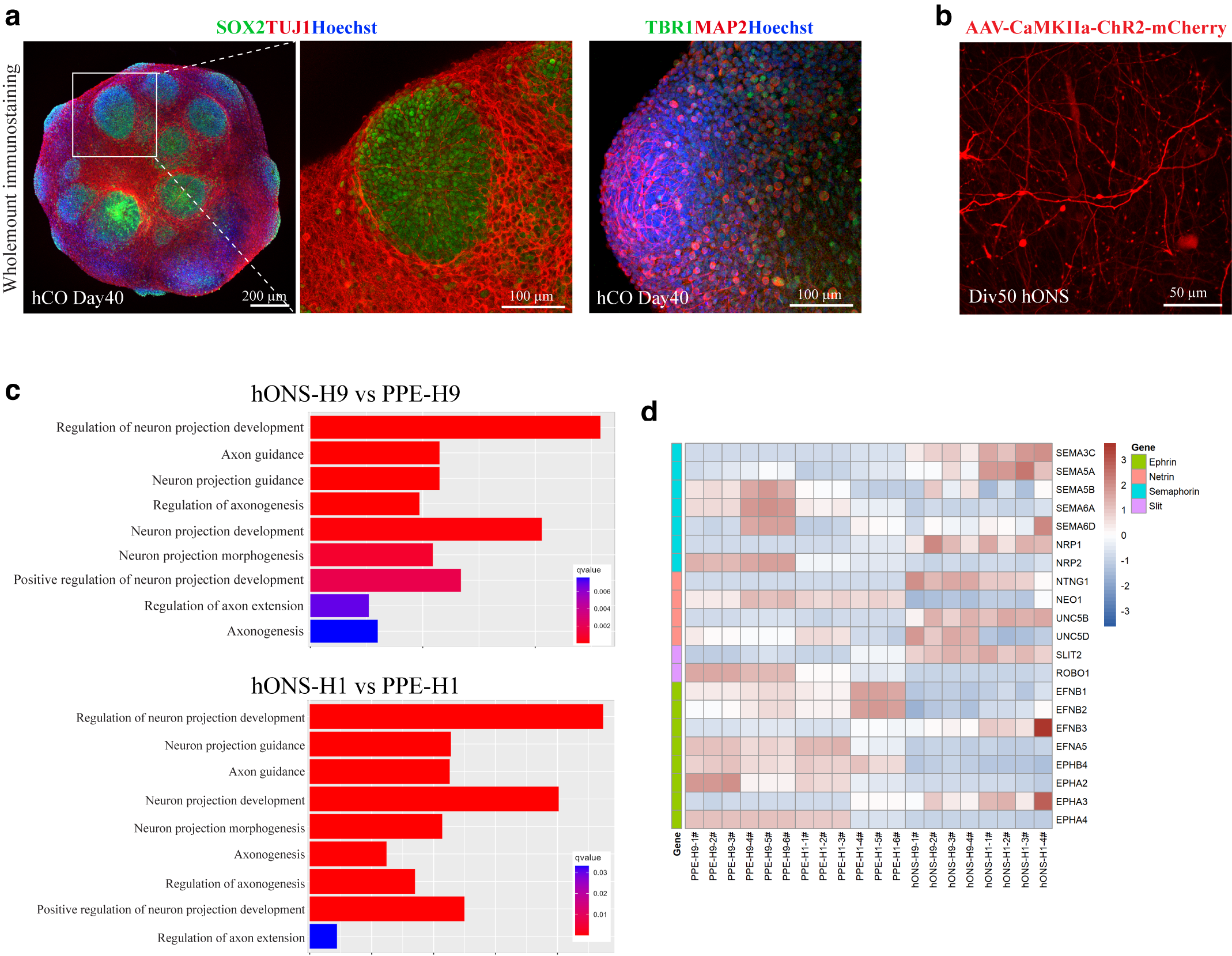


**Figure S5.** Functional projection of neurons in otic spheroids. **a)** Verification of hCO at Day40 via immunostaining of SOX2, TUJ1, TBR1, and MAP2. **b)** Confirmation of ChR2 expression in glutamatergic neurons in Div50 hONS 10-14 days after infection via confocal imaging. **c)** GO analysis of bulk RNA-seq of Div50 otic spheroids (in Figure 2a), enrichment of axon guidance, axon extension, neuron projection, axonogenesis, compared to PPE cells generated using H9 and H1 hESCs, respectively. **d)** Expression heatmaps of axon guidance-related gene family members and receptors from bulk RNA-seq data of hONS.

Figure S6


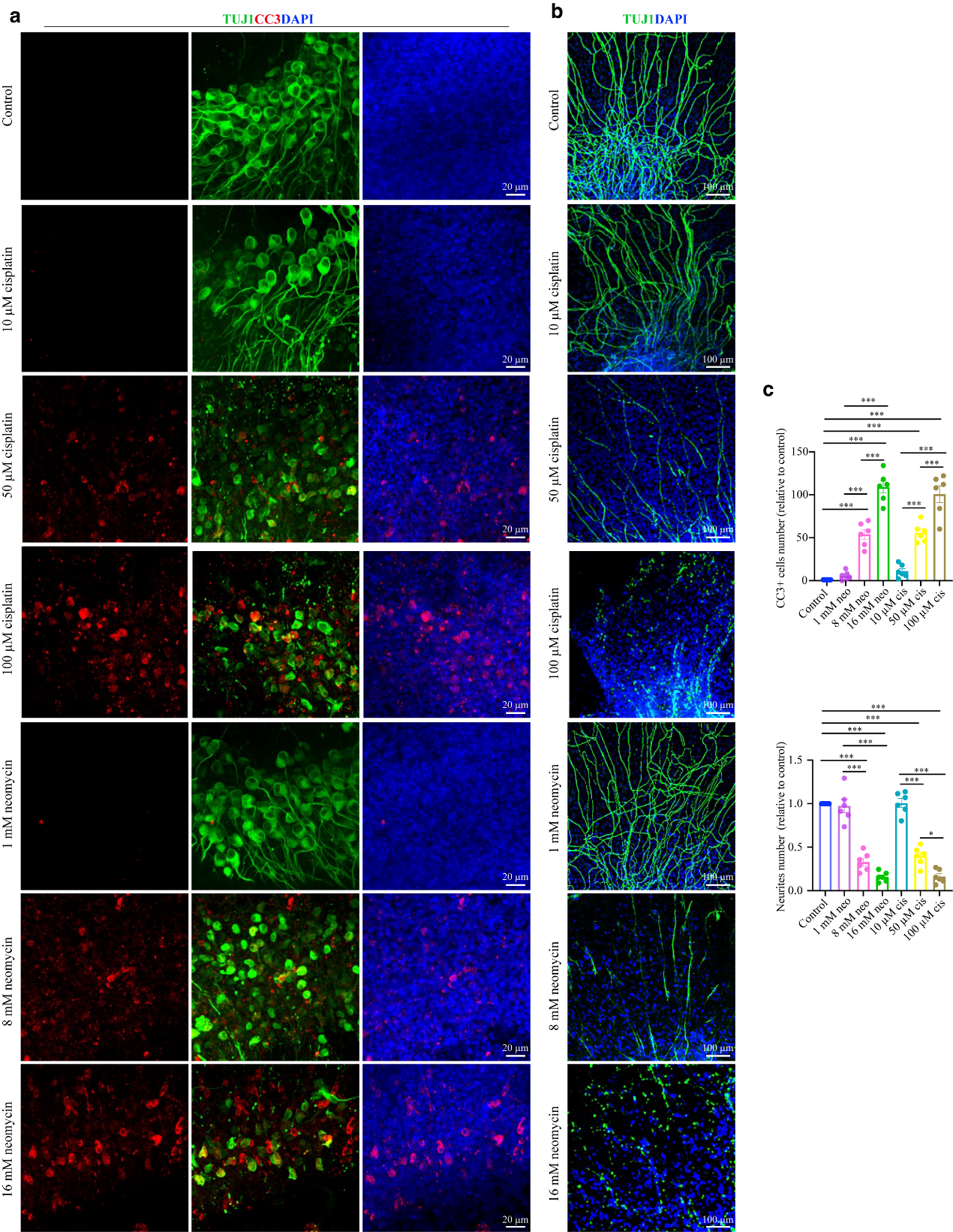


**Figure S6.** Ototoxin treatments to primarily cultured murine spiral ganglion explants. Immunostaining and quantification of apoptotic cells (CC3^+^) and spiral ganglion neuronal neurites (TUJ1^+^) in murine explants treated with different doses of cisplatin or neomycin for 48 h. One-way ANOVA followed by Tukey’s test; n = 6 samples for each group. Values are presented as mean ± SEM. **p*< 0.05, ****p*< 0.001.

Figure S7

**
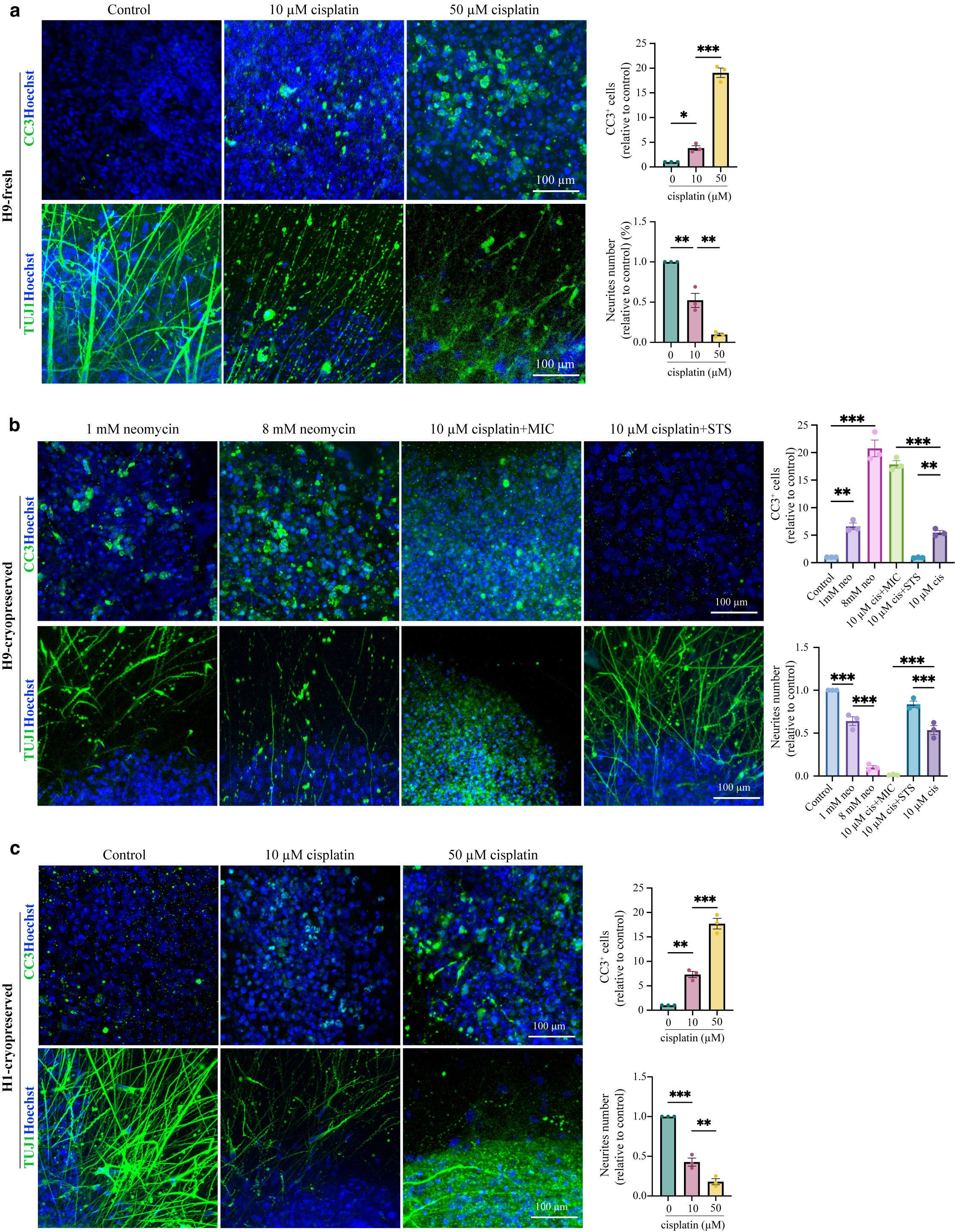
**

**Figure S7.** Ototoxins and otoprotectants treatment to hONS. **a)** Immunostaining and quantification of apoptosis (CC3) and extended neurites (TUJ1) in hONS treated with cisplatin derived using H9-fresh approach. One-way ANOVA followed by Tukey’s test; n= 3 samples for each group. **b)** Immunostaining and quantification of apoptosis (CC3) and extended neurites (TUJ1) in hONS (H9-cryopreserved approach) treated with other ototoxins, neomycin, Minocycline (MIC), or otoprotectant sodium thiosulfate (STS). One-way ANOVA followed by Tukey’s test; n= 3 samples for each group. (**c**) Immunostaining and quantification of apoptosis (CC3) and extended neurites (TUJ1) in hONS treated with cisplatin derived using H1-cryopreserved approach. One-way ANOVA followed by Tukey’s test; n = 3 samples for each group. Values are presented as mean ± SEM. **p*< 0.05, ***p*< 0.01, ****p*< 0.001.

Figure S8


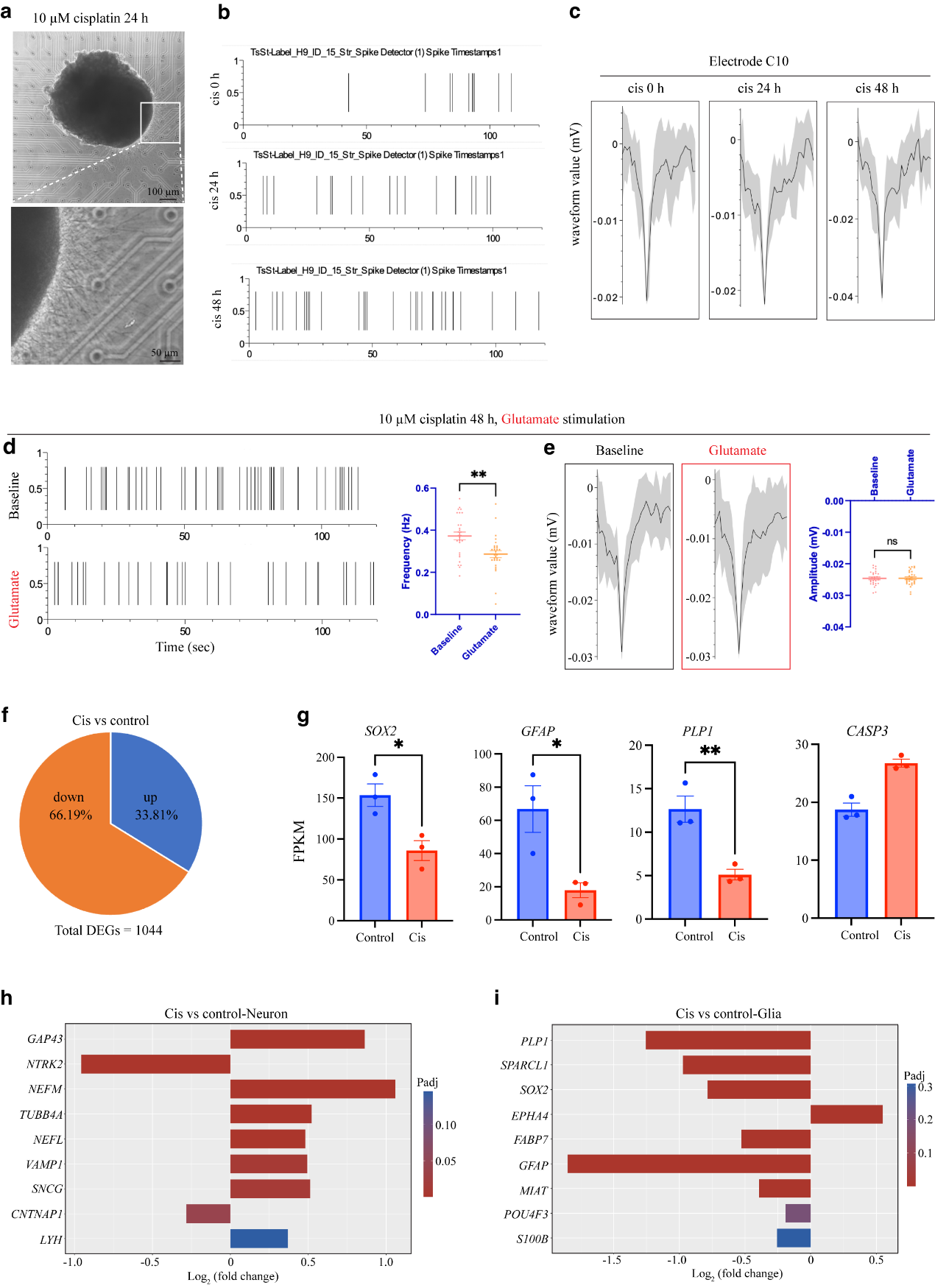


**Figure S8.** Low-dose cisplatin quickly affects electrical activity and transcriptomic profiles of neurite-sprouted hONS along time. **a)** Phase control images showing the extended neurites in a neurite-sprouted otic spheroid mounted on a MEA plate after 24-hour 10 µM cisplatin treatment. **b-c)** Frequency (**b**) and stacked amplitude (**c**) of spikes detected in the indicated electrode under cisplatin treatment. **d-e)** Frequency (**d**) and stacked amplitude (**e**) representations and quantification analyses under glutamatergic stimulation after 48-hour 10 µM cisplatin treatment. Unpaired two-tailed Student’s *t*-test. n = 28 (Baseline) and 29 (Glutamate) active electrodes for frequency quantification, n = 27 (Baseline) and 28 (Glutamate) active electrodes for amplitude quantification. **f)** Pie chart showing DEGs in 10 µM cisplatin-treated hONS. **g)** FPKM values of glial marker genes, *SOX2,* *GFAP*, and *PLP1*, and apoptotic gene *CASP3*. Unpaired two-tailed Student’s *t*-test; n = 3 samples. **h-i)** Expression of neuron markers (**h**) and glial markers (**i**) in the cisplatin-treated group. Values are presented as mean ± SEM. **p*< 0.05, ***p*< 0.01. ns, no significance.

Figure S9


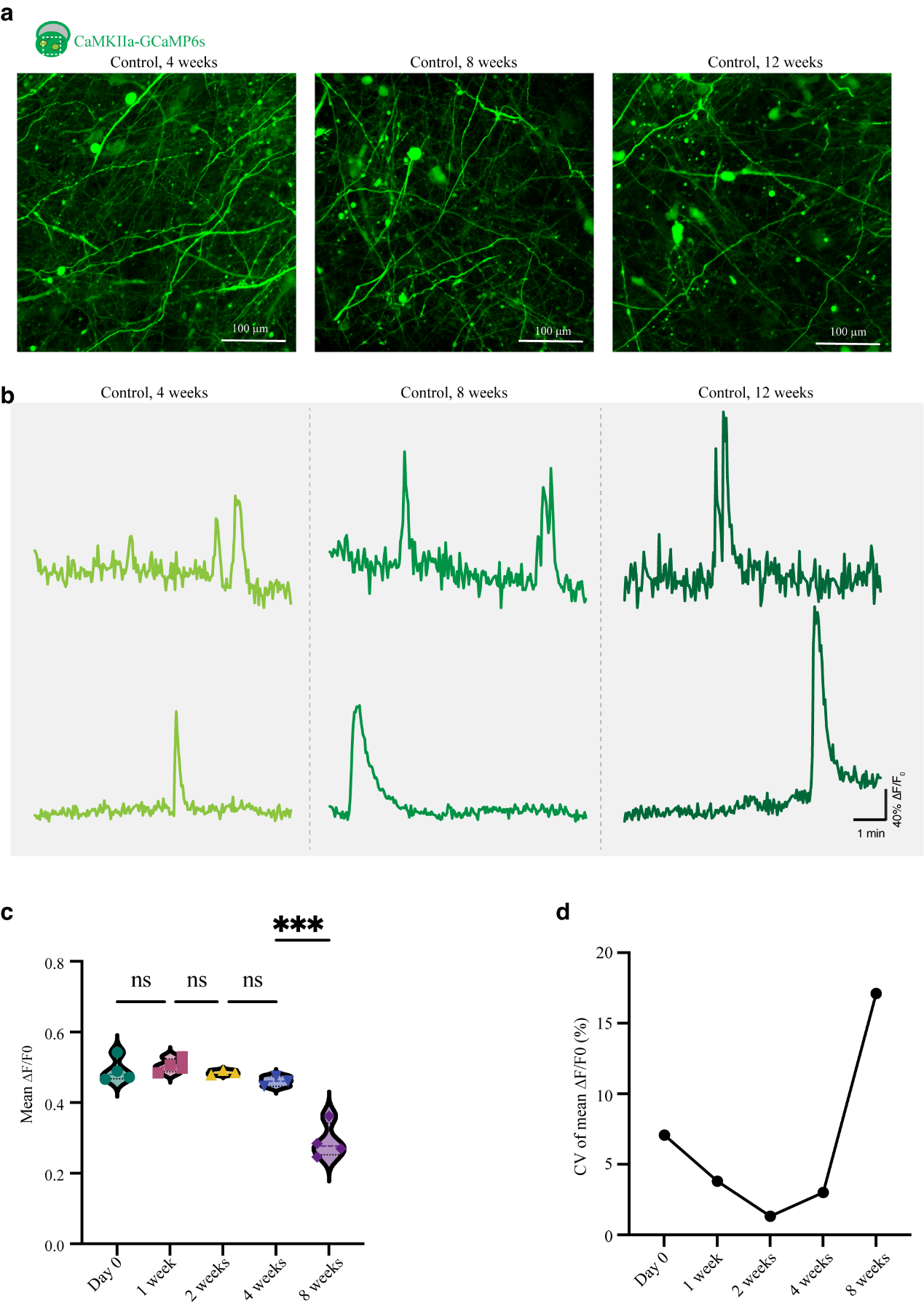


**Figure S9.** Dynamic changes of calcium activity of glutamatergic neurons in hONS after long-term treatment with low dose cisplatin. **a-b)** Representative images and calcium traces of virally encoded otic spheroids without treated drug treatment at 4-12 weeks. **c)** Quantification of mean ΔF/F0 ratios per otic spheroid in different groups. One-way ANOVA followed by Tukey’s test. n= 3 to 4 samples for each group. **d)** Coefficient of variation (CV) of mean ΔF/F0 ratios in **c**. Values are presented as mean ± SEM. ****p*< 0.001. ns, no significance.

Figure S10


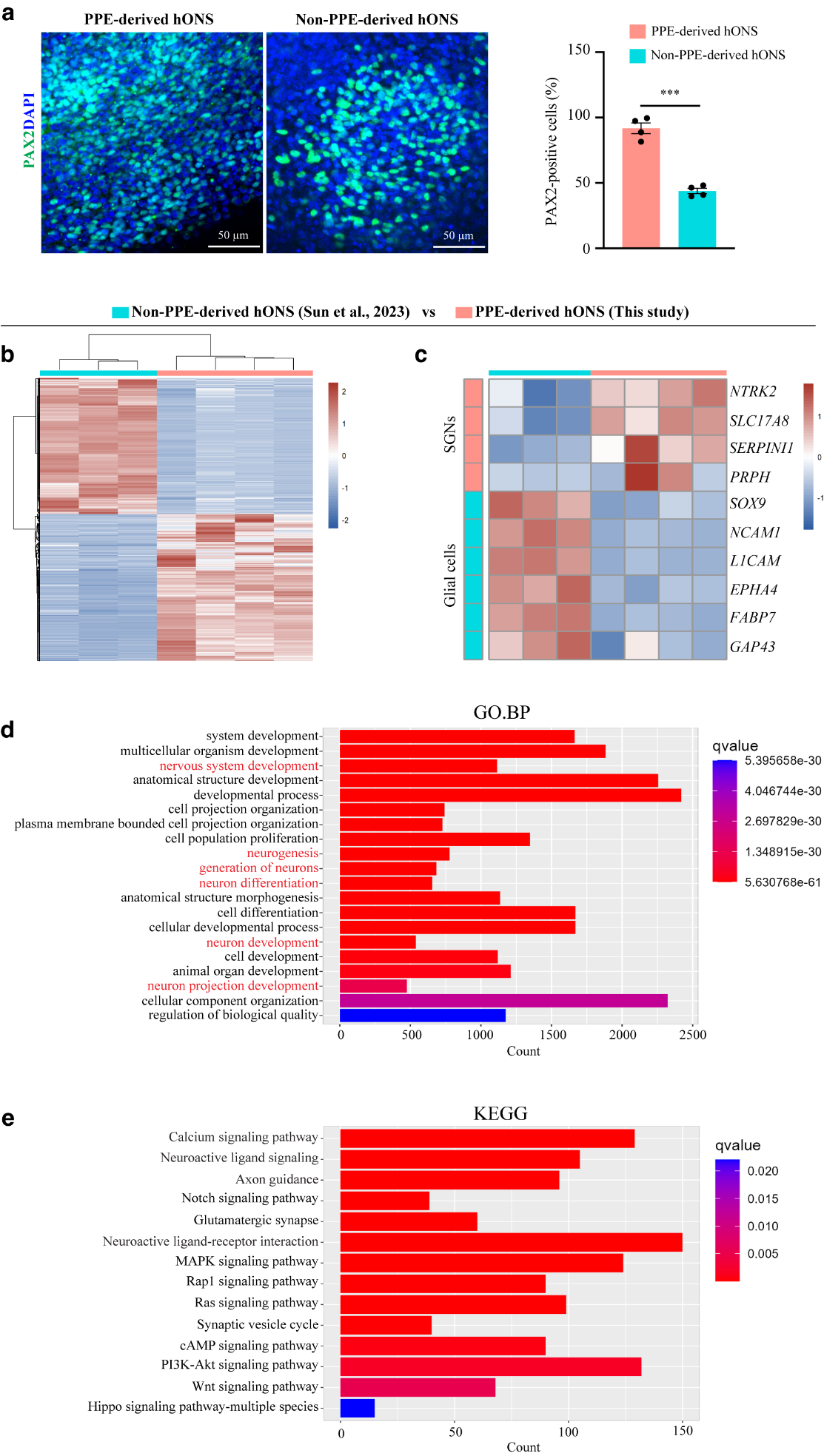


**Figure S10.** Otic marker expression and transcriptomic profiles comparison between PPE- and non-PPE-derived hONS. A comparative analysis of otic marker expression and transcriptomic profiles was conducted between PPE-derived organoids at ONP stage (generated from H9 ESCs in this study) and non-PPE-derived ONP cells (generated from H9 ESCs as described in our previous work^[4]^). **a)** Immunostaining of otic marker PAX2 showed a significantly higher percentage of PAX2-positive cells in PPE-derived organoids. Unpaired two-tailed Student’s *t*-test. n = 4 organoids for each group. **b**) Transcriptomic comparison was carried out using bulk RNA-seq data. Clustering heatmaps showed distinct transcriptional profiles between the two groups. **c**) Expression heatmaps further indicated that SGN marker genes were significantly upregulated in PPE-derived hONS, whereas glial marker genes were downregulated compared to non-PPE-derived hONS. **d-e**) GO (**d**) and KEGG (**e**) analysis showed items enriched in PPE-derived hONS relative to the non-PPE group. Values are presented as mean ± SEM. ****p*< 0.001.

**Tables**

**Table S1.** Antibodies used in this study.

| **Antibody** | **Host** | **Supplier** | **Catalog No.** | **Dilution** |
| --- | --- | --- | --- | --- |
| CALB2 | Chicken | NOVUS | NBP2-50029 | 1:200 |
| CC3 | Rabbit | Cell Signaling | 9579S | 1:200 |
| DLX5 | Rabbit | Abcam | ab109737 | 1:200 |
| ECAD | Mouse | Abcam | ab1416 | 1:500 |
| FOXG1 | Rabbit | Abcam | ab18259 | 1:200 |
| GATA3 | Rat | Thermo | 14-9966-80 | 1:500 |
| JAG1 | Rabbit | Abcam | ab7771 | 1:500 |
| SOX1 | Goat | R&D | AF3369 | 1:200 |
| TUJ1 | Rabbit | Biolegend | 802001 | 1:1000 |
| TUJ1 | Mouse | Biolegend | 801201 | 1:1000 |
| SYP | Rabbit | Abcam | ab32127 | 1:500 |
| SYP | Mouse | Invitrogen | MA1-213 | 1:200 |
| MAP2 | Chicken | Novus | NB300-213 | 1:1500 |
| MAP2 | Mouse | Abcam | ab11267 | 1:500 |
| vGLUT1 | Rabbit | Invitrogen | 48-2400 | 1:200 |
| OCT4 | Mouse | BD Biosciences | 611203 | 1:200 |
| SOX2 | Goat | R&D Systems | AF2018 | 1:200 |
| NANOG | Rabbit | Proteintech | 14295-1 | 1:250 |
| SSEA4 | Mouse | Millipore | MAB4304 | 1:500 |
| NESTIN | Mouse | Millipore | MAB5326 | 1:250 |
| PAX8 | Rabbit | Abcam | ab97477 | 1:200 |
| p75^NTR^ | Rabbit | Cell Signaling | 8238S | 1:500 |
| SOX9 | Rabbit | Abcam | ab185966 | 1:250 |
| TRA-1-60 | Mouse | Sigma | MAB4360 | 1:250 |
| SIX4 | Mouse | Thermo | H00051804-M09 | 1:200 |
| SEMA6D | Rabbit | Thermo | PA5-113249 | 1:200 |
| PAX2 | Rabbit | Thermo | PA5-81235 | 1:200 |
| SCN3A | Rabbit | Thermo | PA5-104465 | 1:200 |
| KCNQ4 | Rabbit | Thermo | PA5-119709 | 1:200 |

**Table S2.** Quantitative PCR primers used in this study

| **Gene** | **Forward sequence (5’-3’)** | **Reverse sequence (5’-3’)** |
| --- | --- | --- |
| *ACTIN* | GCACCACACCTTCTACAATG | TGCTTGCTGATCCACATGTG |
| *GATA3* | TTCTGCCGTACCCAGTTTTT | GGGTCGTTGAATGATTTGCT |
| *EYA1* | TTGAAGCCCTGACCGACTC | TTGCTCCTTGTTCTTCTTCTAC |
| *GATA2* | CAGCAAGGCTCGTTCCTGTT | GGCTTGATGAGTGGTCGGT |
| *DLX3* | TACCCTGCCCGAGTCTTCTG | TGGTGGTAGGTGTAGGGGTTC |
| *KCNA5* | CGCGTCCACATCAACATCTC | GGTAGAAGCGTATCTCGTCCG |
| *KCNJ8* | AACCTGGCGCATAAGAACATC | CCACATGATAGCGAAGAGCAG |
| *KCNQ2* | CATTGGCTACGGGGACAAGTA | GGGTTCCGCCTCTTCTCAA |
| *SCN3A* | GGAGAGCTGTTGGAAAGTTCTT | TTCCTTCGGTTCCTCCATTCT |
| *SCN9A* | CCTCAGAGCTTTGTCCATTTCA | TTGTCTGCATAGTAGGGGTCC |
| *GRIA1* | CGAGCTTTCCCGTTGATACAT | TCTGCCACTTGTAATGGTCAATG |
| *GRIA2* | CATTCAGATGAGACCCGACCT | GGTATGCAAACTTGTCCCATTGA |
| *GRIA3* | ACCATCAGCATAGGTGGACTT | GGTTGGTGTTGTATAACTGCACG |
| *GRIA4* | TTCCGAGCAGCGTGCAAATA | GCATTGGGGCTGGTGTTATGA |
| *GRIN3B* | TAACGCTGTTGGAACACCCAT | CAGTAGCCGTAGCAGCACT |
| *POU4F1* | CACGCCGCCGCTGCAGAGCAAC | GTACGTGGCGTCCGGCTTGA |
| *PROX1* | AATGACTTTGAGGTTCCAGA | AATCTCAGGGACTTCACTATC |
| *NEUROD1* | TCGAGACTCTGCGCTTGGCC | GTCCGAGGATTGAGTTGCAG |

**Table S3.** Successful rates of organoid formation for two PPE-derived approaches.

| **Approach** | **hPSC line** | **Vials** | **Spheroids-formed wells / Total wells** | **Spheroids-formed rate** |
| --- | --- | --- | --- | --- |
| Fresh | H9 hESCs | H9-Fresh1 | 96/96 | 100% |
|  |  | H9-Fresh2 | 94/96 | 97.92% |
|  |  | H9-Fresh3 | 140/144 | 97.22% |
| Cryopreserved | H9 hESCs | H9-Cryo1-Thaw1 | 95/96 | 98.96% |
|  |  | H9-Cryo1-Thaw2 | 189/192 | 98.44% |
|  |  | H9-Cryo2-Thaw1 | 93/96 | 96.88% |
|  |  | H9-Cryo2-Thaw2 | 109/112 | 97.32% |
| Cryopreserved | H1 hESCs | H1-Cryo1-Thaw1 | 96/96 | 100% |
|  |  | H1-Cryo1-Thaw2 | 101/104 | 97.12% |
|  |  | H1-Cryo2-Thaw1 | 79/80 | 98.75% |
|  |  | H1-Cryo2-Thaw2 | 94/96 | 97.92% |

**References**

1. Chang, H.T., et al., *An engineered three-dimensional stem cell niche in the inner ear by applying a nanofibrillar cellulose hydrogel with a sustained-release neurotrophic factor delivery system.* Acta Biomater, 2020. **108**: p. 111-127.

2. Matsuoka, A.J., et al., *Directed Differentiation of Human Embryonic Stem Cells Toward Placode-Derived Spiral Ganglion-Like Sensory Neurons.* Stem Cells Transl Med, 2017. **6**(3): p. 923-936.

3. Heuer, R.A., et al., *Three-Dimensional Otic Neuronal Progenitor Spheroids Derived from Human Embryonic Stem Cells.* Tissue Eng Part A, 2021. **27**(3-4): p. 256-269.3.

4. Sun, G., et al., *Generation of human otic neuronal organoids using pluripotent stem cells.* Cell Prolif, 2023. **56**(5): p. e13434.
