# Supplementary figures and images for: Cryopreserved Human Otic Neuronal Spheroids Self‐assemble for Functional Connectivity Analysis and Long‐term Ototoxicity Evaluation (part 2 of 2)
Source: Adv Sci (Weinh). 2025 Nov 21;13(7):e05663. doi: 10.1002/advs.202505663 (PMC12866767; doi:10.1002/advs.202505663)

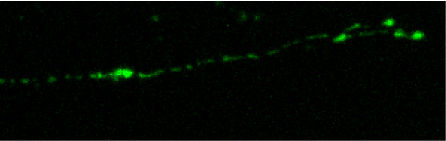

Supplement: Supplementary file 2 — Supplemental Data [file ADVS-13-e05663-s002.zip › advs72932-sup-0001-Data/fig7/Fig7i-10uM cisplatin-TUJ1.jpg]

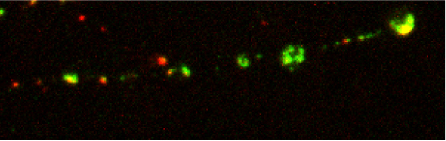

Supplement: Supplementary file 2 — Supplemental Data [file ADVS-13-e05663-s002.zip › advs72932-sup-0001-Data/fig7/Fig7i-50uM cisplatin-SYP+TUJ1.jpg]

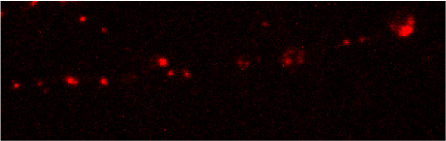

Supplement: Supplementary file 2 — Supplemental Data [file ADVS-13-e05663-s002.zip › advs72932-sup-0001-Data/fig7/Fig7i-50uM cisplatin-SYP.jpg]

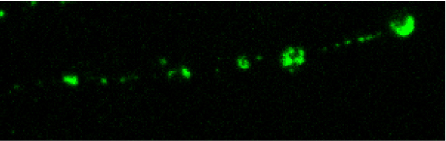

Supplement: Supplementary file 2 — Supplemental Data [file ADVS-13-e05663-s002.zip › advs72932-sup-0001-Data/fig7/Fig7i-50uM cisplatin-TUJ1.jpg]

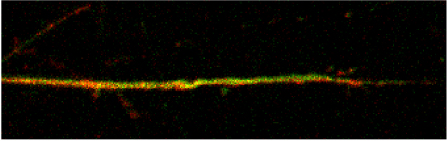

Supplement: Supplementary file 2 — Supplemental Data [file ADVS-13-e05663-s002.zip › advs72932-sup-0001-Data/fig7/Fig7i-control-SYP+TUJ1.jpg]

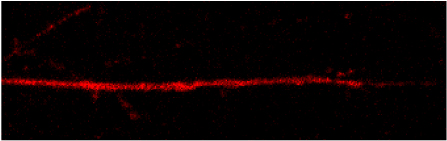

Supplement: Supplementary file 2 — Supplemental Data [file ADVS-13-e05663-s002.zip › advs72932-sup-0001-Data/fig7/Fig7i-control-SYP.jpg]

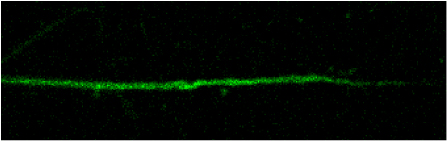

Supplement: Supplementary file 2 — Supplemental Data [file ADVS-13-e05663-s002.zip › advs72932-sup-0001-Data/fig7/Fig7i-control-TUJ1.jpg]

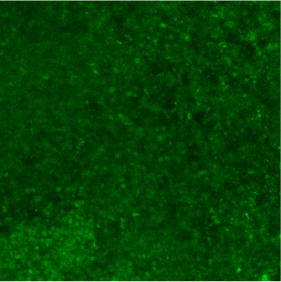

Supplement: Supplementary file 2 — Supplemental Data [file ADVS-13-e05663-s002.zip › advs72932-sup-0001-Data/fig9/Fig9b-12weeks.jpg]

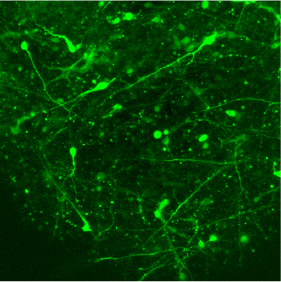

Supplement: Supplementary file 2 — Supplemental Data [file ADVS-13-e05663-s002.zip › advs72932-sup-0001-Data/fig9/Fig9b-1weeks.jpg]

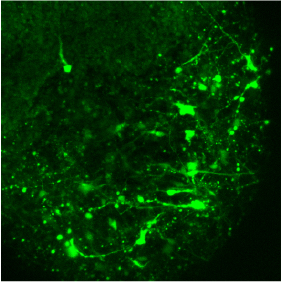

Supplement: Supplementary file 2 — Supplemental Data [file ADVS-13-e05663-s002.zip › advs72932-sup-0001-Data/fig9/Fig9b-2 weeks.jpg]

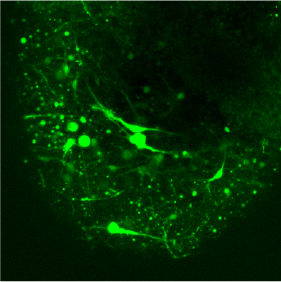

Supplement: Supplementary file 2 — Supplemental Data [file ADVS-13-e05663-s002.zip › advs72932-sup-0001-Data/fig9/Fig9b-4weeks.jpg]

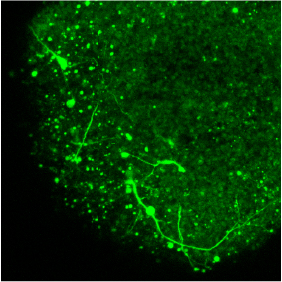

Supplement: Supplementary file 2 — Supplemental Data [file ADVS-13-e05663-s002.zip › advs72932-sup-0001-Data/fig9/Fig9b-8 weeks.jpg]

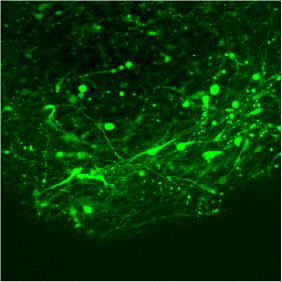

Supplement: Supplementary file 2 — Supplemental Data [file ADVS-13-e05663-s002.zip › advs72932-sup-0001-Data/fig9/Fig9b-day0.jpg]

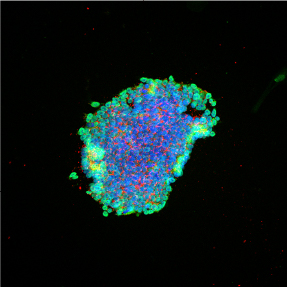

Supplement: Supplementary file 2 — Supplemental Data [file ADVS-13-e05663-s002.zip › advs72932-sup-0001-Data/figs1/Fig S1b-H1-NANOG+SSEA4+DAPI.jpg]

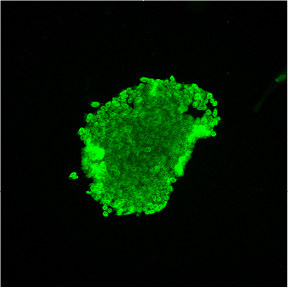

Supplement: Supplementary file 2 — Supplemental Data [file ADVS-13-e05663-s002.zip › advs72932-sup-0001-Data/figs1/Fig S1b-H1-NANOG.jpg]

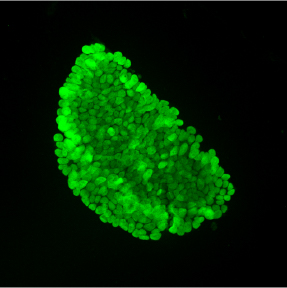

Supplement: Supplementary file 2 — Supplemental Data [file ADVS-13-e05663-s002.zip › advs72932-sup-0001-Data/figs1/Fig S1b-H1-OCT4.jpg]

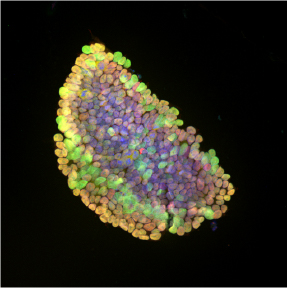

Supplement: Supplementary file 2 — Supplemental Data [file ADVS-13-e05663-s002.zip › advs72932-sup-0001-Data/figs1/Fig S1b-H1-SOX2+OCT4+DAPI.jpg]

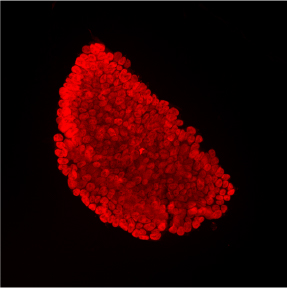

Supplement: Supplementary file 2 — Supplemental Data [file ADVS-13-e05663-s002.zip › advs72932-sup-0001-Data/figs1/Fig S1b-H1-SOX2.jpg]

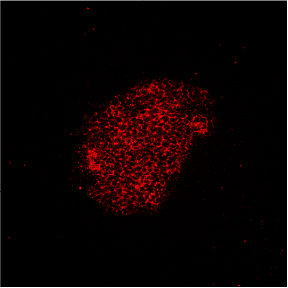

Supplement: Supplementary file 2 — Supplemental Data [file ADVS-13-e05663-s002.zip › advs72932-sup-0001-Data/figs1/Fig S1b-H1-SSEA4.jpg]

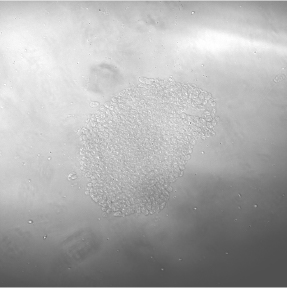

Supplement: Supplementary file 2 — Supplemental Data [file ADVS-13-e05663-s002.zip › advs72932-sup-0001-Data/figs1/Fig S1b-H1BF.jpg]

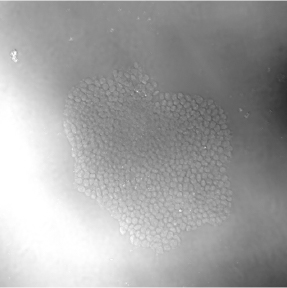

Supplement: Supplementary file 2 — Supplemental Data [file ADVS-13-e05663-s002.zip › advs72932-sup-0001-Data/figs1/Fig S1b-H9-BF.jpg]

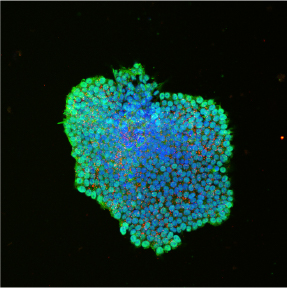

Supplement: Supplementary file 2 — Supplemental Data [file ADVS-13-e05663-s002.zip › advs72932-sup-0001-Data/figs1/Fig S1b-H9-NANOG+SSEA4+DAPI.jpg]

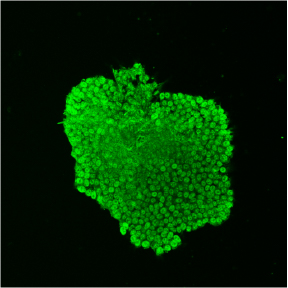

Supplement: Supplementary file 2 — Supplemental Data [file ADVS-13-e05663-s002.zip › advs72932-sup-0001-Data/figs1/Fig S1b-H9-NANOG.jpg]

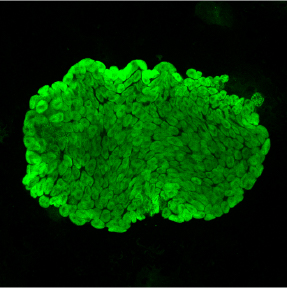

Supplement: Supplementary file 2 — Supplemental Data [file ADVS-13-e05663-s002.zip › advs72932-sup-0001-Data/figs1/Fig S1b-H9-OCT4.jpg]

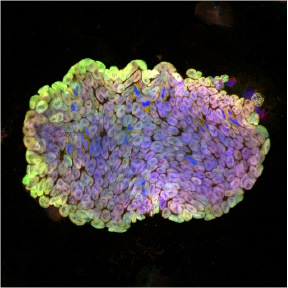

Supplement: Supplementary file 2 — Supplemental Data [file ADVS-13-e05663-s002.zip › advs72932-sup-0001-Data/figs1/Fig S1b-H9-SOX2+OCT4+DAPI.jpg]

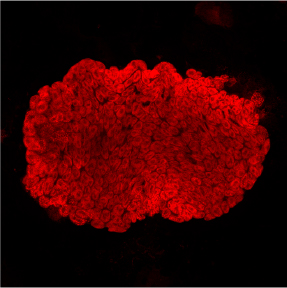

Supplement: Supplementary file 2 — Supplemental Data [file ADVS-13-e05663-s002.zip › advs72932-sup-0001-Data/figs1/Fig S1b-H9-SOX2.jpg]

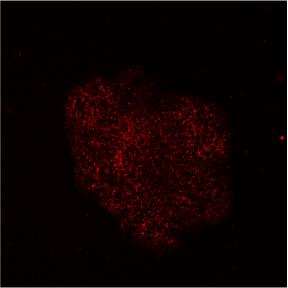

Supplement: Supplementary file 2 — Supplemental Data [file ADVS-13-e05663-s002.zip › advs72932-sup-0001-Data/figs1/Fig S1b-H9-SSEA4.jpg]

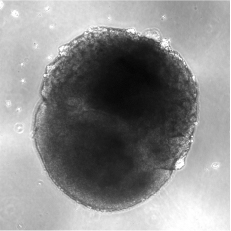

Supplement: Supplementary file 2 — Supplemental Data [file ADVS-13-e05663-s002.zip › advs72932-sup-0001-Data/figs1/Fig S1c-Div100.jpg]

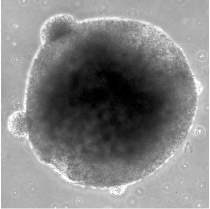

Supplement: Supplementary file 2 — Supplemental Data [file ADVS-13-e05663-s002.zip › advs72932-sup-0001-Data/figs1/Fig S1c-Div15.jpg]

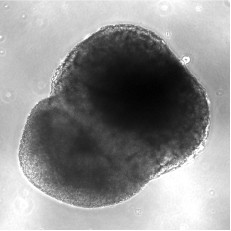

Supplement: Supplementary file 2 — Supplemental Data [file ADVS-13-e05663-s002.zip › advs72932-sup-0001-Data/figs1/Fig S1c-Div150.jpg]

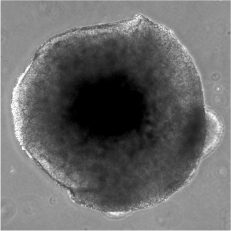

Supplement: Supplementary file 2 — Supplemental Data [file ADVS-13-e05663-s002.zip › advs72932-sup-0001-Data/figs1/Fig S1c-Div18.jpg]

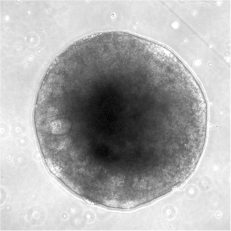

Supplement: Supplementary file 2 — Supplemental Data [file ADVS-13-e05663-s002.zip › advs72932-sup-0001-Data/figs1/Fig S1c-Div25.jpg]

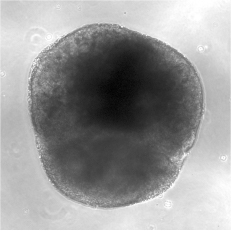

Supplement: Supplementary file 2 — Supplemental Data [file ADVS-13-e05663-s002.zip › advs72932-sup-0001-Data/figs1/Fig S1c-Div30.jpg]

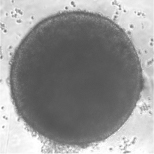

Supplement: Supplementary file 2 — Supplemental Data [file ADVS-13-e05663-s002.zip › advs72932-sup-0001-Data/figs1/Fig S1c-Div4.jpg]

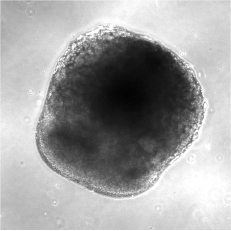

Supplement: Supplementary file 2 — Supplemental Data [file ADVS-13-e05663-s002.zip › advs72932-sup-0001-Data/figs1/Fig S1c-Div40.jpg]

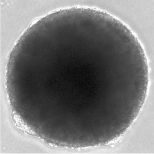

Supplement: Supplementary file 2 — Supplemental Data [file ADVS-13-e05663-s002.zip › advs72932-sup-0001-Data/figs1/Fig S1c-Div7.jpg]

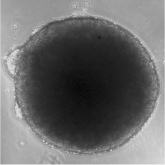

Supplement: Supplementary file 2 — Supplemental Data [file ADVS-13-e05663-s002.zip › advs72932-sup-0001-Data/figs1/Fig S1c-Div9.jpg]

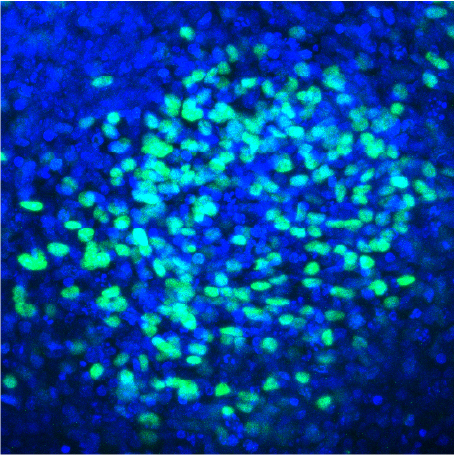

Supplement: Supplementary file 2 — Supplemental Data [file ADVS-13-e05663-s002.zip › advs72932-sup-0001-Data/figs10/Fig S10a-nonPPE.jpg]

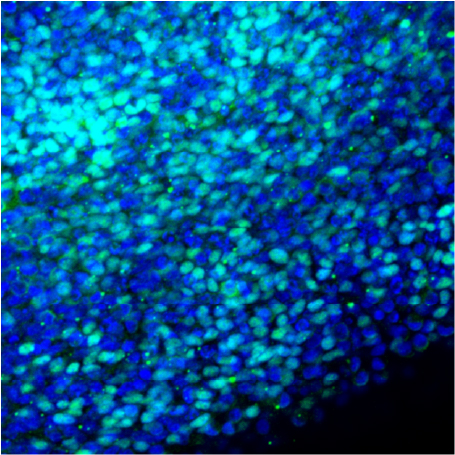

Supplement: Supplementary file 2 — Supplemental Data [file ADVS-13-e05663-s002.zip › advs72932-sup-0001-Data/figs10/Fig S10a-PPE.jpg]

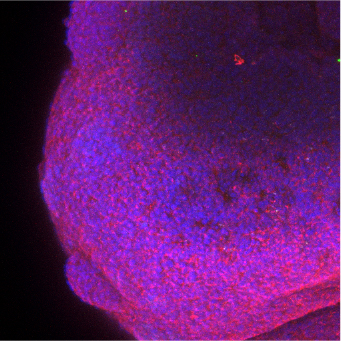

Supplement: Supplementary file 2 — Supplemental Data [file ADVS-13-e05663-s002.zip › advs72932-sup-0001-Data/figs2/Fig S2a-JAG1-SOX1-TRA-HOE.jpg]

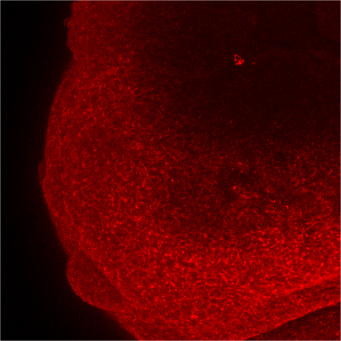

Supplement: Supplementary file 2 — Supplemental Data [file ADVS-13-e05663-s002.zip › advs72932-sup-0001-Data/figs2/Fig S2a-JAG1.jpg]

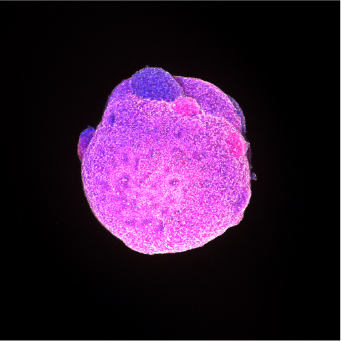

Supplement: Supplementary file 2 — Supplemental Data [file ADVS-13-e05663-s002.zip › advs72932-sup-0001-Data/figs2/Fig S2a-merge.jpg]

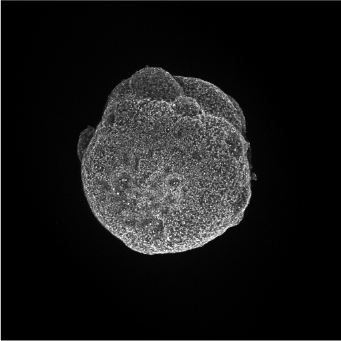

Supplement: Supplementary file 2 — Supplemental Data [file ADVS-13-e05663-s002.zip › advs72932-sup-0001-Data/figs2/Fig S2a-NESTIN.jpg]

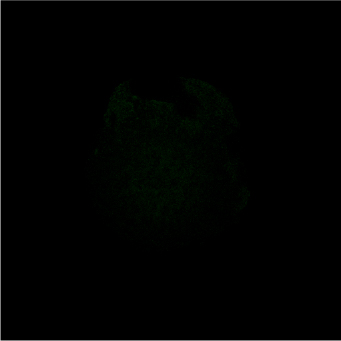

Supplement: Supplementary file 2 — Supplemental Data [file ADVS-13-e05663-s002.zip › advs72932-sup-0001-Data/figs2/Fig S2a-PAX8.jpg]

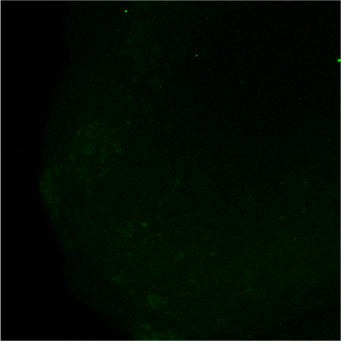

Supplement: Supplementary file 2 — Supplemental Data [file ADVS-13-e05663-s002.zip › advs72932-sup-0001-Data/figs2/Fig S2a-SOX1.jpg]

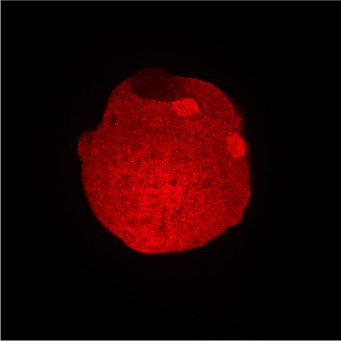

Supplement: Supplementary file 2 — Supplemental Data [file ADVS-13-e05663-s002.zip › advs72932-sup-0001-Data/figs2/Fig S2a-SOX2.jpg]

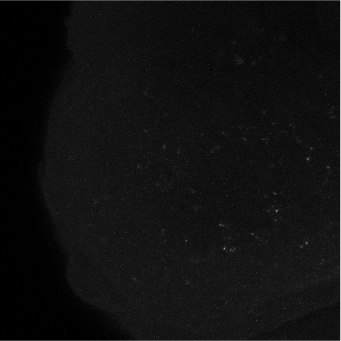

Supplement: Supplementary file 2 — Supplemental Data [file ADVS-13-e05663-s002.zip › advs72932-sup-0001-Data/figs2/Fig S2a-TRA-1-60.jpg]

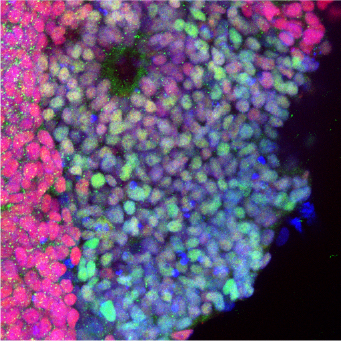

Supplement: Supplementary file 2 — Supplemental Data [file ADVS-13-e05663-s002.zip › advs72932-sup-0001-Data/figs2/Fig S2b-Cryopreserved-PAX2+SOX2+DAPI.jpg]

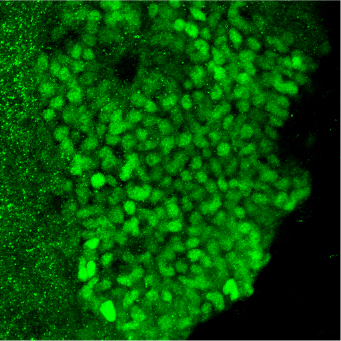

Supplement: Supplementary file 2 — Supplemental Data [file ADVS-13-e05663-s002.zip › advs72932-sup-0001-Data/figs2/Fig S2b-Cryopreserved-PAX2.jpg]

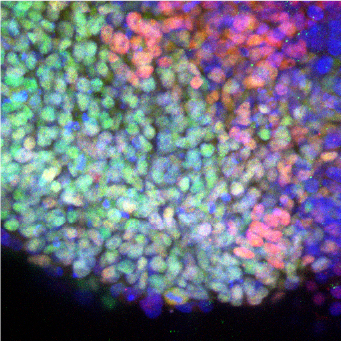

Supplement: Supplementary file 2 — Supplemental Data [file ADVS-13-e05663-s002.zip › advs72932-sup-0001-Data/figs2/Fig S2b-fresh-PAX2+SOX2+DAPI.jpg]

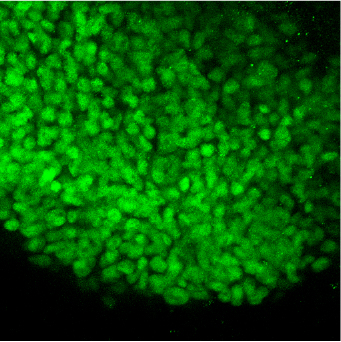

Supplement: Supplementary file 2 — Supplemental Data [file ADVS-13-e05663-s002.zip › advs72932-sup-0001-Data/figs2/Fig S2b-fresh-PAX2.jpg]

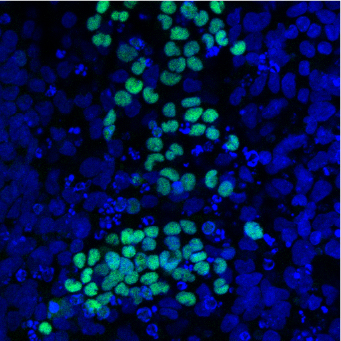

Supplement: Supplementary file 2 — Supplemental Data [file ADVS-13-e05663-s002.zip › advs72932-sup-0001-Data/figs2/Fig S2c-Cryopreserved-POU4F1+DAPI.jpg]

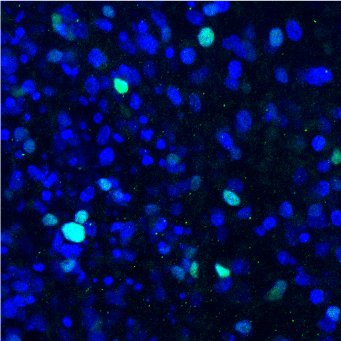

Supplement: Supplementary file 2 — Supplemental Data [file ADVS-13-e05663-s002.zip › advs72932-sup-0001-Data/figs2/Fig S2c-Cryopreserved-PROX1+DAPI.jpg]

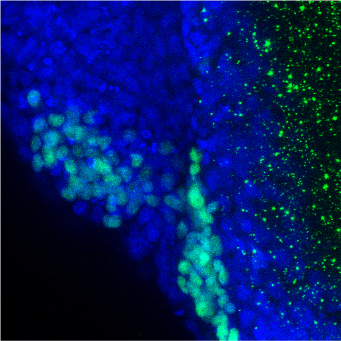

Supplement: Supplementary file 2 — Supplemental Data [file ADVS-13-e05663-s002.zip › advs72932-sup-0001-Data/figs2/Fig S2c-fresh-NEUROD1+DAPI.jpg]

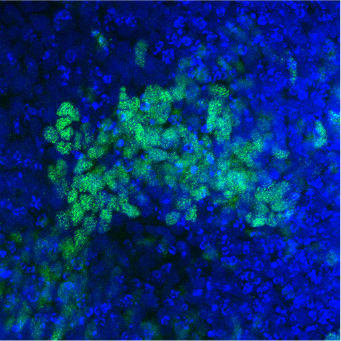

Supplement: Supplementary file 2 — Supplemental Data [file ADVS-13-e05663-s002.zip › advs72932-sup-0001-Data/figs2/Fig S2c-fresh-POU4F1+DAPI.jpg]

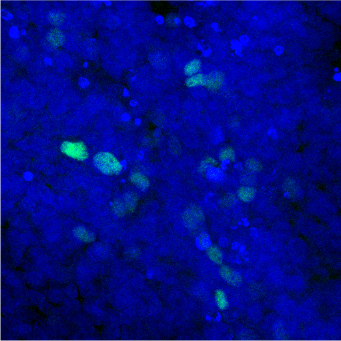

Supplement: Supplementary file 2 — Supplemental Data [file ADVS-13-e05663-s002.zip › advs72932-sup-0001-Data/figs2/Fig S2c-fresh-PROX1+DAPI.jpg]

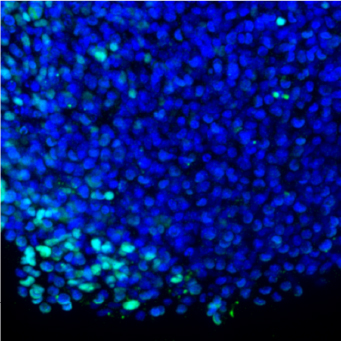

Supplement: Supplementary file 2 — Supplemental Data [file ADVS-13-e05663-s002.zip › advs72932-sup-0001-Data/figs2/Fig S2c-neurod1-cryo.jpg]

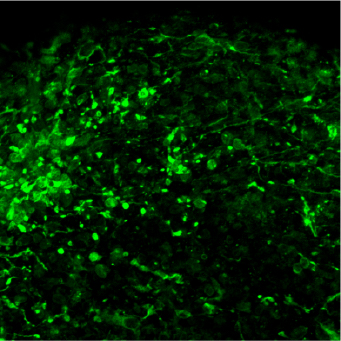

Supplement: Supplementary file 2 — Supplemental Data [file ADVS-13-e05663-s002.zip › advs72932-sup-0001-Data/figs2/Fig s2e-fresh-s100.jpg]

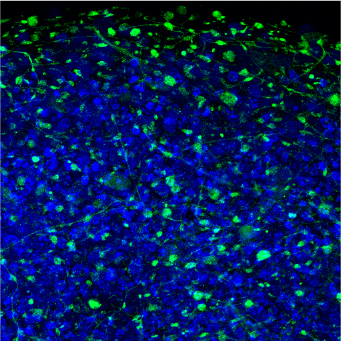

Supplement: Supplementary file 2 — Supplemental Data [file ADVS-13-e05663-s002.zip › advs72932-sup-0001-Data/figs2/Fig S2e-S100-dapi.jpg]

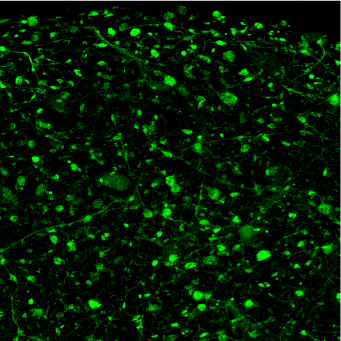

Supplement: Supplementary file 2 — Supplemental Data [file ADVS-13-e05663-s002.zip › advs72932-sup-0001-Data/figs2/Fig S2e-S100.jpg]

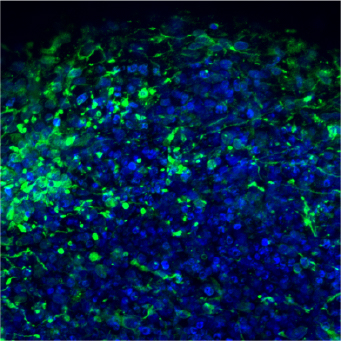

Supplement: Supplementary file 2 — Supplemental Data [file ADVS-13-e05663-s002.zip › advs72932-sup-0001-Data/figs2/fig S2e-s100bdapi.jpg]

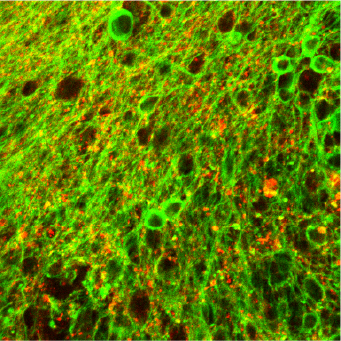

Supplement: Supplementary file 2 — Supplemental Data [file ADVS-13-e05663-s002.zip › advs72932-sup-0001-Data/figs2/Fig S2f-fresh.jpg]

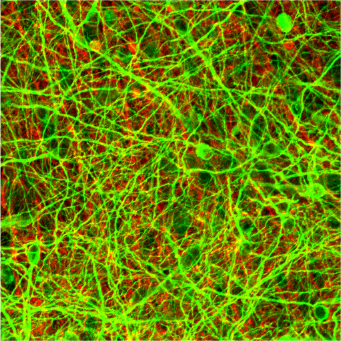

Supplement: Supplementary file 2 — Supplemental Data [file ADVS-13-e05663-s002.zip › advs72932-sup-0001-Data/figs2/Fig S2g-cryo.jpg]

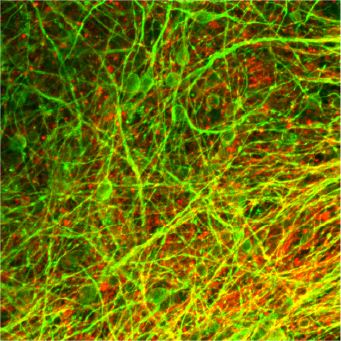

Supplement: Supplementary file 2 — Supplemental Data [file ADVS-13-e05663-s002.zip › advs72932-sup-0001-Data/figs2/Fig S2g-fresh.jpg]

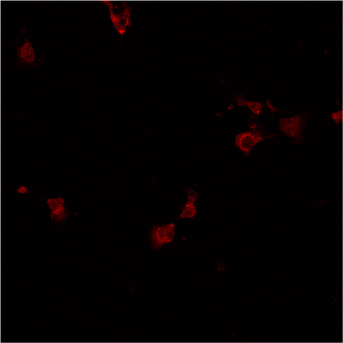

Supplement: Supplementary file 2 — Supplemental Data [file ADVS-13-e05663-s002.zip › advs72932-sup-0001-Data/figs2/Fig S2h-P75NTR.jpg]

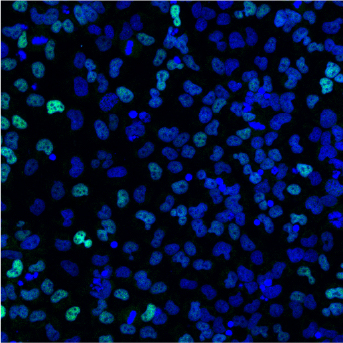

Supplement: Supplementary file 2 — Supplemental Data [file ADVS-13-e05663-s002.zip › advs72932-sup-0001-Data/figs2/Fig S2h-SIX4-DAPI.jpg]

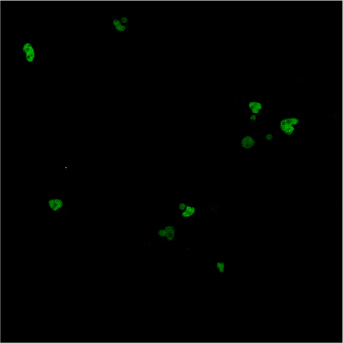

Supplement: Supplementary file 2 — Supplemental Data [file ADVS-13-e05663-s002.zip › advs72932-sup-0001-Data/figs2/Fig S2h-SOX2.jpg]

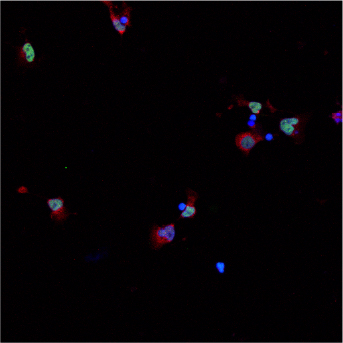

Supplement: Supplementary file 2 — Supplemental Data [file ADVS-13-e05663-s002.zip › advs72932-sup-0001-Data/figs2/Fig S2h-SOX2P75NTRDAPI.jpg]

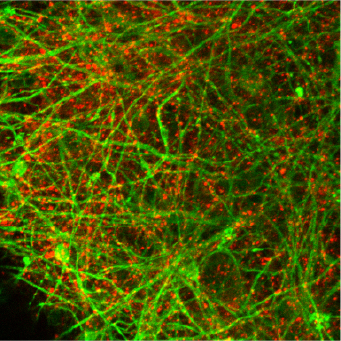

Supplement: Supplementary file 2 — Supplemental Data [file ADVS-13-e05663-s002.zip › advs72932-sup-0001-Data/figs2/Fig S2i.jpg]

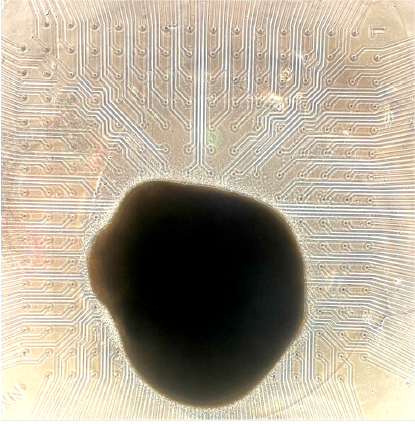

Supplement: Supplementary file 2 — Supplemental Data [file ADVS-13-e05663-s002.zip › advs72932-sup-0001-Data/figs4/figs4c.jpg]

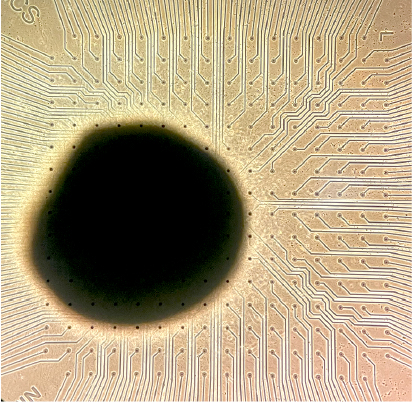

Supplement: Supplementary file 2 — Supplemental Data [file ADVS-13-e05663-s002.zip › advs72932-sup-0001-Data/figs4/figs4e.jpg]

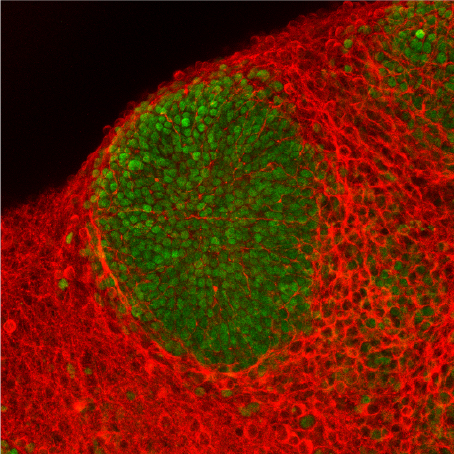

Supplement: Supplementary file 2 — Supplemental Data [file ADVS-13-e05663-s002.zip › advs72932-sup-0001-Data/figs5/Fig S5a-SOX2+TUJ1+DAPI magnification.jpg]

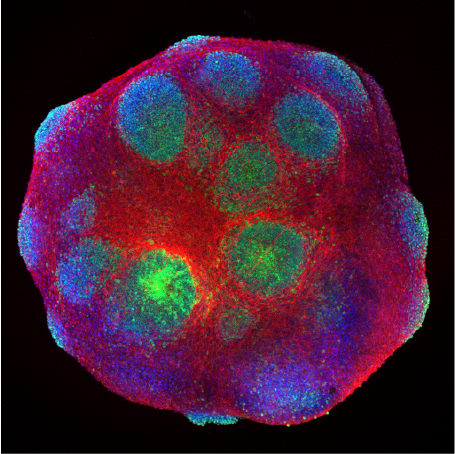

Supplement: Supplementary file 2 — Supplemental Data [file ADVS-13-e05663-s002.zip › advs72932-sup-0001-Data/figs5/Fig S5a-SOX2+TUJ1+DAPI.jpg]

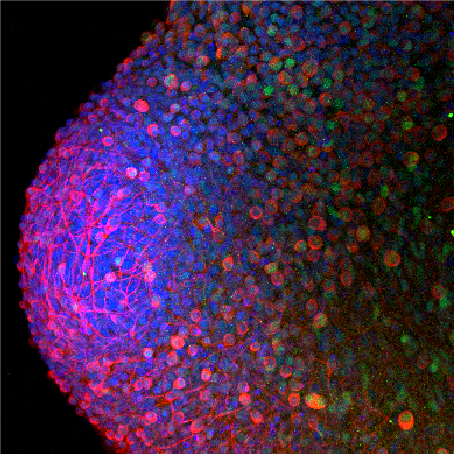

Supplement: Supplementary file 2 — Supplemental Data [file ADVS-13-e05663-s002.zip › advs72932-sup-0001-Data/figs5/Fig S5a-TBR1+MAP2+Hoechst.jpg]

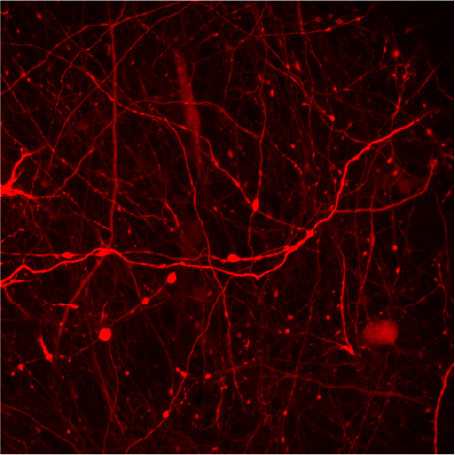

Supplement: Supplementary file 2 — Supplemental Data [file ADVS-13-e05663-s002.zip › advs72932-sup-0001-Data/figs5/Fig S5b.jpg]

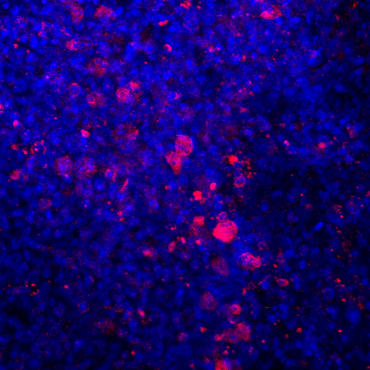

Supplement: Supplementary file 2 — Supplemental Data [file ADVS-13-e05663-s002.zip › advs72932-sup-0001-Data/figs6/FigS6a-Cis100uM-CC3+DAPI.tif]

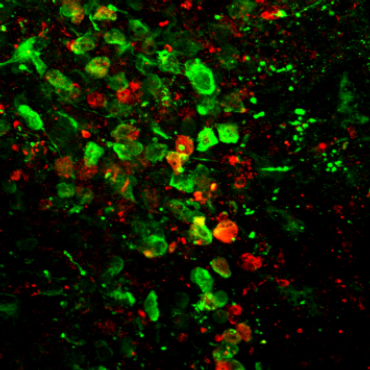

Supplement: Supplementary file 2 — Supplemental Data [file ADVS-13-e05663-s002.zip › advs72932-sup-0001-Data/figs6/FigS6a-Cis100uM-CC3+TUJ1.tif]

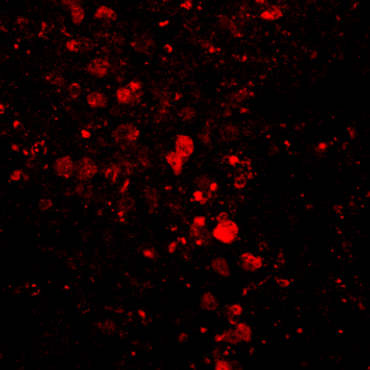

Supplement: Supplementary file 2 — Supplemental Data [file ADVS-13-e05663-s002.zip › advs72932-sup-0001-Data/figs6/FigS6a-Cis100uM-CC3.tif]

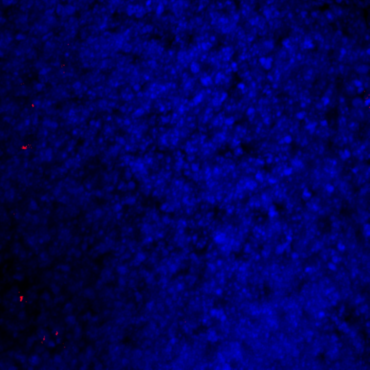

Supplement: Supplementary file 2 — Supplemental Data [file ADVS-13-e05663-s002.zip › advs72932-sup-0001-Data/figs6/FigS6a-cis10uM-CC3+DAPI.tif]

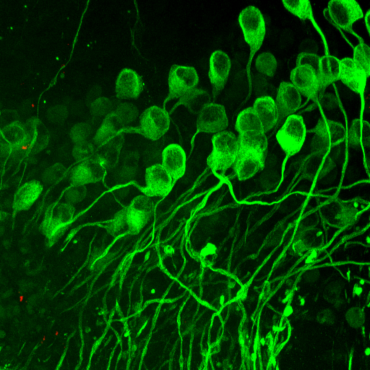

Supplement: Supplementary file 2 — Supplemental Data [file ADVS-13-e05663-s002.zip › advs72932-sup-0001-Data/figs6/FigS6a-cis10uM-CC3+TUJ1.tif]

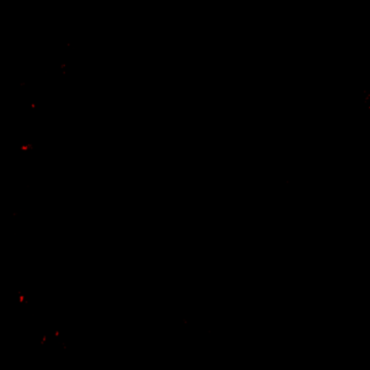

Supplement: Supplementary file 2 — Supplemental Data [file ADVS-13-e05663-s002.zip › advs72932-sup-0001-Data/figs6/FigS6a-cis10uM-CC3.tif]

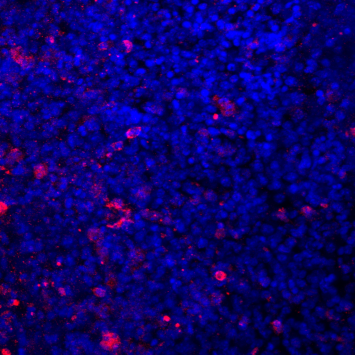

Supplement: Supplementary file 2 — Supplemental Data [file ADVS-13-e05663-s002.zip › advs72932-sup-0001-Data/figs6/FigS6a-Cis50uM-CC3+DAPI.tif]

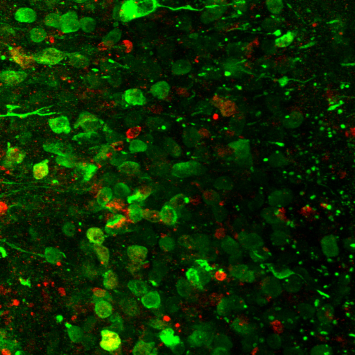

Supplement: Supplementary file 2 — Supplemental Data [file ADVS-13-e05663-s002.zip › advs72932-sup-0001-Data/figs6/FigS6a-Cis50uM-CC3+TUJ1.tif]

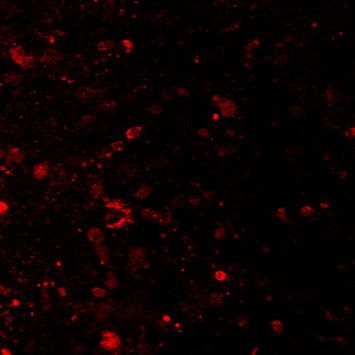

Supplement: Supplementary file 2 — Supplemental Data [file ADVS-13-e05663-s002.zip › advs72932-sup-0001-Data/figs6/FigS6a-Cis50uM-CC3.tif]

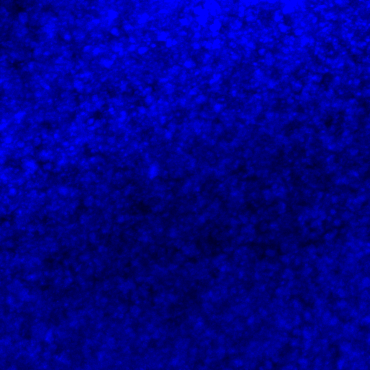

Supplement: Supplementary file 2 — Supplemental Data [file ADVS-13-e05663-s002.zip › advs72932-sup-0001-Data/figs6/FigS6a-Control-CC3+DAPI.tif]

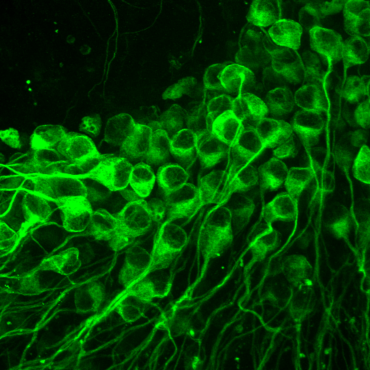

Supplement: Supplementary file 2 — Supplemental Data [file ADVS-13-e05663-s002.zip › advs72932-sup-0001-Data/figs6/FigS6a-Control-CC3+TUJ1.tif]

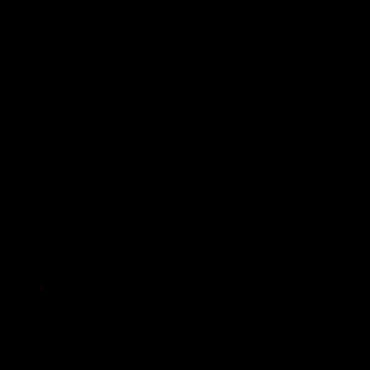

Supplement: Supplementary file 2 — Supplemental Data [file ADVS-13-e05663-s002.zip › advs72932-sup-0001-Data/figs6/FigS6a-Control-CC3.tif]

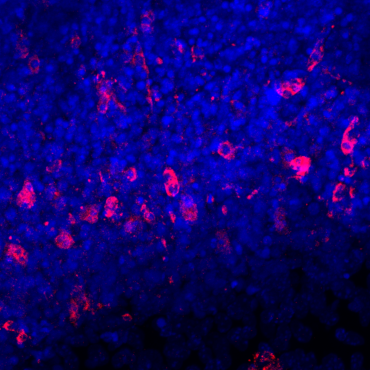

Supplement: Supplementary file 2 — Supplemental Data [file ADVS-13-e05663-s002.zip › advs72932-sup-0001-Data/figs6/FigS6a-Neo16mM-CC3+DAPI.tif]

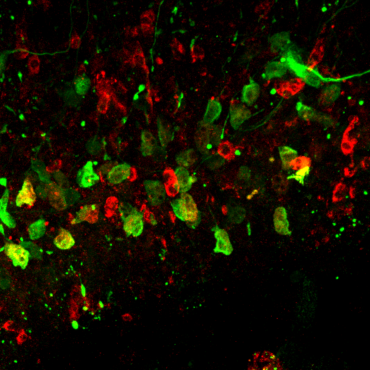

Supplement: Supplementary file 2 — Supplemental Data [file ADVS-13-e05663-s002.zip › advs72932-sup-0001-Data/figs6/FigS6a-Neo16mM-CC3+TUJ1.tif]

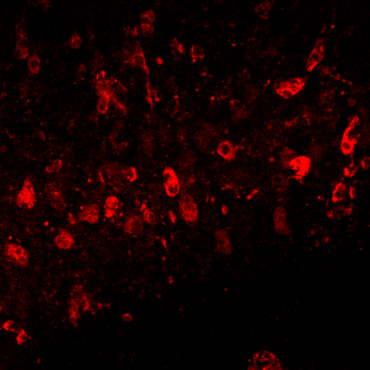

Supplement: Supplementary file 2 — Supplemental Data [file ADVS-13-e05663-s002.zip › advs72932-sup-0001-Data/figs6/FigS6a-Neo16mM-CC3.tif]

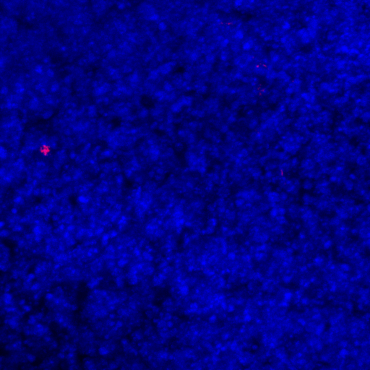

Supplement: Supplementary file 2 — Supplemental Data [file ADVS-13-e05663-s002.zip › advs72932-sup-0001-Data/figs6/FigS6a-Neo1mM-CC3+DAPI.tif]

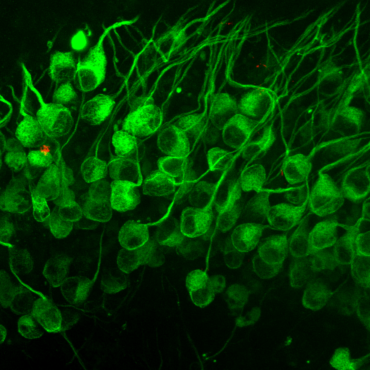

Supplement: Supplementary file 2 — Supplemental Data [file ADVS-13-e05663-s002.zip › advs72932-sup-0001-Data/figs6/FigS6a-Neo1mM-CC3+TUJ1.tif]

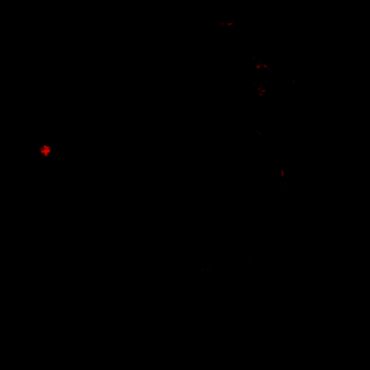

Supplement: Supplementary file 2 — Supplemental Data [file ADVS-13-e05663-s002.zip › advs72932-sup-0001-Data/figs6/FigS6a-Neo1mM-CC3.tif]

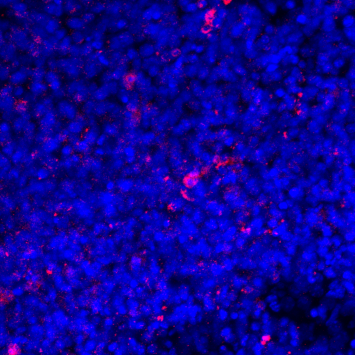

Supplement: Supplementary file 2 — Supplemental Data [file ADVS-13-e05663-s002.zip › advs72932-sup-0001-Data/figs6/FigS6a-Neo8mM-CC3+DAPI.tif]

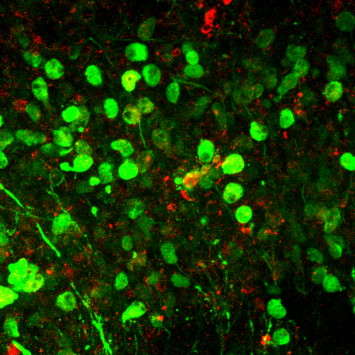

Supplement: Supplementary file 2 — Supplemental Data [file ADVS-13-e05663-s002.zip › advs72932-sup-0001-Data/figs6/FigS6a-Neo8mM-CC3+TUJ1.tif]

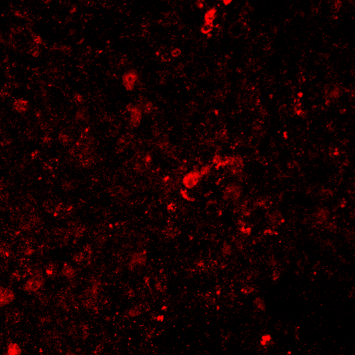

Supplement: Supplementary file 2 — Supplemental Data [file ADVS-13-e05663-s002.zip › advs72932-sup-0001-Data/figs6/FigS6a-Neo8mM-CC3.tif]

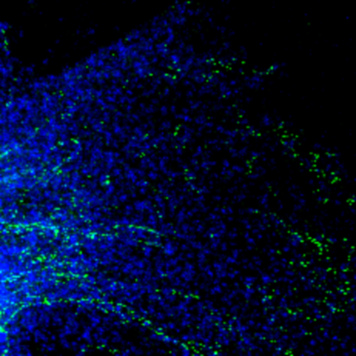

Supplement: Supplementary file 2 — Supplemental Data [file ADVS-13-e05663-s002.zip › advs72932-sup-0001-Data/figs6/FigS6b-cis100uM.tif]

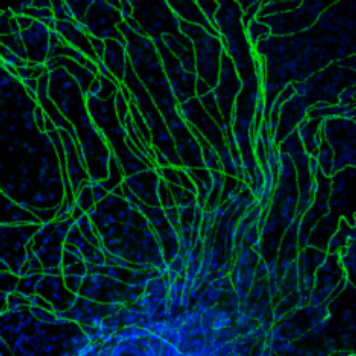

Supplement: Supplementary file 2 — Supplemental Data [file ADVS-13-e05663-s002.zip › advs72932-sup-0001-Data/figs6/FigS6b-cis10uM.tif]

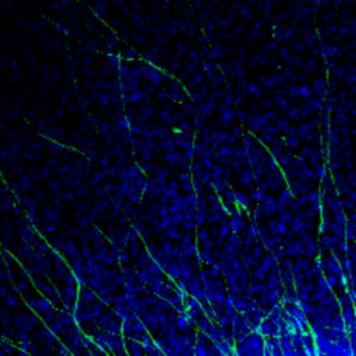

Supplement: Supplementary file 2 — Supplemental Data [file ADVS-13-e05663-s002.zip › advs72932-sup-0001-Data/figs6/FigS6b-cis50uM.tif]

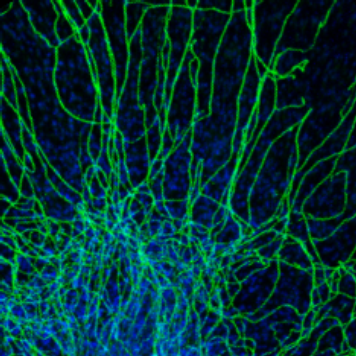

Supplement: Supplementary file 2 — Supplemental Data [file ADVS-13-e05663-s002.zip › advs72932-sup-0001-Data/figs6/FigS6b-control.tif]
