# Supplementary figures and images for: Cryopreserved Human Otic Neuronal Spheroids Self‐assemble for Functional Connectivity Analysis and Long‐term Ototoxicity Evaluation (part 1 of 2)
Source: Adv Sci (Weinh). 2025 Nov 21;13(7):e05663. doi: 10.1002/advs.202505663 (PMC12866767; doi:10.1002/advs.202505663)

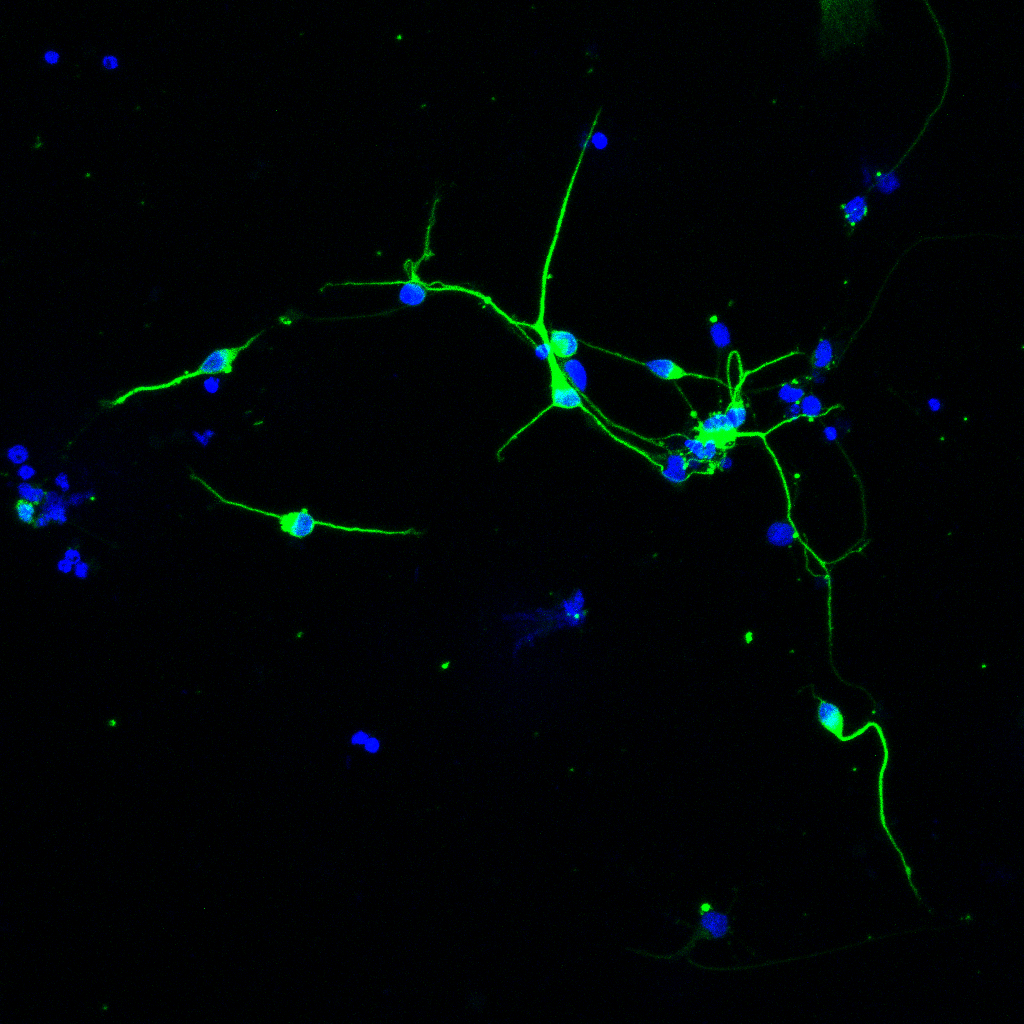

Supplement: Supplementary file 2 — Supplemental Data [file ADVS-13-e05663-s002.zip › advs72932-sup-0001-Data/fig1/fig1-bipolar.tif]

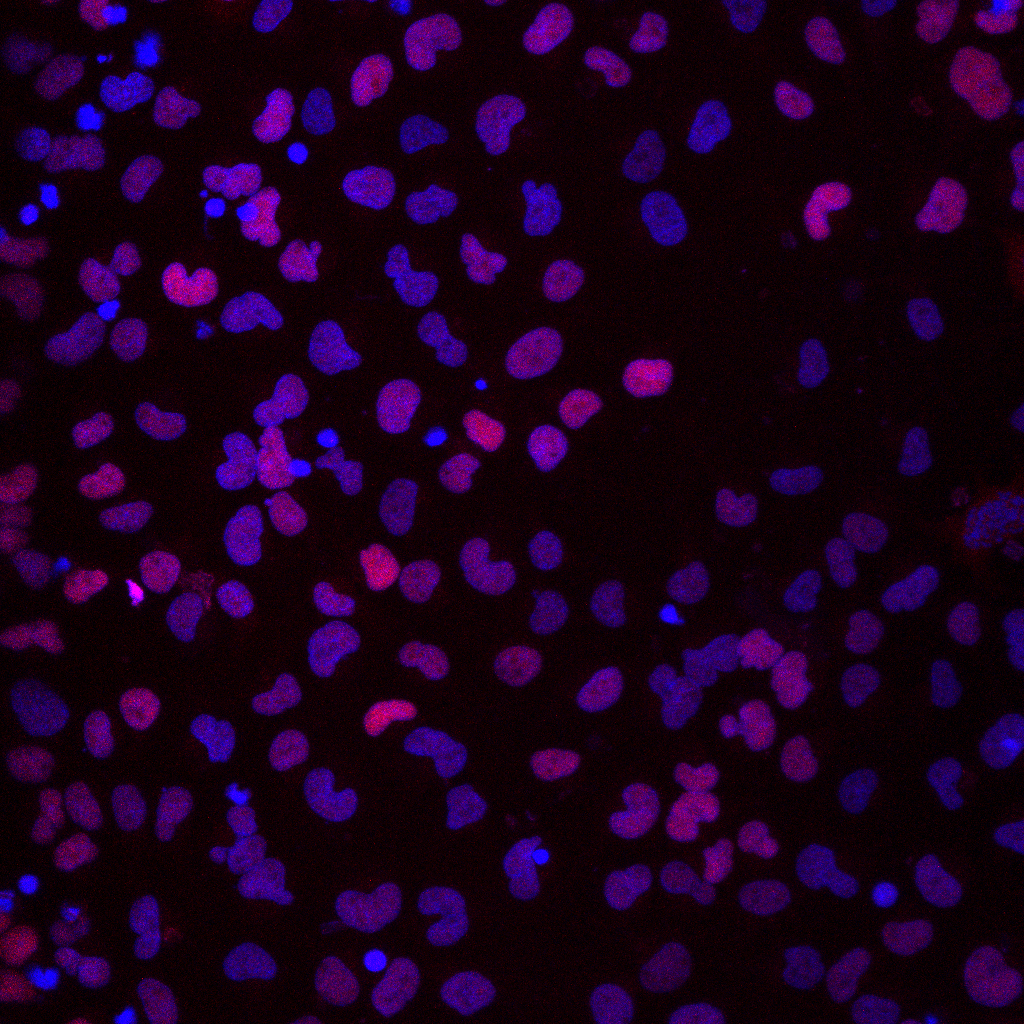

Supplement: Supplementary file 2 — Supplemental Data [file ADVS-13-e05663-s002.zip › advs72932-sup-0001-Data/fig1/fig1b-cryo-six4-dapi.tif]

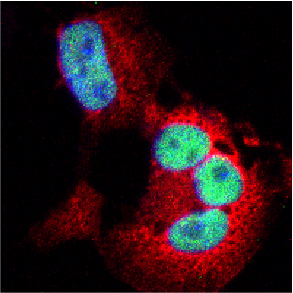

Supplement: Supplementary file 2 — Supplemental Data [file ADVS-13-e05663-s002.zip › advs72932-sup-0001-Data/fig1/fig1b-cryo-sox2-p75.jpg]

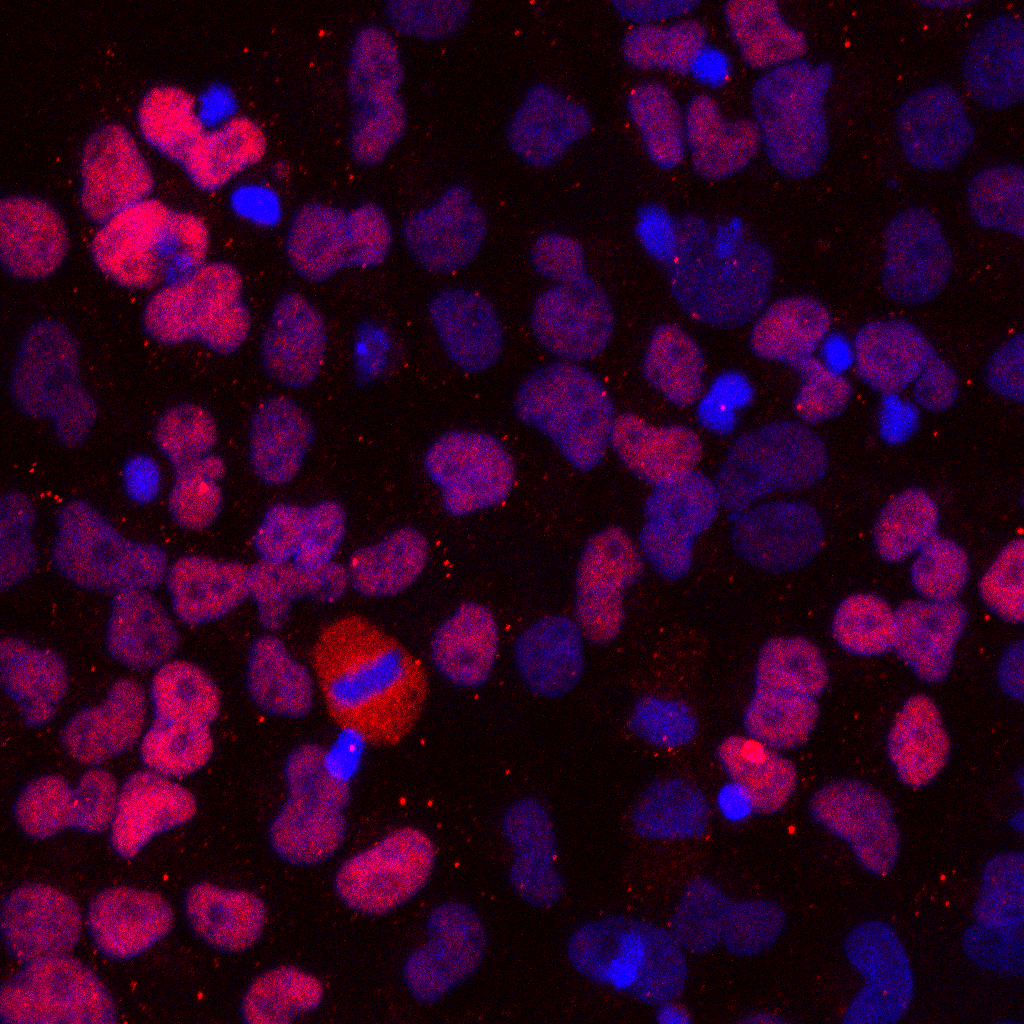

Supplement: Supplementary file 2 — Supplemental Data [file ADVS-13-e05663-s002.zip › advs72932-sup-0001-Data/fig1/fig1b-fresh-six4-dapi.tif]

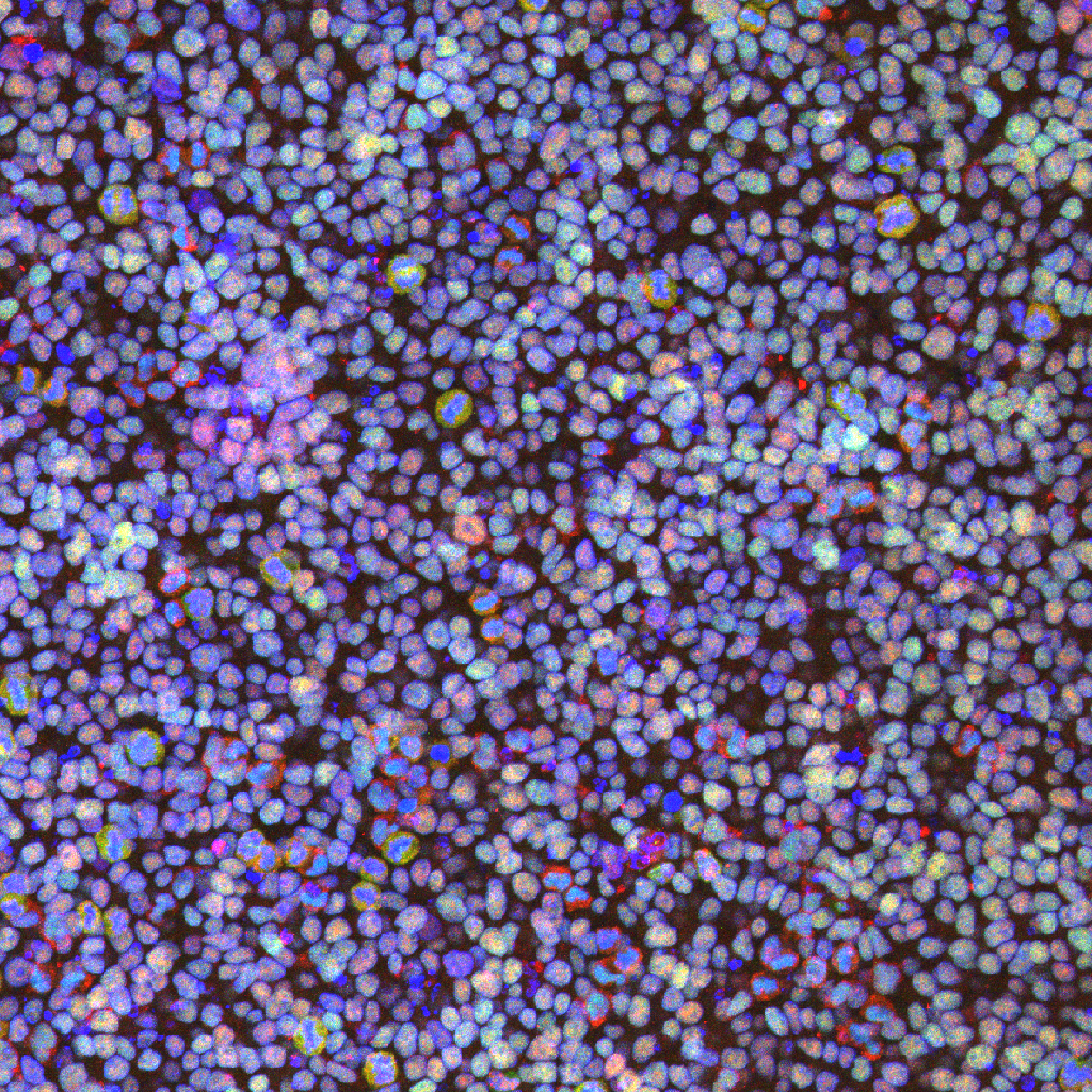

Supplement: Supplementary file 2 — Supplemental Data [file ADVS-13-e05663-s002.zip › advs72932-sup-0001-Data/fig1/fig1b-fresh-sox2-p75-dapi.tif]

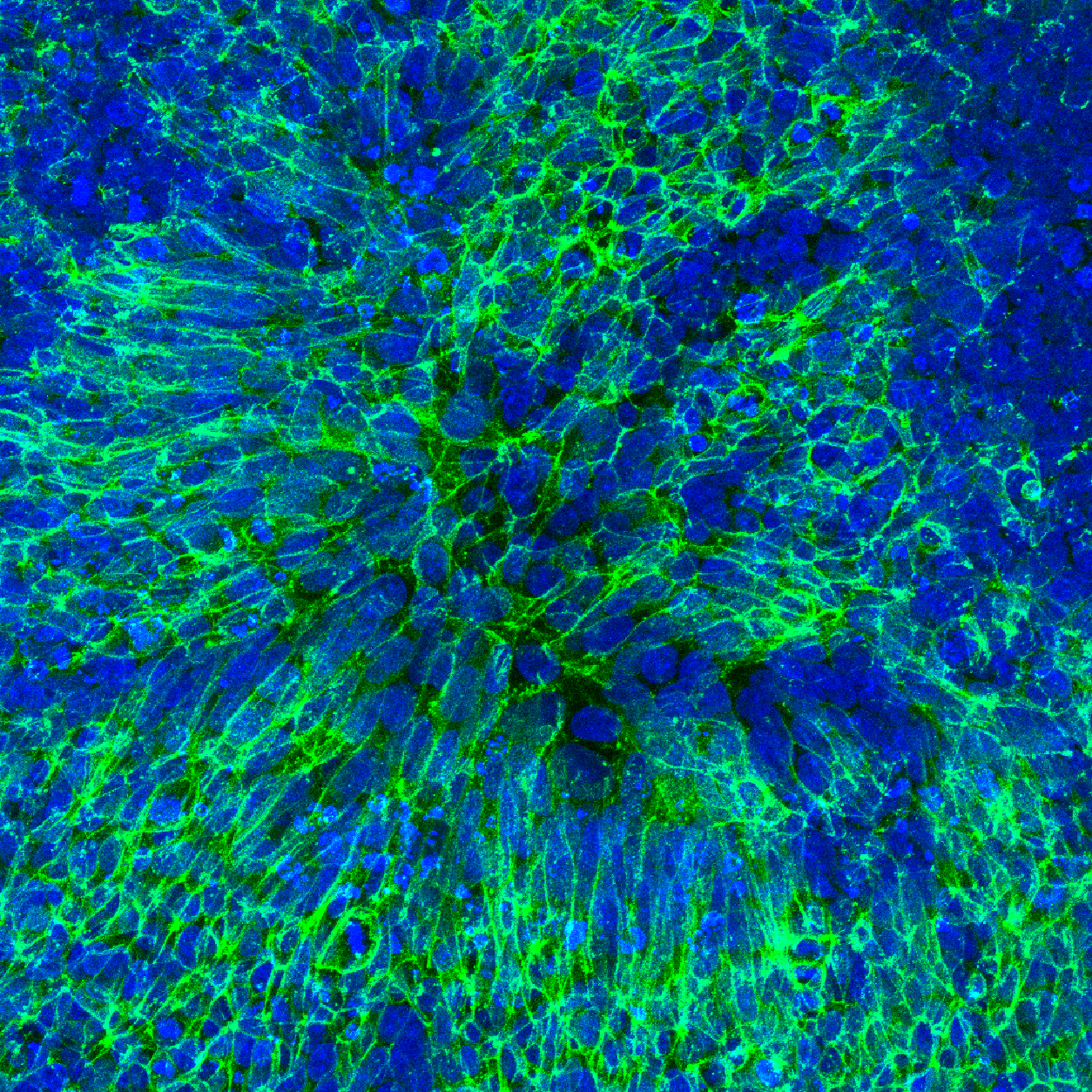

Supplement: Supplementary file 2 — Supplemental Data [file ADVS-13-e05663-s002.zip › advs72932-sup-0001-Data/fig1/fig1b-PPE-ECAD-dapi.tif]

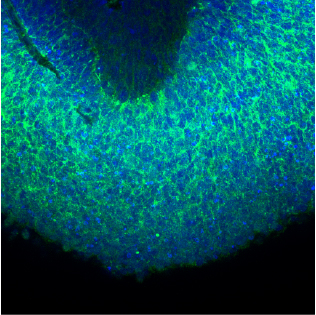

Supplement: Supplementary file 2 — Supplemental Data [file ADVS-13-e05663-s002.zip › advs72932-sup-0001-Data/fig1/fig1c-div15-foxg1-dapi.jpg]

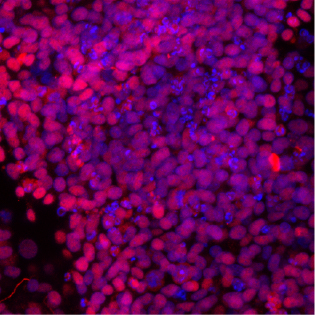

Supplement: Supplementary file 2 — Supplemental Data [file ADVS-13-e05663-s002.zip › advs72932-sup-0001-Data/fig1/fig1c-div15-gata3-dapi.jpg]

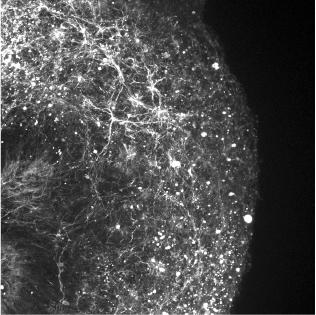

Supplement: Supplementary file 2 — Supplemental Data [file ADVS-13-e05663-s002.zip › advs72932-sup-0001-Data/fig1/fig1c-div15-nestin.jpg]

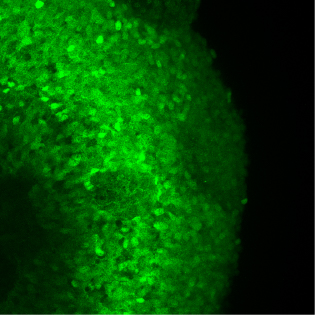

Supplement: Supplementary file 2 — Supplemental Data [file ADVS-13-e05663-s002.zip › advs72932-sup-0001-Data/fig1/fig1c-div15-pax8.jpg]

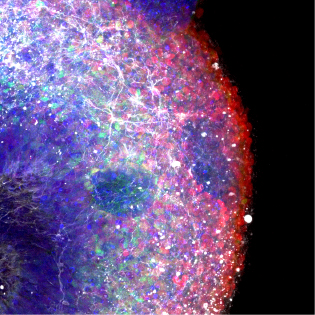

Supplement: Supplementary file 2 — Supplemental Data [file ADVS-13-e05663-s002.zip › advs72932-sup-0001-Data/fig1/fig1c-div15-sox2-pax8-dapi.jpg]

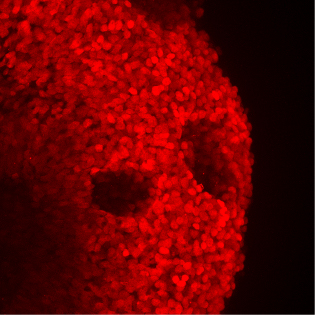

Supplement: Supplementary file 2 — Supplemental Data [file ADVS-13-e05663-s002.zip › advs72932-sup-0001-Data/fig1/fig1c-div15-sox2.jpg]

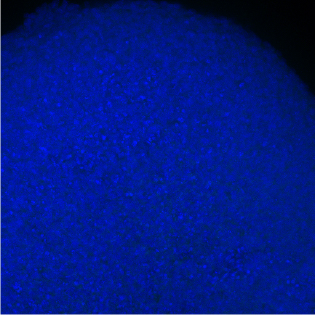

Supplement: Supplementary file 2 — Supplemental Data [file ADVS-13-e05663-s002.zip › advs72932-sup-0001-Data/fig1/fig1c-div9-foxg1-dapi.jpg]

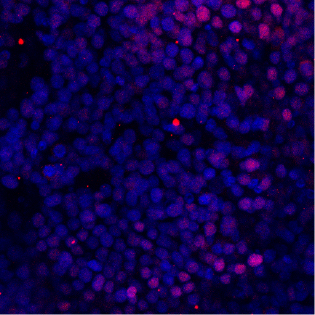

Supplement: Supplementary file 2 — Supplemental Data [file ADVS-13-e05663-s002.zip › advs72932-sup-0001-Data/fig1/fig1c-div9-gata3-dapi.jpg]

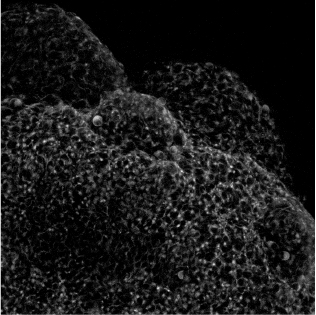

Supplement: Supplementary file 2 — Supplemental Data [file ADVS-13-e05663-s002.zip › advs72932-sup-0001-Data/fig1/fig1c-div9-nestin.jpg]

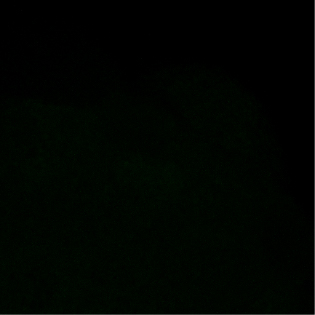

Supplement: Supplementary file 2 — Supplemental Data [file ADVS-13-e05663-s002.zip › advs72932-sup-0001-Data/fig1/fig1c-div9-pax8.jpg]

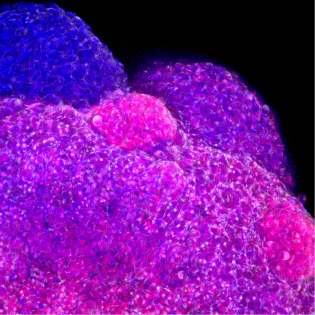

Supplement: Supplementary file 2 — Supplemental Data [file ADVS-13-e05663-s002.zip › advs72932-sup-0001-Data/fig1/fig1c-div9-sox2-pax8-nestin-dapi.jpg]

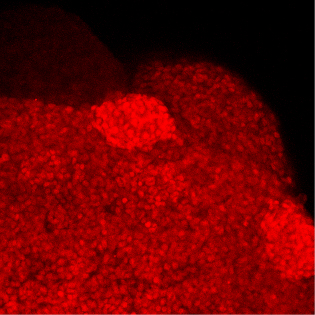

Supplement: Supplementary file 2 — Supplemental Data [file ADVS-13-e05663-s002.zip › advs72932-sup-0001-Data/fig1/fig1c-div9-sox2.jpg]

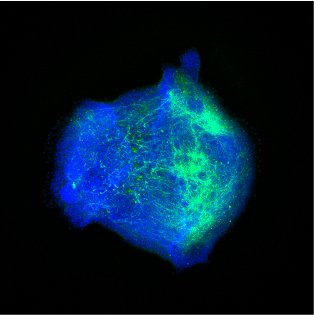

Supplement: Supplementary file 2 — Supplemental Data [file ADVS-13-e05663-s002.zip › advs72932-sup-0001-Data/fig1/fig1d-div18-tuj1-dapi.jpg]

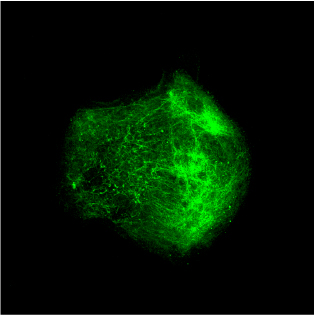

Supplement: Supplementary file 2 — Supplemental Data [file ADVS-13-e05663-s002.zip › advs72932-sup-0001-Data/fig1/fig1d-div18-tuj1.jpg]

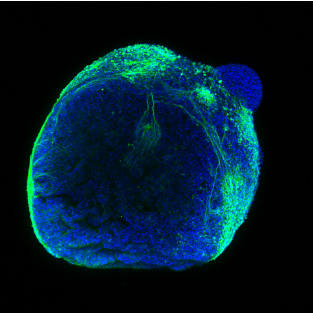

Supplement: Supplementary file 2 — Supplemental Data [file ADVS-13-e05663-s002.zip › advs72932-sup-0001-Data/fig1/fig1d-div25-tuj1-dapi.jpg]

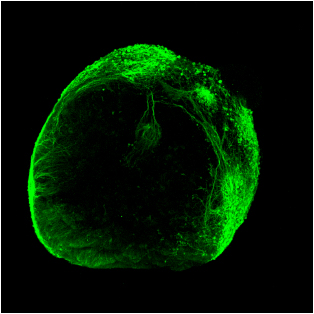

Supplement: Supplementary file 2 — Supplemental Data [file ADVS-13-e05663-s002.zip › advs72932-sup-0001-Data/fig1/fig1d-div25-tuj1.jpg]

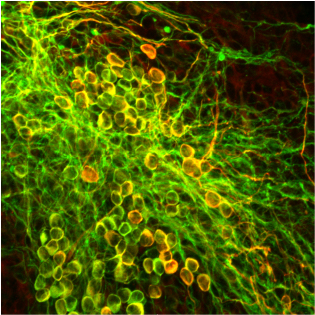

Supplement: Supplementary file 2 — Supplemental Data [file ADVS-13-e05663-s002.zip › advs72932-sup-0001-Data/fig1/fig1e-calb2-tuj1.jpg]

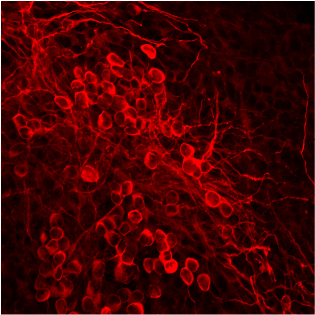

Supplement: Supplementary file 2 — Supplemental Data [file ADVS-13-e05663-s002.zip › advs72932-sup-0001-Data/fig1/fig1e-calb2.jpg]

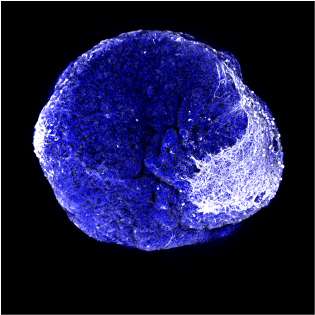

Supplement: Supplementary file 2 — Supplemental Data [file ADVS-13-e05663-s002.zip › advs72932-sup-0001-Data/fig1/fig1e-tuj1-map2.jpg]

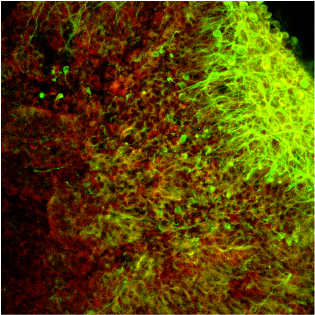

Supplement: Supplementary file 2 — Supplemental Data [file ADVS-13-e05663-s002.zip › advs72932-sup-0001-Data/fig1/fig1e-vglut1-tuj1.jpg]

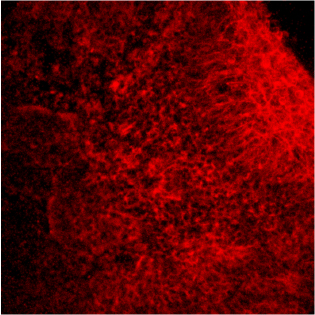

Supplement: Supplementary file 2 — Supplemental Data [file ADVS-13-e05663-s002.zip › advs72932-sup-0001-Data/fig1/fig1e-vglut1.jpg]

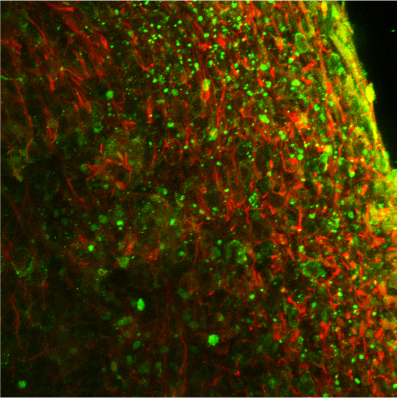

Supplement: Supplementary file 2 — Supplemental Data [file ADVS-13-e05663-s002.zip › advs72932-sup-0001-Data/fig1/fig1f-kcnq4-map2.jpg]

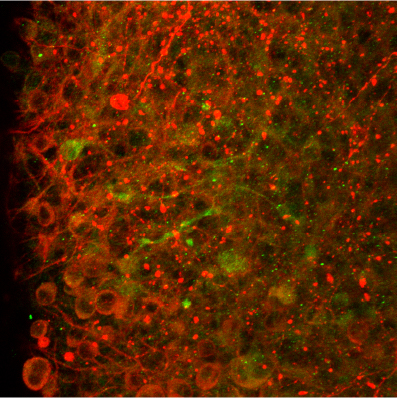

Supplement: Supplementary file 2 — Supplemental Data [file ADVS-13-e05663-s002.zip › advs72932-sup-0001-Data/fig1/fig1f-nav1.3syp.jpg]

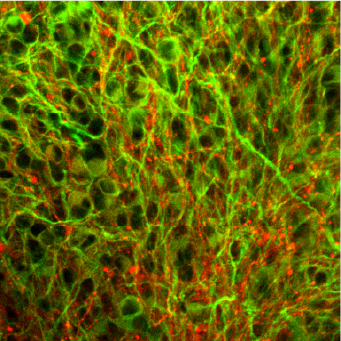

Supplement: Supplementary file 2 — Supplemental Data [file ADVS-13-e05663-s002.zip › advs72932-sup-0001-Data/fig1/fig1f-vglut1-map2.jpg]

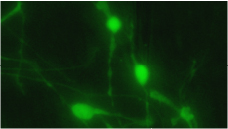

Supplement: Supplementary file 2 — Supplemental Data [file ADVS-13-e05663-s002.zip › advs72932-sup-0001-Data/fig1/fig1g-gcamp.jpg]

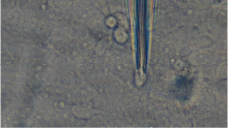

Supplement: Supplementary file 2 — Supplemental Data [file ADVS-13-e05663-s002.zip › advs72932-sup-0001-Data/fig1/fig1g-phase.jpg]

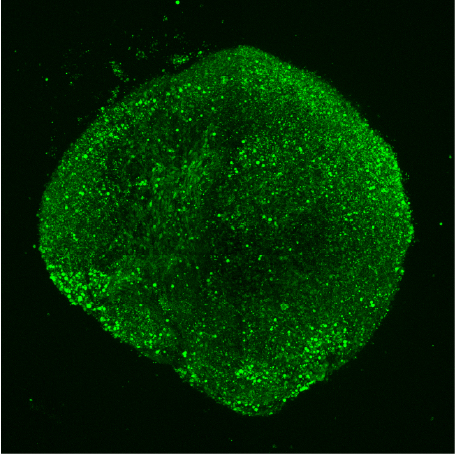

Supplement: Supplementary file 2 — Supplemental Data [file ADVS-13-e05663-s002.zip › advs72932-sup-0001-Data/fig3/fig3a-div100.jpg]

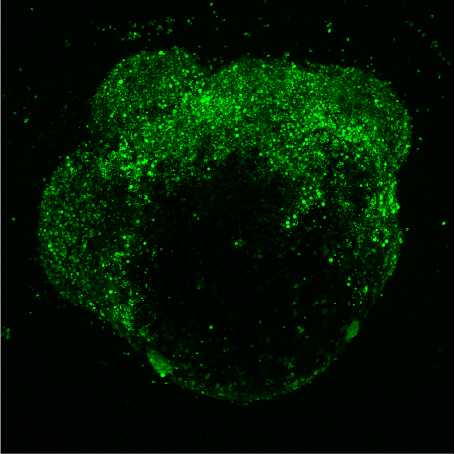

Supplement: Supplementary file 2 — Supplemental Data [file ADVS-13-e05663-s002.zip › advs72932-sup-0001-Data/fig3/fig3a-div25.jpg]

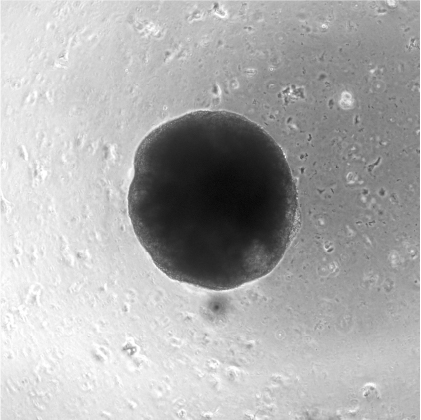

Supplement: Supplementary file 2 — Supplemental Data [file ADVS-13-e05663-s002.zip › advs72932-sup-0001-Data/fig4/fig4b-day0.jpg]

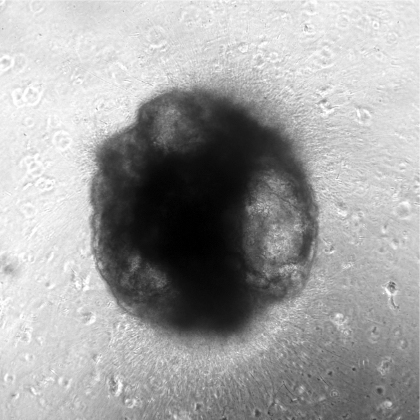

Supplement: Supplementary file 2 — Supplemental Data [file ADVS-13-e05663-s002.zip › advs72932-sup-0001-Data/fig4/fig4b-day7.jpg]

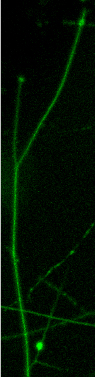

Supplement: Supplementary file 2 — Supplemental Data [file ADVS-13-e05663-s002.zip › advs72932-sup-0001-Data/fig4/fig4c-neurite tip-tuj1.jpg]

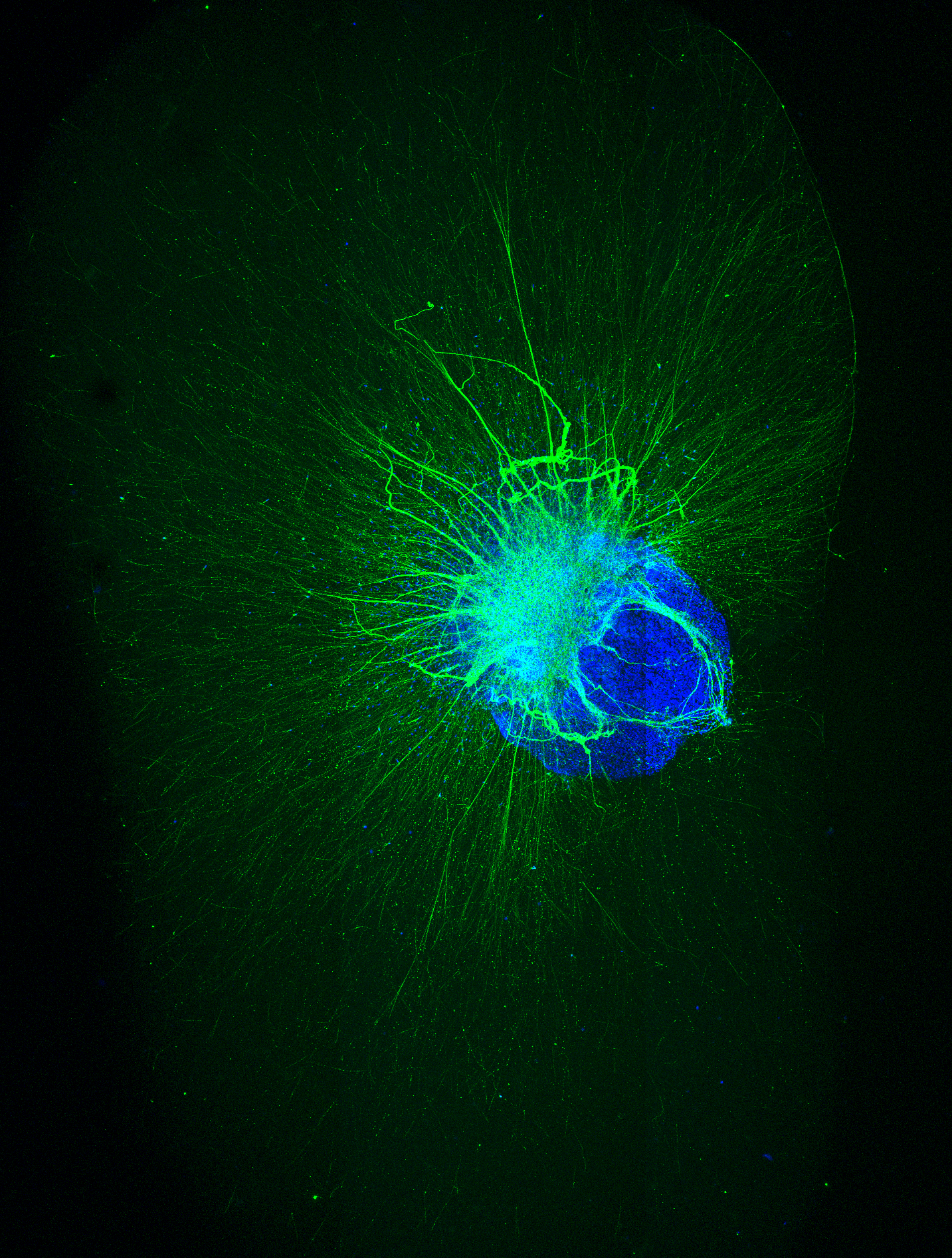

Supplement: Supplementary file 2 — Supplemental Data [file ADVS-13-e05663-s002.zip › advs72932-sup-0001-Data/fig4/fig4c-tuj1-hoe.tif]

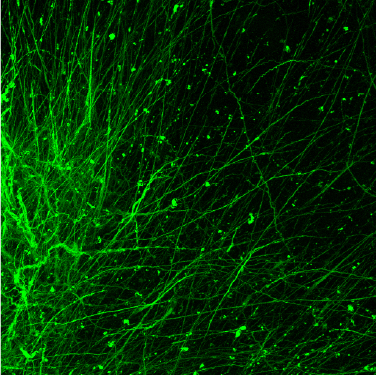

Supplement: Supplementary file 2 — Supplemental Data [file ADVS-13-e05663-s002.zip › advs72932-sup-0001-Data/fig4/fig4d-map2.jpg]

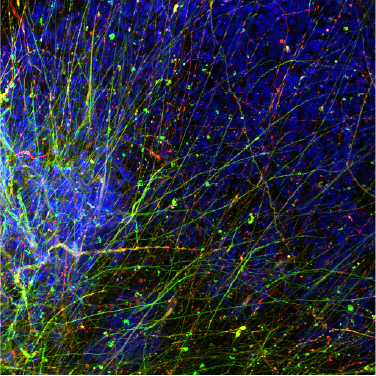

Supplement: Supplementary file 2 — Supplemental Data [file ADVS-13-e05663-s002.zip › advs72932-sup-0001-Data/fig4/fig4d-merge.jpg]

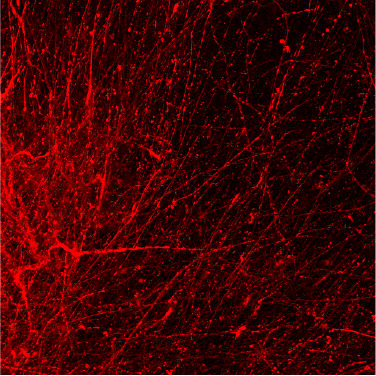

Supplement: Supplementary file 2 — Supplemental Data [file ADVS-13-e05663-s002.zip › advs72932-sup-0001-Data/fig4/fig4d-SYP.jpg]

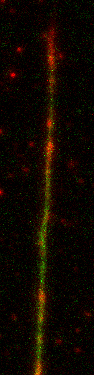

Supplement: Supplementary file 2 — Supplemental Data [file ADVS-13-e05663-s002.zip › advs72932-sup-0001-Data/fig4/fig4d-tip-syp-map2.jpg]

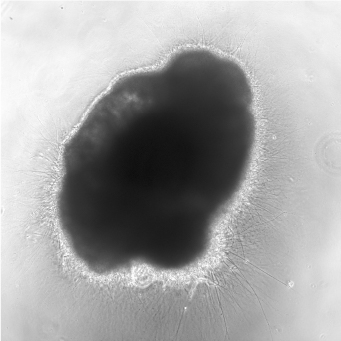

Supplement: Supplementary file 2 — Supplemental Data [file ADVS-13-e05663-s002.zip › advs72932-sup-0001-Data/fig4/fig4e-day0.jpg]

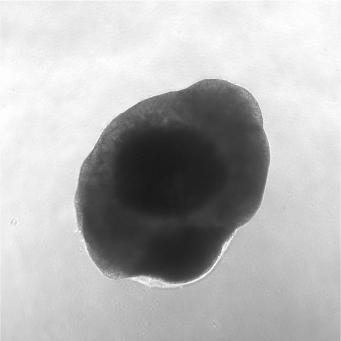

Supplement: Supplementary file 2 — Supplemental Data [file ADVS-13-e05663-s002.zip › advs72932-sup-0001-Data/fig4/fig4e-day7.jpg]

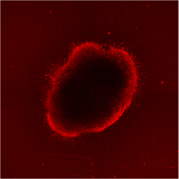

Supplement: Supplementary file 2 — Supplemental Data [file ADVS-13-e05663-s002.zip › advs72932-sup-0001-Data/fig4/fig4f-day1-left.jpg]

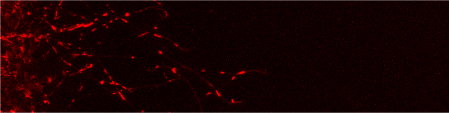

Supplement: Supplementary file 2 — Supplemental Data [file ADVS-13-e05663-s002.zip › advs72932-sup-0001-Data/fig4/fig4f-day1-neurite.jpg]

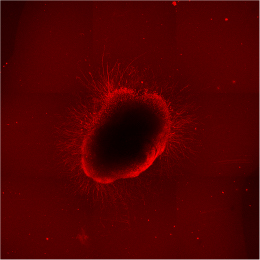

Supplement: Supplementary file 2 — Supplemental Data [file ADVS-13-e05663-s002.zip › advs72932-sup-0001-Data/fig4/fig4f-day3-left.jpg]

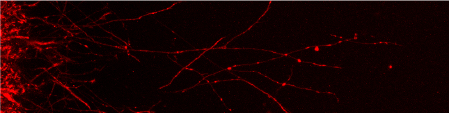

Supplement: Supplementary file 2 — Supplemental Data [file ADVS-13-e05663-s002.zip › advs72932-sup-0001-Data/fig4/fig4f-day3-neurite.jpg]

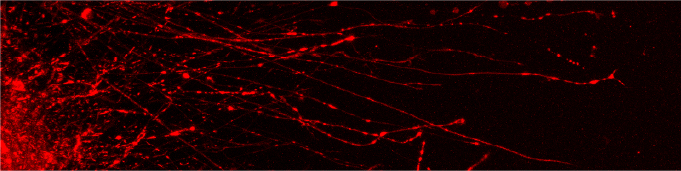

Supplement: Supplementary file 2 — Supplemental Data [file ADVS-13-e05663-s002.zip › advs72932-sup-0001-Data/fig4/fig4f-day5jpg.jpg]

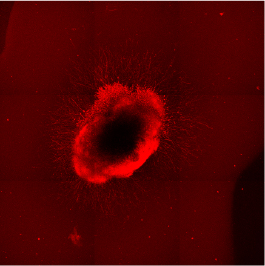

Supplement: Supplementary file 2 — Supplemental Data [file ADVS-13-e05663-s002.zip › advs72932-sup-0001-Data/fig4/fig4f-day7-left.jpg]

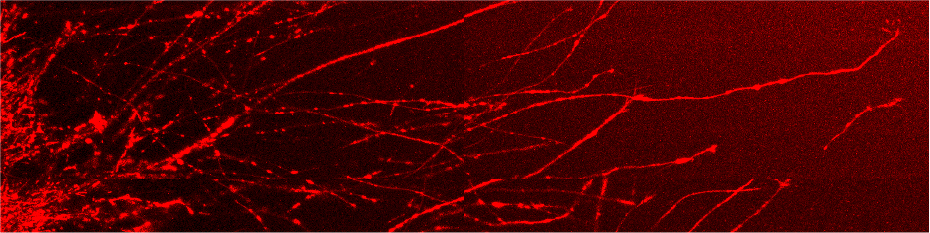

Supplement: Supplementary file 2 — Supplemental Data [file ADVS-13-e05663-s002.zip › advs72932-sup-0001-Data/fig4/fig4f-day7-neurite.jpg]

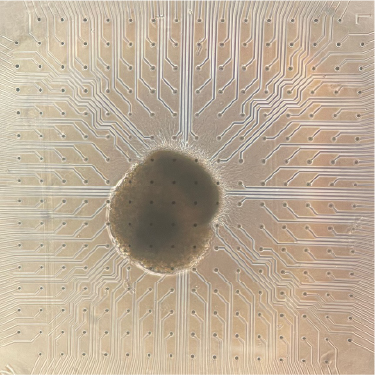

Supplement: Supplementary file 2 — Supplemental Data [file ADVS-13-e05663-s002.zip › advs72932-sup-0001-Data/fig4/fig4h.jpg]

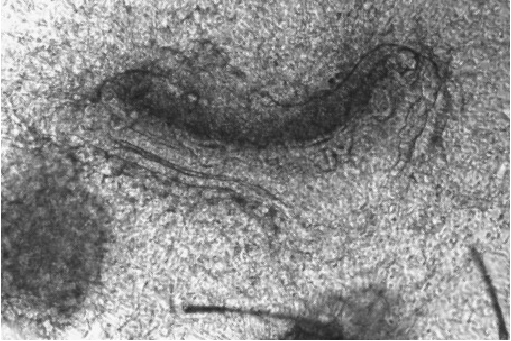

Supplement: Supplementary file 2 — Supplemental Data [file ADVS-13-e05663-s002.zip › advs72932-sup-0001-Data/fig5/Fig 5a-mBM.jpg]

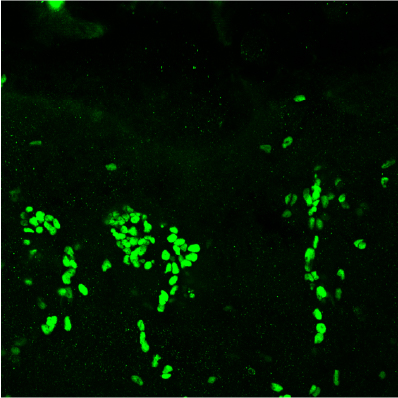

Supplement: Supplementary file 2 — Supplemental Data [file ADVS-13-e05663-s002.zip › advs72932-sup-0001-Data/fig5/Fig 5b-human nuclear.jpg]

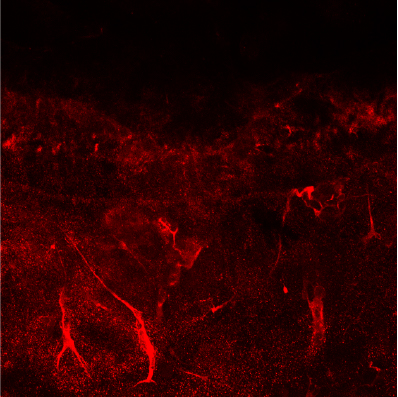

Supplement: Supplementary file 2 — Supplemental Data [file ADVS-13-e05663-s002.zip › advs72932-sup-0001-Data/fig5/Fig 5b-Map2.jpg]

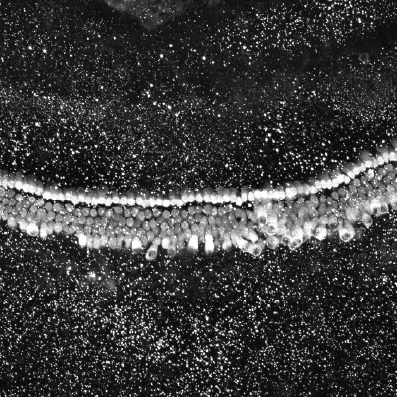

Supplement: Supplementary file 2 — Supplemental Data [file ADVS-13-e05663-s002.zip › advs72932-sup-0001-Data/fig5/Fig 5b-myo7a.jpg]

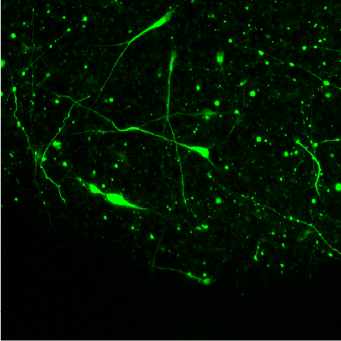

Supplement: Supplementary file 2 — Supplemental Data [file ADVS-13-e05663-s002.zip › advs72932-sup-0001-Data/fig5/Fig 5d-otic spheroid.jpg]

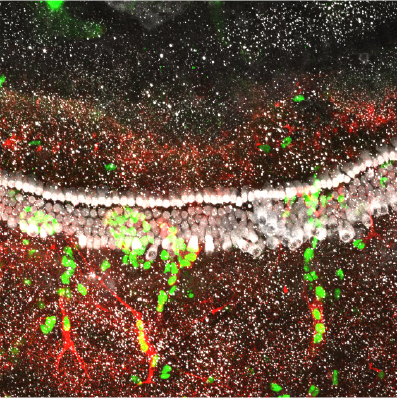

Supplement: Supplementary file 2 — Supplemental Data [file ADVS-13-e05663-s002.zip › advs72932-sup-0001-Data/fig5/Fig5b-merge.jpg]

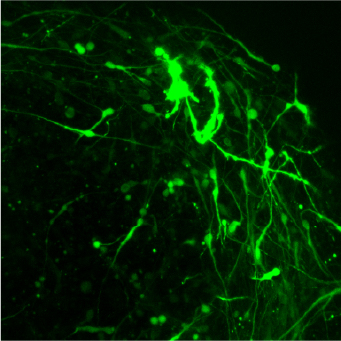

Supplement: Supplementary file 2 — Supplemental Data [file ADVS-13-e05663-s002.zip › advs72932-sup-0001-Data/fig5/Fig5d-hCO.jpg]

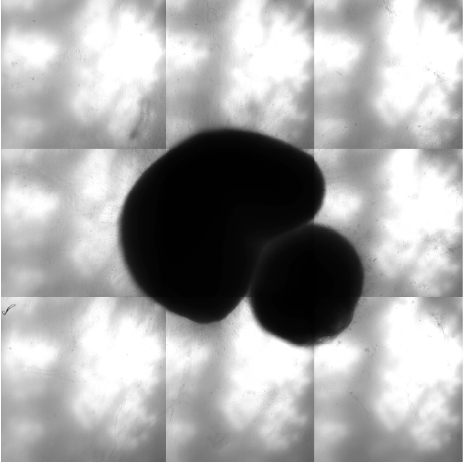

Supplement: Supplementary file 2 — Supplemental Data [file ADVS-13-e05663-s002.zip › advs72932-sup-0001-Data/fig5/Fig5e-BF.jpg]

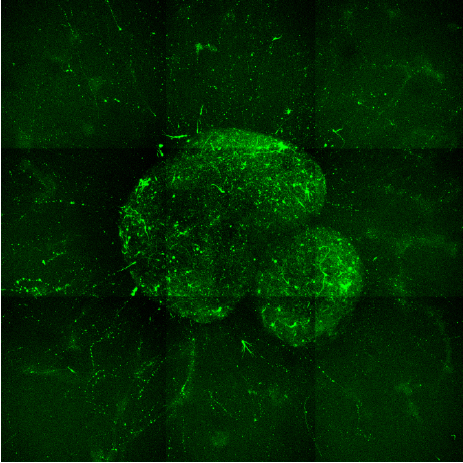

Supplement: Supplementary file 2 — Supplemental Data [file ADVS-13-e05663-s002.zip › advs72932-sup-0001-Data/fig5/Fig5e-Fluo.jpg]

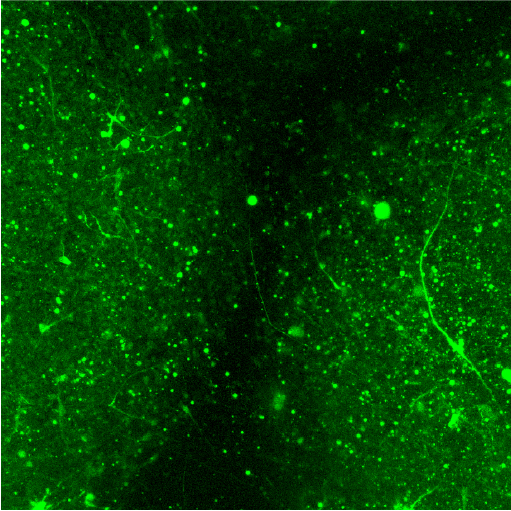

Supplement: Supplementary file 2 — Supplemental Data [file ADVS-13-e05663-s002.zip › advs72932-sup-0001-Data/fig5/Fig5f-21days after fusion-1.jpg]

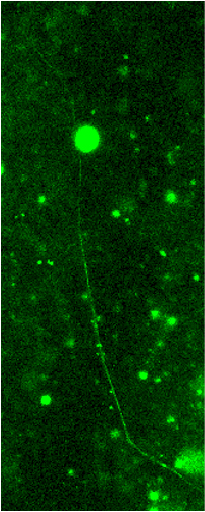

Supplement: Supplementary file 2 — Supplemental Data [file ADVS-13-e05663-s002.zip › advs72932-sup-0001-Data/fig5/Fig5f-21days after fusion-100.jpg]

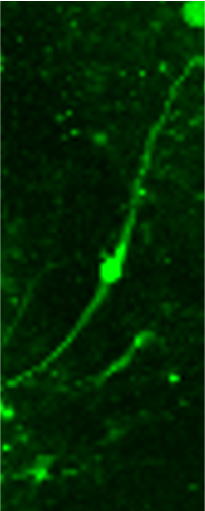

Supplement: Supplementary file 2 — Supplemental Data [file ADVS-13-e05663-s002.zip › advs72932-sup-0001-Data/fig5/Fig5f-42days after fusion--100.jpg]

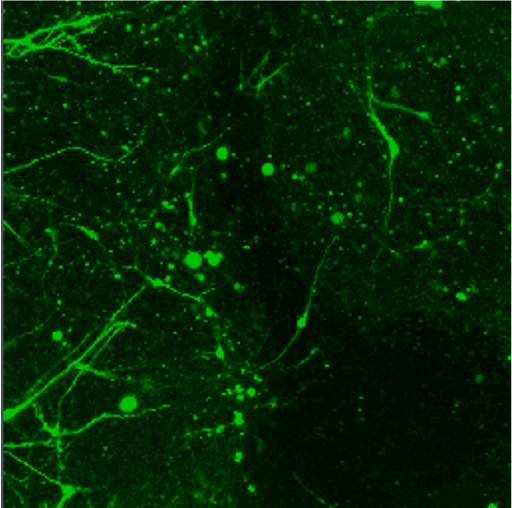

Supplement: Supplementary file 2 — Supplemental Data [file ADVS-13-e05663-s002.zip › advs72932-sup-0001-Data/fig5/Fig5f-42days after fusion-1.jpg]

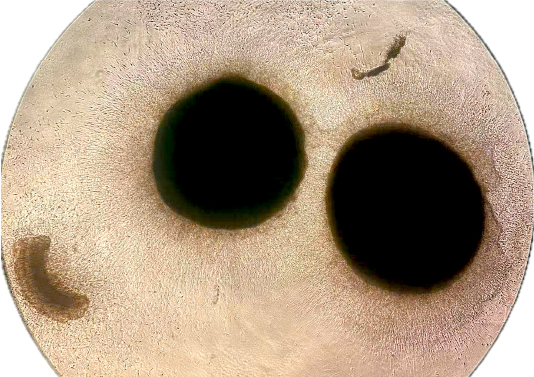

Supplement: Supplementary file 2 — Supplemental Data [file ADVS-13-e05663-s002.zip › advs72932-sup-0001-Data/fig6/fig6a.jpg]

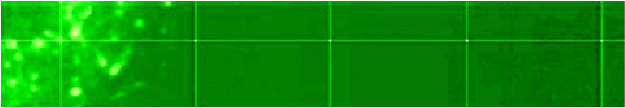

Supplement: Supplementary file 2 — Supplemental Data [file ADVS-13-e05663-s002.zip › advs72932-sup-0001-Data/fig6/fig6b-left-0h.jpg]

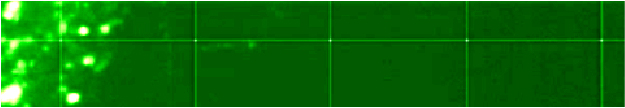

Supplement: Supplementary file 2 — Supplemental Data [file ADVS-13-e05663-s002.zip › advs72932-sup-0001-Data/fig6/fig6b-left-12h.jpg]

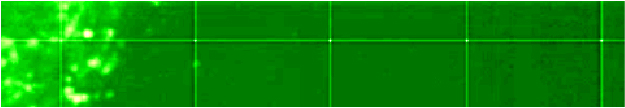

Supplement: Supplementary file 2 — Supplemental Data [file ADVS-13-e05663-s002.zip › advs72932-sup-0001-Data/fig6/fig6b-left-4h.jpg]

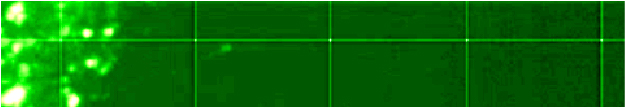

Supplement: Supplementary file 2 — Supplemental Data [file ADVS-13-e05663-s002.zip › advs72932-sup-0001-Data/fig6/fig6b-left-8h.jpg]

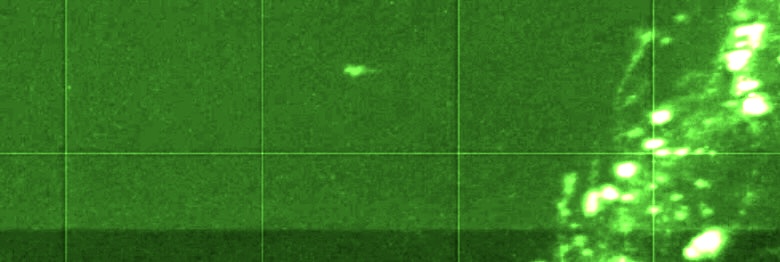

Supplement: Supplementary file 2 — Supplemental Data [file ADVS-13-e05663-s002.zip › advs72932-sup-0001-Data/fig6/fig6b-tohco-0h.jpg]

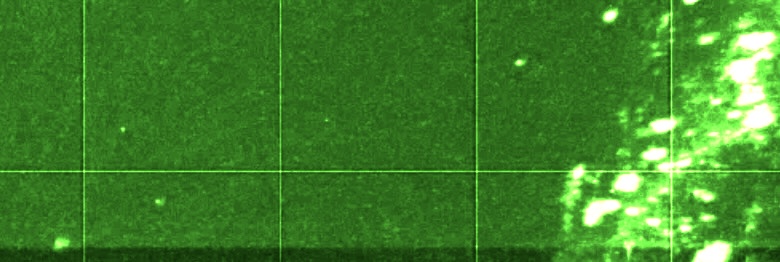

Supplement: Supplementary file 2 — Supplemental Data [file ADVS-13-e05663-s002.zip › advs72932-sup-0001-Data/fig6/fig6b-tohco-12h.jpg]

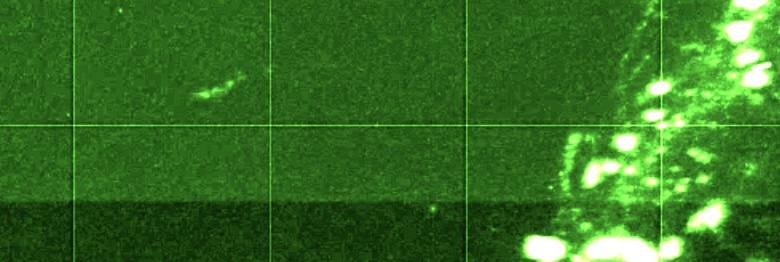

Supplement: Supplementary file 2 — Supplemental Data [file ADVS-13-e05663-s002.zip › advs72932-sup-0001-Data/fig6/fig6b-tohco-4h.jpg]

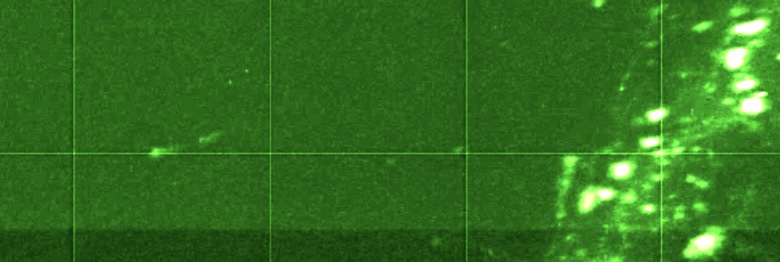

Supplement: Supplementary file 2 — Supplemental Data [file ADVS-13-e05663-s002.zip › advs72932-sup-0001-Data/fig6/fig6b-tohco-8h.jpg]

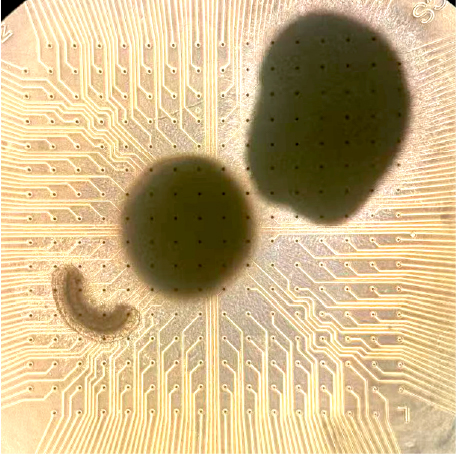

Supplement: Supplementary file 2 — Supplemental Data [file ADVS-13-e05663-s002.zip › advs72932-sup-0001-Data/fig6/fig6c.jpg]

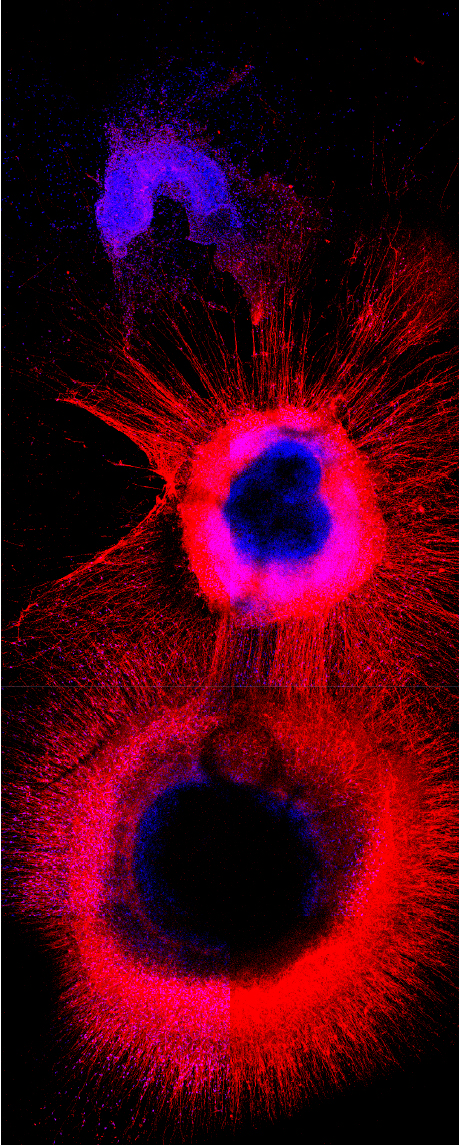

Supplement: Supplementary file 2 — Supplemental Data [file ADVS-13-e05663-s002.zip › advs72932-sup-0001-Data/fig6/fig6g-left.jpg]

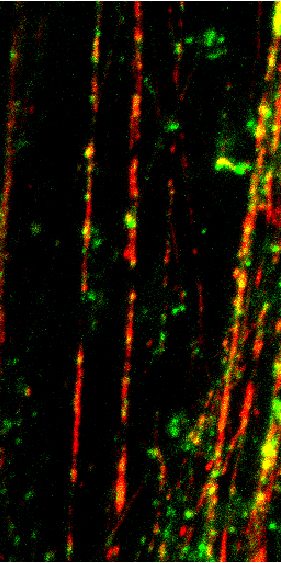

Supplement: Supplementary file 2 — Supplemental Data [file ADVS-13-e05663-s002.zip › advs72932-sup-0001-Data/fig6/fig6g-neurite-down.jpg]

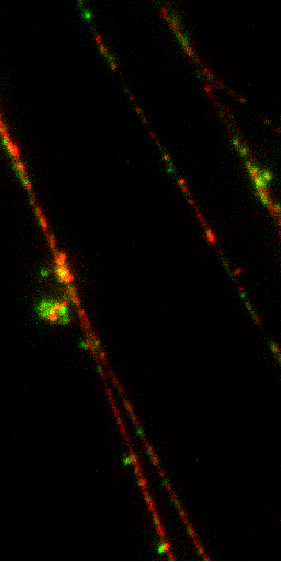

Supplement: Supplementary file 2 — Supplemental Data [file ADVS-13-e05663-s002.zip › advs72932-sup-0001-Data/fig6/fig6g-neurite-up.jpg]

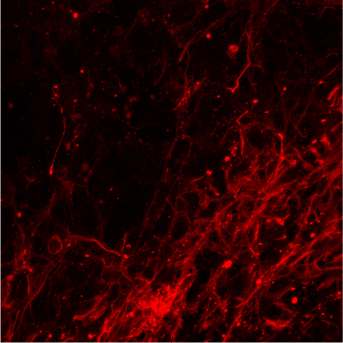

Supplement: Supplementary file 2 — Supplemental Data [file ADVS-13-e05663-s002.zip › advs72932-sup-0001-Data/fig6/fig6h-control-map2.jpg]

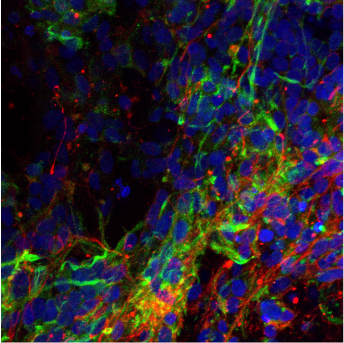

Supplement: Supplementary file 2 — Supplemental Data [file ADVS-13-e05663-s002.zip › advs72932-sup-0001-Data/fig6/fig6h-control-merge.jpg]

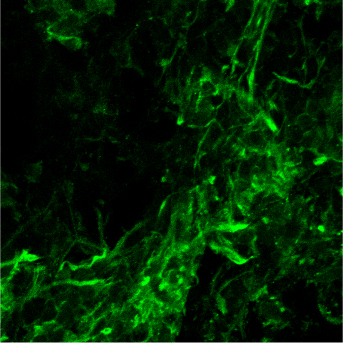

Supplement: Supplementary file 2 — Supplemental Data [file ADVS-13-e05663-s002.zip › advs72932-sup-0001-Data/fig6/fig6h-control-sema6d.jpg]

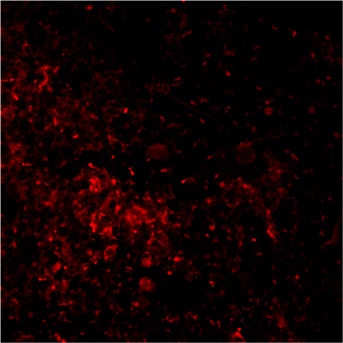

Supplement: Supplementary file 2 — Supplemental Data [file ADVS-13-e05663-s002.zip › advs72932-sup-0001-Data/fig6/fig6h-sirna-map2.jpg]

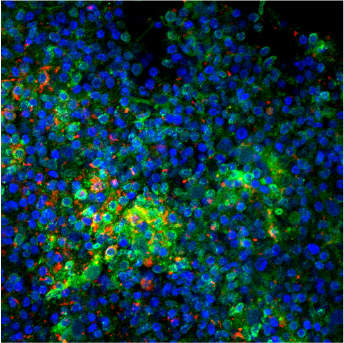

Supplement: Supplementary file 2 — Supplemental Data [file ADVS-13-e05663-s002.zip › advs72932-sup-0001-Data/fig6/fig6h-sirna-merge.jpg]

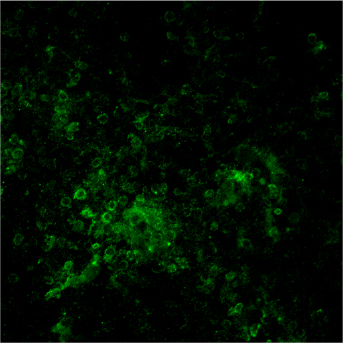

Supplement: Supplementary file 2 — Supplemental Data [file ADVS-13-e05663-s002.zip › advs72932-sup-0001-Data/fig6/fig6h-sirna-sema6d.jpg]

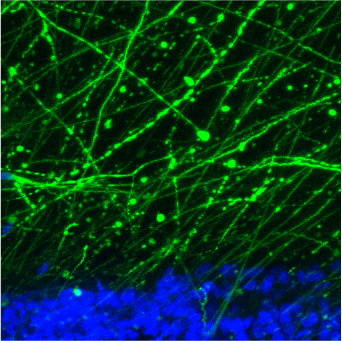

Supplement: Supplementary file 2 — Supplemental Data [file ADVS-13-e05663-s002.zip › advs72932-sup-0001-Data/fig6/fig6i-control.jpg]

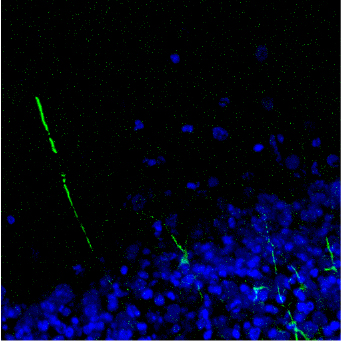

Supplement: Supplementary file 2 — Supplemental Data [file ADVS-13-e05663-s002.zip › advs72932-sup-0001-Data/fig6/fig6i-sirna.jpg]

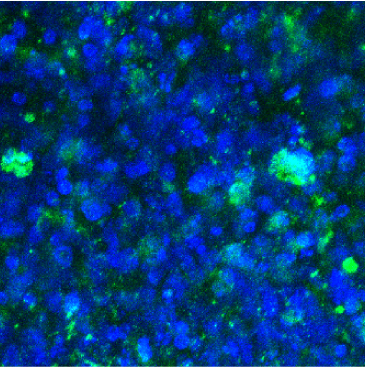

Supplement: Supplementary file 2 — Supplemental Data [file ADVS-13-e05663-s002.zip › advs72932-sup-0001-Data/fig7/Fig7b-10uM cisplatin-CC3+DAPI.jpg]

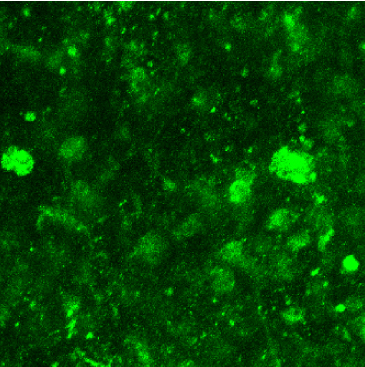

Supplement: Supplementary file 2 — Supplemental Data [file ADVS-13-e05663-s002.zip › advs72932-sup-0001-Data/fig7/Fig7b-10uM cisplatin-CC3.jpg]

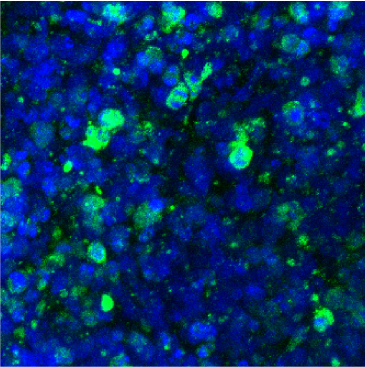

Supplement: Supplementary file 2 — Supplemental Data [file ADVS-13-e05663-s002.zip › advs72932-sup-0001-Data/fig7/Fig7b-50uM cisplatin-CC3+DAPI.jpg]

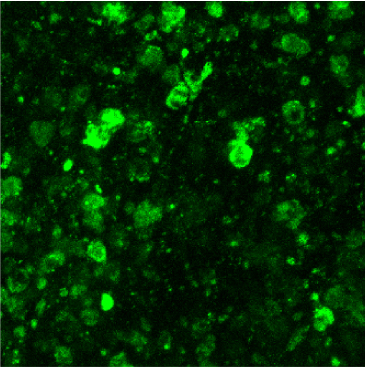

Supplement: Supplementary file 2 — Supplemental Data [file ADVS-13-e05663-s002.zip › advs72932-sup-0001-Data/fig7/Fig7b-50uM cisplatin-CC3.jpg]

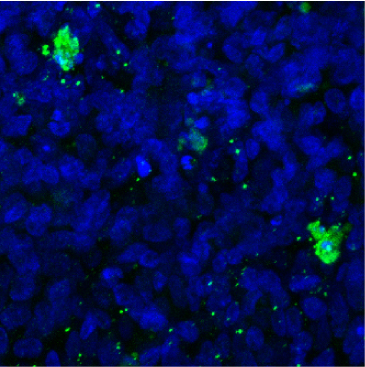

Supplement: Supplementary file 2 — Supplemental Data [file ADVS-13-e05663-s002.zip › advs72932-sup-0001-Data/fig7/Fig7b-control-CC3+DAPI.jpg]

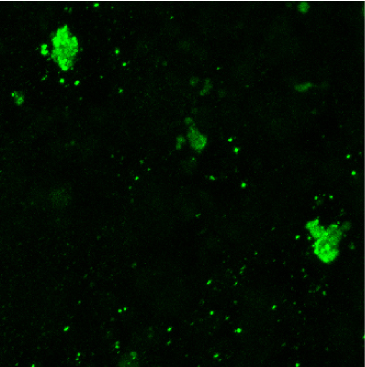

Supplement: Supplementary file 2 — Supplemental Data [file ADVS-13-e05663-s002.zip › advs72932-sup-0001-Data/fig7/Fig7b-control-CC3.jpg]

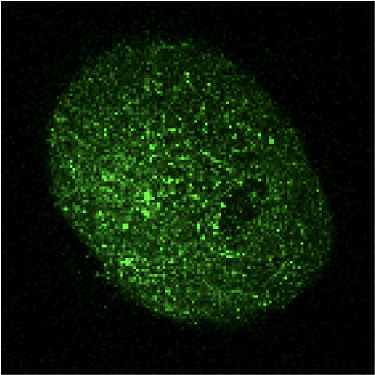

Supplement: Supplementary file 2 — Supplemental Data [file ADVS-13-e05663-s002.zip › advs72932-sup-0001-Data/fig7/Fig7c-10uM cisplatin.jpg]

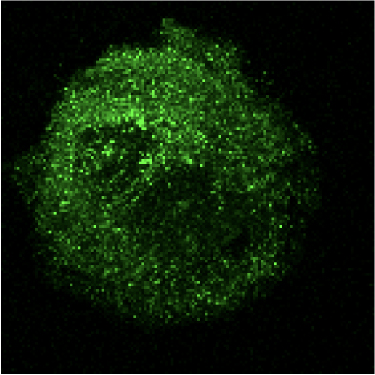

Supplement: Supplementary file 2 — Supplemental Data [file ADVS-13-e05663-s002.zip › advs72932-sup-0001-Data/fig7/Fig7c-50uM cisplatin.jpg]

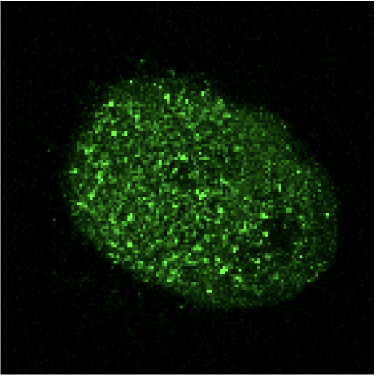

Supplement: Supplementary file 2 — Supplemental Data [file ADVS-13-e05663-s002.zip › advs72932-sup-0001-Data/fig7/Fig7c-control.jpg]

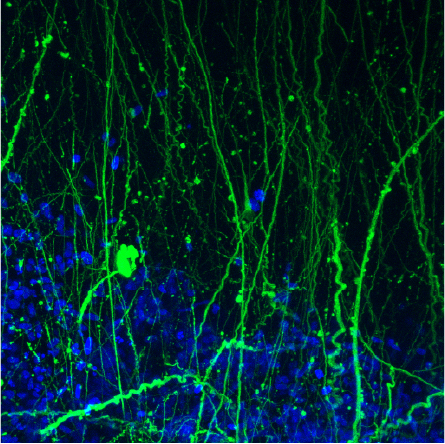

Supplement: Supplementary file 2 — Supplemental Data [file ADVS-13-e05663-s002.zip › advs72932-sup-0001-Data/fig7/Fig7h-10uM cisplatin.jpg]

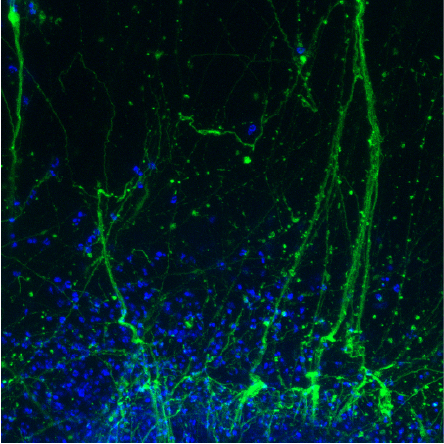

Supplement: Supplementary file 2 — Supplemental Data [file ADVS-13-e05663-s002.zip › advs72932-sup-0001-Data/fig7/Fig7h-50uM cisplatin.jpg]

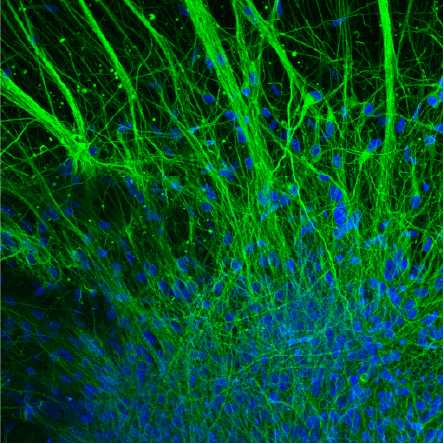

Supplement: Supplementary file 2 — Supplemental Data [file ADVS-13-e05663-s002.zip › advs72932-sup-0001-Data/fig7/Fig7h-control.jpg]

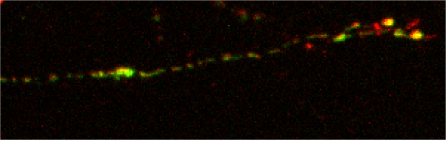

Supplement: Supplementary file 2 — Supplemental Data [file ADVS-13-e05663-s002.zip › advs72932-sup-0001-Data/fig7/Fig7i-10uM cisplatin-SYP+TUJ1.jpg]

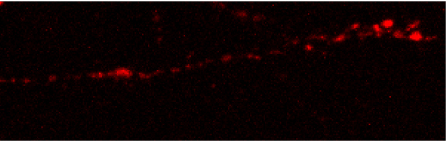

Supplement: Supplementary file 2 — Supplemental Data [file ADVS-13-e05663-s002.zip › advs72932-sup-0001-Data/fig7/Fig7i-10uM cisplatin-SYP.jpg]
